# Annotated check list of the Pyraloidea (Lepidoptera) of America North of Mexico

**DOI:** 10.3897/zookeys.535.6086

**Published:** 2015-11-13

**Authors:** Brian G. Scholtens, M. Alma Solis

**Affiliations:** 1 BGS, Biology Department, College of Charleston, Charleston, SC, USA 29424; 2 MAS, Systematic Entomology Laboratory, USDA, National Museum of Natural History, P.O. Box 37012, MRC 168, Washington, DC USA 20013-7012

**Keywords:** Canada, United States, Pyralidae, Crambidae, faunistics, introduced species, distribution

## Abstract

An annotated check list of Pyraloidea of North America north of Mexico is presented, including 861 Crambidae and 681 Pyralidae with 1542 total species. It includes all new species described, tropical species with new records in the United States, and species introduced from Europe and Asia since 1983. The Notes section provides the seminal citations, data and/or commentary to all changes since 1983 for easy and future reference. In addition, this list proposes seven new generic combinations, the transfer of a phycitine species, Salebria nigricans (Hulst), to Epipaschiinae and its **syn. n.** with Pococera fuscolotella (Ragonot), and three new records for the United States. Purposefully, no new taxa are described here, but we found a gradual increase of 10% in the number of species described since 1983. Finally, we also include a list of thirteen species not included or removed from the MONA list. Many higher-level changes have occurred since 1983 and the classification is updated to reflect research over the last 30 years, including exclusion of Thyrididae and Hyblaeidae from the superfamily and recognition of Crambidae and Pyralidae as separate families. The list includes multiple changes to subfamilies based on morphology such as the synonymization of the Dichogamini with the Glaphyriinae, but also incorporating recent molecular phylogenetic results such as the synonymization of the Evergestinae with the Glaphyriinae.

## Introduction

This check list is an update of the Pyraloidea by Munroe (1983) and Klots (1983) in Hodges et al. (1983) (hereafter referred to as the MONA check list) that is now over 30 years old. An updated list of Pyraloidea of North America north of Mexico is greatly needed because there have been many new species described, higher-level taxonomic and nomenclatural changes, additions and deletions, new synonymies and combinations, and new introductions to the fauna since 1983.

The additional species included in this check list are from three primary sources: newly described species (Table 1), tropical species newly reported from the United States, and new introductions from Europe and Asia (Table 2). Species listed here are mostly established, breeding populations, but also include some strays and recent introductions which may not, ultimately, become established. This check list is also highly influenced by the publication in 1995 of the first check list of Neotropical pyraloids (hereafter known as the NTCL or Neotropical check list) (Munroe 1995a,b; Solis 1995; Solis, Becker and Munroe 1995; M. Shaffer and Solis 1995; J. Shaffer 1995). This list included many changes to the MONA check list because it included species in America north of Mexico (indicated with an * in the NTCL) that were described mostly from U.S. states that border Mexico and could possibly extend south into the Neotropical region. In addition, the results of studying and comparing type specimens over many years by the authors resulted in many new combinations and synonymies that affected American pyraloid nomenclature.

**Table 1.** Faunal additions as new species.

Species	Reference	Note	Species	Reference	Note
Anania tennesseensis	Yang et al. 2012	10	Dioryctria inyoensis	Neunzig 2003	278
Herpetogamma sphingealis	Handfield and Handfield 2011	40	Dioryctria sierra	Neunzig 2003	278
Palpita maritima	Sullivan and Solis 2013	72	Dioryctria resinosella	Mutuura 1982	279
Schacontia themis	Goldstein et al. 2013	102	Dioryctria ebeli	Mutuura and Munroe 1979	281
Eoreuma arenella	Blanchard and Knudson 1983a	127	Dioryctria merkeli	Mutuura and Munroe 1979	281
Diatraea mitteri	Solis et al. 2015	128	Dioryctria yatesi	Mutuura and Munroe 1979	281
Neodactria glenni	Landry and Metzler 2002	142	Dioryctria westerlandi	Donahue and Neunzig 2002	282
Neodactria daemonis	Landry and Brown 2005	143	Dioryctria fordi	Donahue and Neunzig 2002	282
Neodactria oktibbeha	Landry and Brown 2005	143	Dioryctria durangoensis	Mutuura and Neunzig 1986	283
Neodactria cochisensis	Landry and Albu 2012	144	Dioryctria caesirufella	Blanchard and Knudson 1983c	284
La cerveza	Landry 1995	148	Dioryctria taedivorella	Neunzig and Leidy 1989	285
Parapediasia torquatella	Landry 1995	151	Lipographis unicolor	Ferris 2012b	289
Almita portalia	Landry 1995	152	Acroncosa minima	Neunzig 2003	294
Almita texana	Landry 1995	152	Passadenoides donahuei	Neunzig 2003	296
Chrysendeton nigrescens	Heppner 1991	176	Passadenoides pullus	Neunzig 2003	296
Petrophila heppneri	Blanchard and Knudson 1983a	178	Passadenoides montanus	Ferris 2004	297
Paragalasa exospinalis	Solis et al. 2013	191	Ancylosis balconiensis	Blanchard and Knudson 1985c	308
Penthesilea sacculalis baboquivariensis	Solis et al. 2013	194	Palatka powelli	Neunzig and Solis 1996	311
Arta brevivalvalis	Solis et al. 2013	203	Anderida peorinella	Blanchard and Knudson 1985b	312
Heliades lindae	Solis et al. 2013	205	Mescinia texanica	Neunzig 1997	317
Acrobasis texana	Neunzig 1986	221	Homoeosoma phaeoboreas	Goodson and Neunzig 1993	320
Acrobasis juglanivorella	Neunzig 1986	221	Homoeosoma asylonnastes	Goodson and Neunzig 1993	320
Acrobasis caulivorella	Neunzig 1986	221	Homoeosoma oxycercus	Goodson and Neunzig 1993	320
Acrobasis kylesi	Neunzig 1986	221	Homoeosoma ammonastes	Goodson and Neunzig 1993	320
Crocidomera imitata	Neunzig 1990	232	Homoeosoma pedionnastes	Goodson and Neunzig 1993	320
Myelopsoides venustus	Neunzig 1998	235	Homoeosoma eremophasma	Goodson and Neunzig 1993	320
Pima fergusoni	Neunzig 2003	245	Homoeosoma ardaloniphas	Goodson and Neunzig 1993	320
Pimodes caliginosus	Neunzig 2003	246	Homoeosoma parvalbum	Blanchard and Knudson 1985c	322
Ambesa dentifera	Neunzig 2003	249	Homoeosoma nanophasma	Neunzig 2003	324
Catastia subactualis	Neunzig 2003	250	Laetilia cinerosella	Neunzig 1997	330
Salebriaria borealis	Neunzig 1988	252	Laetilia bellivorella	Neunzig 1997	330
Salebriaria chisosensis	Neunzig 1988	252	Baphala phaeolella	Neunzig 1997	333
Salebriaria fasciata	Neunzig 1988	252	Zophodia multistriatella	Blanchard and Knudson 1981	334
Salebriaria rufimaculatella	Neunzig 1988	252	Melitara texana	Neunzig 1997	336
Salebriaria bella	Neunzig 1988	252	Melitara apicigrammella	Blanchard and Knudson 1985c	338
Salebriaria grandidentalis	Neunzig 1988	252	Alberada californiensis	Neunzig 1997	340
Salebriaria equivoca	Neunzig 1988	252	Alberada franclemonti	Neunzig 1997	340
Salebriaria integra	Neunzig 1988	252	Alberada candida	Neunzig 1997	340
Salebriaria maximella	Neunzig 1988	252	Rumatha jacumba	Neunzig 1997	342
Salebriaria simpliciella	Neunzig 1988	252	Selga californica	Neunzig 1990	347
Salebriaria carolynae	Neunzig 1988	252	Euzophera habrella	Neunzig 1990	350
Salebriaria squamopalpiella	Neunzig 1988	252	Euzophera vinnulella	Neunzig 1990	350
Salebriaria roseopunctella	Neunzig 2003	253	Ephestiodes monticolus	Neunzig 1990	351
Salebriaria kanawha	Neunzig 2003	253	Ephestiodes griseus	Neunzig 1990	351
Salebriaria floridana	Neunzig 2003	253	Moodna pallidostrinella	Neunzig 1990	356
Salebriaria pallidella	Neunzig 2003	253	Vitula aegerella	Neunzig 1990	360
Salebriaria fergusonella	Blanchard and Knudson 1983b	254	Vitula insula	Neunzig 1990	360
Quasisalebria atratella	Blanchard and Knudson 1985b	255	Vitula coconinoana	Neunzig 1990	360
Quasisalebria occidentalis	Neunzig 1988	258	Volatica gallivorella	Neunzig 1990	361
Ortholepis baloghi	Neunzig 2003	259	Caudellia floridensis	Neunzig 1990	363
Meroptera anaimella	Blanchard and Knudson 1985c	260	Sosipatra proximanthophila	Neunzig 1990	364
Meroptera nevadensis	Neunzig 2003	261	Sosipatra knudsoni	Neunzig 1990	364
Sciota quasisubfuscella	Neunzig 2003	266	Heinrichiessa sanpetella	Neunzig 1990	365
Sciota californiana	Neunzig 2003	266	Ribua droozi	Neunzig 1990	366
Pyla araeneola	Balogh and Wilterding 1998	272	Ephestia columbiella	Neunzig 1990	371
Pyla westerlandi	Wilterding and Balogh 2002	274	Uncitruncata leuschneri	Neunzig 2000	374
Pyla longispina	Neunzig 2003	275	Peoria padreella	Blanchard 1980	378
Pyla serrata	Neunzig 2003	275	Peoria insularis	Shaffer 2003	379
Utah sanrafaelensis	Ferris 2012a	276	Atascosa heitzmani	Shaffer 1980	380
Dioryctria hodgesi	Neunzig 2003	278	Oneida grisiella	Solis 1991	393
Dioryctria muricativorella	Neunzig 2003	278	Neodavisia melusina	Ferguson et al. 1984	414
Dioryctria mutuurai	Neunzig 2003	278			

**Table 2.** Faunal additions to list as either introductions or recorded from southern states.

Species	State	Year	Origin	Reference	Note
Sarabotys cupreicostalis	Arizona	1974	Southern	This paper	1
Sclerocona acutella	Massachusetts	1984	Introduced	Wagner et al. 2003	2
Pseudopyrausta marginalis	Texas	1980	Southern	Bordelon and Knudson 2010	4
Aponia aponianalis	Texas	2010	Southern	Bordelon 2011	11
Sitochroa palealis	Illinois	2002	Introduced	Passoa et al. 2008	13
Paracorsia repandalis	Indiana	2010	Introduced	Vargo pers. comm.	14
Pyrausta cardinalis	Florida	2010	Southern	Hayden et al. 2011	19
Ecpyrrhorrhoe puralis	Georgia & Maryland	2000	Introduced	Solis et al. 2010	23
Microthyrix lelex	Florida	1995	Southern	Hayden and Dickel 2014	33
Pantographa suffusalis	Texas	2008	Southern	Bordelon 2009	34
Hileithia decostalis	Mississippi	1979	Southern	Mather and Munroe 1992	42
Lineodes triangulalis	Florida	1991	Southern	Hayden and Dickel 2014	45
Lineodes elcodes	California	2004	Southern	Epstein 2004	46
Lineodes multisignalis	Florida	2013	Southern	Covell and Hayden 2014	47
Lineodes vulnifica	Florida	1945	Southern	Annand 1945	48
Lamprosema baracoalis	Texas	1973	Southern	Blanchard and Knudson 1985	50
Neoleucinodes torvis	Florida	1984	Southern	Hayden and Dickel 2014	51
Nacoleia charesalis	Florida	2012	Introduced	Dixon and Anderson 2014a, Hayden and Troubridge 2015	57
Duponchelia fovealis	California	2004	Southern	Epstein 2004	58
Glyphodes onychinalis	California	2006	Southern	Solis 2008	63
Maruca vitrata	Florida	1980	Southern	Dickel 1981	64
Omiodes martyralis	Louisiana	1984	Southern	Brou 1985; Brou 1994	68
Palpita persimilis	Florida	1980	Southern	Hayden and Buss 2012	70
Nomophila triticalis	Florida	1992	Southern	Heppner 2011; Hayden and Dickel 2014	77
Samea druchachalis	Texas	1981	Southern	Bordelon and Knudson 2011	78
Cangetta micralis	Florida	1975	Southern	Hayden and Dickel 2014	84
Eurrhyparodes splendens	Texas	1975	Southern	Blanchard and Knudson 2007	85
Sisyracera contortilinealis	Florida	2012	Southern	Bugguide 2003–2015a, Hayden pers. comm.	92
Schacontia themis	Florida	1984	Southern	Goldstein et al. 2013	102
Schacontia rasa	Florida	2014	Southern	Goldstein et al. 2013	103
Ennomosia basalis	Florida	1992	Southern	Hayden and Dickel 2014	107
Neomusotima conspurcatalis	Florida	2008	Introduced	Boughton and Pemberton 2012	115
Thaumatopsis digrammellus	Arizona	1922	Southern	Landry 1995	136
Parapediasia ligonellus	Florida	1986	Southern	Landry 1995	150
Epitamyra albomaculalis	Florida	2014	Southern	Dixon and Anderson 2014b	190
Epimorius testaceellus	Florida	1974	Southern	Ferguson 1991	212
Stenopaschia trichopteris	Texas	1994	Southern	Knudson 1996	213
Anypsipyla univitella	Florida	1983	Southern	Dickel 1988	231
Fundella ignobilis	Texas	1982	Southern	Knudson 1983	238
Scorylus cubensis	Florida	1984	Southern	Dickel 1987	239
Stylopalpia luniferella	Florida	1985	Southern	Dickel 1988	268
Quasisarata subosseella	Florida	1981	Southern	Dickel 1987	291
Chorrera extrincica	Texas	1982	Southern	Knudson 1983	298
Arcola malloi	Florida	1971	Introduced	Brown and Spencer 1973	300
Ancylosis bonhoti	Florida	1980	Southern	Hayden and Dickel 2014	310
Cassiana malacella	Florida	No date	Southern	Neunzig 1997	313
Mescinia berosa	Florida	No date	Southern	Neunzig 1997	314
Mescinia parvula	Florida	No date	Southern	Neunzig 1997	315
Phestinia costella	Florida	2011	Southern	Solis 2011; Diaz et al. 2015	318
Comotia torsicornis	Florida	1992	Southern	Heppner and Dickel 2008	319
Cactoblastis cactorum	Florida	1989	Introduced	Habeck and Bennett 1990	340
Ozamia lucidalis	Florida	No date	Southern	Habeck and Bennett 1990	345
Australephestiodes stictella	Florida	No date	Southern	Neunzig 1990	353
Moodnodes plorella	Florida	No date	Southern	Neunzig 1990	355
Ribua innoxia	South Carolina	2002	Southern	This paper	367
Bethulia championella	Arizona	1961	Southern	Neunzig and Solis 2006	368
Bema neuricella	Florida	1984	Southern	Dickel 1987	377
Milgithea trilinearis	Florida	1993	Southern	Solis 1993	386
Tallula beroella	Arizona	No date	Southern	This paper	397
‘Macalla’ glastianalis	Texas	1970s	Southern	Knudson unpub.	399

**Figure 1.**
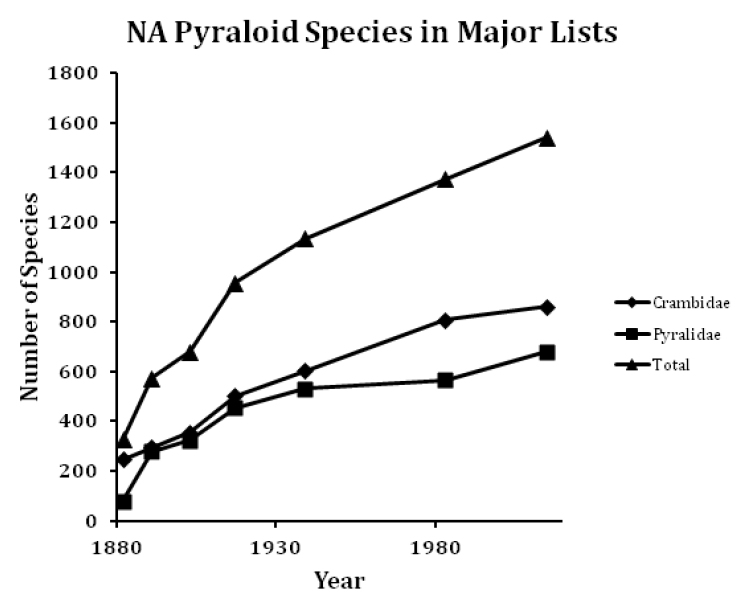
Total number of Pyraloidea species in North America north of Mexico in major published lists.

The MONA check list was the product of a number of sequential lists of the known Pyraloidea species in the United States and Canada beginning over 100 years ago (Grote 1882, Fernald 1891 in Smith 1891, Fernald 1903 and Hulst 1903 in Dyar 1903, Barnes and McDunnough 1917, McDunnough 1939, Munroe 1983 and Klots 1983 in Hodges et al. 1983). These lists progressively added more species, with the MONA check list more than doubling the known total from 1903 (Table 3; Figure 1). The MONA check list included 807 species in the Crambidae and 567 species in the Pyralidae. To the best of our knowledge the present list is complete up to August 2015. This check list includes 861 species in the Crambidae and 681 species in the Pyralidae from North America north of Mexico, 168 more than the 1983 list and a total of 1542 species, an increase of more than 10%.

**Table 3.** Number of Pyraloidea species reported in major North American lists.

Source list	Crambidae	Pyralidae	Total
Grote 1882	248	79	327
Fernald in Smith 1891	294	280	574
Fernald & Hulst in Dyar 1903 [1902]	356	323	679
Barnes and McDunnough 1917	501	455	956
McDunnough 1939	604	531	1135
Munroe & Klots in Hodges et al. 1983	807	567	1374
Present list	861	681	1542

A number of workers contributed to our increased knowledge about the identity and relationships among pyraloid species in America north of Mexico after the MONA check list was published. Eugene Munroe continued to publish, along with A. Blanchard, E. C. Knudson, B. Landry, H. H. Neunzig, J. Shaffer, and M. A. Solis, most actively describing species, revising groups and adding to the North American list of pyraloids. Within the last 30 or 40 years major revisions of subfamilies that included American species or genera included the Scopariinae (Munroe 1972a), Acentropinae (Munroe 1972a as Nymphulinae; Speidel 2005), Odontiinae (Munroe 1972b), Glaphyriinae (Munroe 1972b, 1973 including Evergestinae), Pyraustinae (Munroe 1976a,b), Crambinae (Landry 1995), Schoenobiinae (Martinez 2010) (but new names not yet published), Chrysauginae (Cashatt 1968, 1969; Solis et al. 2013), Epipaschiinae (Solis 1993), and most of the Phycitinae (Neunzig 1986, 1990, 1997, 2003; Shaffer 1968).

In addition, recent work in Europe, particularly that by Maes (1987, 1994, [2002], 2003, 2005), Leraut (1997, 2002, 2003a, 2003b, 2005a, 2005b, 2006a, 2006b, [2008], 2012, 2014) and Nuss (1999, 2005), and work by contributors to the Global Information System on Pyraloidea (Nuss et al. 2003–2015), has clarified placement of many North American taxa and synonymized several genera with European genera.

We have made significant higher-order taxonomic and/or nomenclatural changes from the MONA check list to reflect Pyraloidea systematic research in the last 40 years. Most prominently this includes the removal of Thyrididae and Hyblaeidae from the Pyraloidea (Minet 1985), and splitting the Pyralidae (*sensu lato*) into two families, the Crambidae and Pyralidae (*sensu stricto*) (Minet 1982); Munroe (1983) used the informal terms of Series Crambiformes and Series Pyraliformes, but followed Minet’s division of two families in Munroe (1995a). We also have adopted the subfamily arrangement of Regier et al. (2012) based on recent molecular evidence that supported the morphological evidence or hypothesized new relationships and/or synonymies. The Pyralidae subfamilies remain the Chrysauginae, Galleriinae, Pyralinae, Epipaschiinae, and Phycitinae as in Munroe (1983), with the exception of the Peoriinae, now considered part of the Phycitinae (Minet 1982) and relegated to tribal status within the Phycitinae by Solis and Mitter (1992). In the Crambidae, there are several major changes from the MONA check list. 1) The subfamily Evergestinae is synonymized with Glaphyriinae, as the oldest name, based on Regier et al. (2012). 2) The genera in the tribe Dichogamini (in the Odontiinae in MONA) are placed in the Glaphyriinae (Munroe and Solis 1999). 3) The genera in the tribe Ambiini (in the Nymphulinae in MONA) are placed in the Musotiminae (Speidel 1981). 4) The name Acentropinae is applied to the Nymphulinae (in MONA) as the oldest name for the group (Solis 1999; ICZN 2003). 5) The tribes Pyraustini and Spilomelini are elevated to subfamily level (Minet 1982). 6) The subfamily Cybalomiinae is deleted from the North American region, its lone species transferred to Glaphyriinae (Solis 2009). 7) The genera in the subfamily Ancylolomiinae (in MONA) are placed in the Crambinae (Landry 1995; Munroe 1995a). 8) The subfamily Lathrotelinae is recognized for the genus Sufetula (Minet 2015).

In this check list we give the complete synonymy as currently known for all species, and list known subspecies with synonyms, indicating where taxa are extralimital or introduced. We have reorganized many of the genera based on the NTCL (Munroe 1995a, b; Solis 1995; Solis, Becker and Munroe 1995; M. Shaffer and Solis 1995; J. Shaffer 1995) because it reflects a more recent concept of generic relationships. We have also followed Landry (1995) for the arrangement of genera in the Crambinae. We have not included the taxonomic rank of tribe because so many are not based on characters, or the tribal structure breaks down upon study of the tribe in other regions of the world. We also list described subgenera as synonyms because they are not universally applied to most of the list.

MAS has externally examined types or vouchers for much of the Western Hemisphere fauna, and we have confirmed vouchers for the recorded introductions. Researchers in other parts of the world have examined species, internally as well as externally, and, in some cases, synonymized some of the older generic names (e.g. Anania – Leraut 2005a; Tränker et al. 2009, Elophila – Yoshiyasu 1985; Speidel 2005, Hypsopygia – Leraut 2006a). In some cases when the genus was synonymized, new combinations of species were not published. We include those combinations in this list based on the synonymies indicated in those publications. Citations for all species described since the MONA check list are included in the References, but citations for older species descriptions are not included. Taxa with a superscript number, e.g. ^122^, have a corresponding note in the Notes section explaining or clarifying information about that name (e.g. new synonymies, recent introductions, and discoveries regarding Neotropical taxa that extend northward). Authors and years in parentheses, e.g. (Walker 1859), indicate that the species was originally described in another genus. For all names, the original genus is listed in parentheses after the complete name (even when the same as the current genus). The original spelling of the specific epithet is used to maintain consistency with recent lists by Lafontaine and Schmidt (2010) and Poole (1996). By listing the original spelling, appropriate combinations can be formed by future authors based on the gender of any current or future generic placement following ICZN article 34.2 (ICZN 1999). Brackets around the year of publication, e.g. Walker, [1866], indicate that the year of publication has been determined to be different from that in the publication. Our synonymy includes only names associated with descriptions or emendations; we have eliminated misapplied names and misspellings that have often been listed in previous check lists. In several cases, the attribution of authorship has been corrected because the author of the name was not the same as the author of the overall publication. The most prominent of these are Ragonot, E. L. & Hampson, G. F. 1901 [November 22] (Fletcher and Nye 1984) and authorship by Cramer and/or Stoll (1779–1782) (Fletcher and Nye 1982). Nuss et al. (2003–2015) also indicate that authorship of Felder (1875) should actually be Felder, C., Felder, R. & Rogenhofer, A.F. (1875) (Fletcher and Nye 1979).

**Figure 2.**
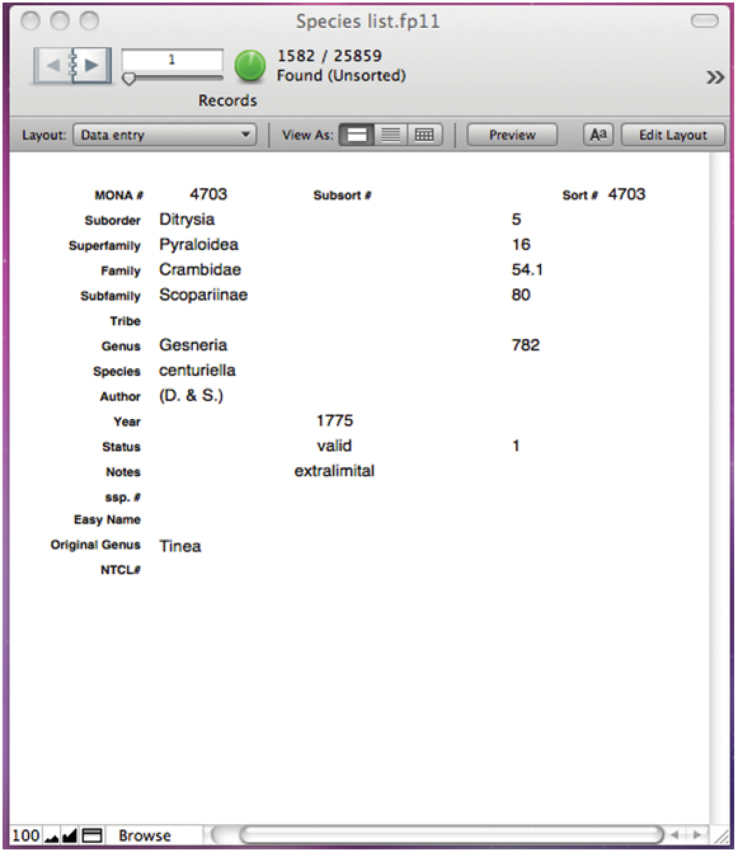
Data record illustrating numbering system to enable phylogenetic sorting without new species numbers.

We opted not to create a new numbering system for the list, but have included the original MONA and NTCL numbers (even when a name is in new synonymy) for cross-reference to those lists. We anticipate a new numbering system will be in place when the entire North American check list becomes available on-line. When lists are numbered, the computer sorting and ordering of taxa phylogenetically becomes onerous, particularly as species are reassigned to genera or subfamilies and new species are described. In our database, we have assigned sequential numbers to superfamilies, families, subfamilies, tribes and genera, in addition to species (currently with MONA numbers and additions interpolated with decimals). Figure 2 shows a data record illustrating this system. These number assignments are for internal database use only and allow for correct hierarchical sorting to higher-level taxa while still retaining the original species numbers, allowing phylogenetic sorting rather than only alphabetic. This logic could easily be extended to subgenera or species groups, particularly for very large genera. This avoids complete renumbering of species in the database, and allows easy reference to the previous list. We intend to keep this database (maintained by BGS) current, including updates in GlobIZ (Nuss et al. 2003–2015). The database can generate an unformatted version of this check list, or export into Excel format.

We and our colleagues who study Pyraloidea are aware of other undescribed species, new synonymies, and new combinations, but we have not included these in the list pending publication. Molecular data have also recently been applied to species level questions in the Pyraloidea, (e.g. Anania – Yang et al. 2012) and we expect additional discoveries and changes to nomenclature. Small groups such as the Pyralinae, Epipaschiinae, and Galleriinae are likely to have just a handful of additional species when revised at the species level. Most future additions are likely to be in the largest subfamilies, the Spilomelinae, Crambinae, and Phycitinae where some genera remain to be described, larger genera are known to be polyphyletic (see misplaced species at the end of genera in the NTCL), and smaller genera may be paraphyletic to other genera. Recent molecular work has largely supported previous morphological studies, but we expect generic concepts to experience significant changes in the future because most generic taxonomic groupings still require adequate investigation using morphological and molecular techniques.

We plan to continue updating this list, thus we welcome information on omissions, corrections, additions or updates.

## Check list

**MONA# NTCL#**

**Pyraloidea**

**Crambidae**

**Pyraustinae**

**Munroeodes** Amsel, 1957

Munroeia Amsel, 1956; preocc. by Marion, 1954

**thalesalis** (Walker, 1859); valid (Botys) 4934 1373

**Saucrobotys** Munroe, 1976

**fumoferalis** (Hulst, 1886); valid (Botis) 4935

**futilalis** (Lederer, 1863); valid (Botys) 4936 1374

erectalis (Grote, 1876); syn. (Botis) 4936 1374

**a. inconcinnalis** (Lederer, 1863); ssp. (Botys) 4936 1374

crocotalis (Grote, 1881); syn. (Botis) 4936 1374

festalis (Hulst, 1886); syn. (Botis) 4936 1374

**Sarabotys** Munroe, 1964

**cupreicostalis** (Dyar, 1913)^1^; valid (Phlyctaenodes) 1376

**Nascia** Curtis, 1835

**acutellus** (Walker, 1866); valid (Crambus) 4937

venalis (Grote, 1878); syn. (Botis) 4937

**Sclerocona** Meyrick, 1890

**acutellus** (Eversmann, 1842)^2^; valid introduced (Chilo)

cilialis (Herrich-Schäffer, 1849); syn. (Duponchelia)

tincticostellus (Walker, 1863); syn. (Crambus)

**a. sinensellus** (Walker, 1863)^3^; ssp. (Crambus)

**Epicorsia** Hübner, 1818

**oedipodalis** (Guenée, 1854); valid (Botys) 4938 1403

butyrosa (Butler, 1878); syn. (Botys) 4938 1403

**Pseudopyrausta** Amsel, 1956

**marginalis** (Dyar, 1914)^4^; valid (Nacoleia) 1426

**santatalis** (Barnes & McDunnough, 1914);

valid (Blepharomastix) 4939 1428

**Oenobotys** Munroe, 1976

**vinotinctalis** (Hampson, 1895); valid (Pionea) 4940 1433

**texanalis** Munroe & A. Blanchard, 1976; valid (Oenobotys) 4941 1434

**Triuncidia** Munroe, 1976

**eupalusalis** (Walker, 1859); valid (Botys) 4942 1436

**Crocidophora** Lederer, 1863

Crocidosema Lederer, 1863^5^

Monocrocis Warren, 1895^5^

**pustuliferalis** Lederer, 1863; valid (Crocidophora) 4943

**serratissimalis** Zeller, 1872; valid (Crocidophora) 4944

subdentalis (Grote, 1873); syn. (Botis) 4944

**tuberculalis** Lederer, 1863; valid (Crocidophora) 4945

**Ostrinia** Hübner, [1825]

Micractis Warren, 1892

Eupolemarcha Meyrick, 1937

Zeaphagus Agenjo, 1952

**penitalis** (Grote, 1876); valid (Botys) 4946 1438

nelumbialis (Smith, 1890); syn. (Pyrausta) 4946 1438

pyraustalis (Dyar, 1925); syn. (Tholeria) 4946 1438

**a. brasiliensis** Mutuura & Munroe, 1970^6^;

ssp. extralimital (Ostrinia) 1438

**b. rubrifusa** (Hampson, 1913); ssp. extralimital (Pyrausta) 1438

**obumbratalis** (Lederer, 1863); valid (Botys) 4947

obliteralis (Walker, [1866]); syn. (Botys) 4947

ainsliei (Heinrich, 1919); syn. (Pyrausta) 4947

**marginalis** (Walker, [1866]); valid (Scopula) 4948

stenopteralis (Grote, 1878); syn. (Botys) 4948

**nubilalis** (Hübner, 1796); valid (Pyralis) 4949

silacealis (Hübner, 1796); syn. (Pyralis) 4949

glabralis (Haworth, 1803)^5^; syn. (Pyralis)

appositalis (Lederer, 1858)^5^; syn. (Botys)

paulalis (Fuchs, 1900)^5^; syn. infrasubsp. (Botis)

fanalis (Costantini, 1923)^5^; syn. (Pyrausta)

rubescens (Krulikovsky, 1928)^5^; syn. (Pyrausta)

insignis (Skala, 1928)^5^; syn. infrasubsp. (Pyrausta)

margarita (Skala, 1928)^5^; syn. infrasubsp. (Pyrausta)

flava (Dufrane, 1930)^5^; syn. infrasubsp. (Pyrausta)

minor (Dufrane, 1930)^5^; syn. infrasubsp. (Pyrausta)

fuscalis (Romaniszyn, 1933)^5^; syn. infrasubsp. (Pyrausta)

**a. mauretanica** Mutuura & Munroe, 1970^5^;

ssp. extralimital (Ostrinia)

**b. persica** Mutuura & Munroe, 1970^5^;

ssp. extralimital (Ostrinia)

**Fumibotys** Munroe, 1976

**fumalis** (Guenée, 1854); valid (Ebulea) 4950

orasusalis (Walker, 1859); syn. (Scopula) 4950

badipennis (Grote, 1873); syn. (Botis) 4950

**Perispasta** Zeller, 1876

**caeculalis** Zeller, 1876; valid (Perispasta) 4951 1439

immixtalis Grote, 1881; syn. (Perispasta) 4951 1439

**Anania** Hübner, 1823^7^

Palpita Hübner, 1806^5^; rejected work, ICZN opinions 97 & 278

Phlyctaenia Hübner, 1825

Eurrhypara Hübner, 1825

Perinephela Hübner, 1825

Polyctaenia Hübner, 1826

Ennychia Treitschke, 1828

Ebulea Doubleday, 1849

Algedonia Lederer, 1863

Opsibotys Warren, 1890

Udonomeiga Mutuura, 1954

Trichovalva Amsel, 1956

Pronomis Munroe & Mutuura, 1968

Proteurrhypara Munroe & Mutuura, 1969

Tenerobotys Munroe & Mutuura, 1971

Mutuuraia Munroe, 1976^8^

Nealgedonia Munroe, 1976^8^

Ametasia M.O. Martin, 1986

Ethiobotys Maes, 1997

**hortulata** (Linnaeus, 1758); valid (Phalaena (Geometra)) 4952

urticata (Linnaeus, 1761); syn. (Phalaena (Geometra)) 4952

urticalis (Denis & Schiffermüller, 1775); syn. (Pyralis) 4952

flavicauda (Retzius, 1783)^5^; syn. (Phalaena)?

dissoluta (Skala, 1928)^5^; syn. infrasubsp. (Eurrhypara)

minor (Dufrane, 1957)^5^; syn. infrasubsp. (Eurrhypara)

crassipunctata (Dufrane, 1957)^5^; syn. (Eurrhypara)

**tertialis** (Guenée, 1854)^9^; valid (Ebulea) 4953 1440

syringicola (Packard, 1870); syn. (Botys) 4953 1440

**plectilis** (Grote & Robinson, 1867)^9^; valid (Botys) 4953 1440

**tennesseensis** Yang, 2012^10^; valid (Anania)

**quebecensis** (Munroe, 1954); valid (Phlyctaenia) 4954

**leuschneri** (Munroe, 1976); valid (Phlyctaenia) 4955 1441

**extricalis** (Guenée, 1854); valid (Botys) 4956

intricatalis (Lederer, 1863); syn. (Botys) 4956

oppilalis (Grote, 1880); syn. (Botis) 4956

**a. dionalis** (Walker, 1859); ssp. (Pionea) 4956

nisoeecalis (Walker, 1859); syn. (Spilodes) 4956

beddeci (Dyar, 1913); syn. (Pyrausta) 4956

**mysippusalis** (Walker, 1859); valid (Botys) 4957 1458

humilalis (Lederer, 1863); syn. (Botys) 4957 1458

**funebris** (Ström, 1768);

valid extralimital (Phalaena (Geometra)) 4958

octomaculata (Linnaeus, 1771);

syn. extralimital (Phalaena (Geometra)) 4958

funeraria (Müller, 1774)^5^; syn. extralimital (Geometra)

atralis (Fabricius, 1775); syn. extralimital (Phalaena) 4958

guttalis (Denis & Schiffermüller, 1775);

syn. extralimital (Pyralis) 4958

trigutta (Esper, 1791); syn. extralimital (Noctua) 4958

octomaculalis (Treitschke, 1829);

syn. emendation extralimital (Ennychia) 4958

assimilis (Butler, 1879)^5^; syn. extralimital (Ennychia)

astrifera (Butler, 1879)^5^; syn. extralimital (Ennychia)

reducta (Weber, 1945)^5^; syn. extralimital (Pionea)

**a. glomeralis** (Walker, 1859); ssp. (Ennychia) 4958

**b. sabaudialis** Leraut, 1996^5^; ssp. extralimital (Anania)

**labeculalis** (Hulst, 1886); valid (Botis) 4959 1466

**Aponia** Munroe, 1964

**aponianalis** (H. Druce, 1899)^11^; valid (Pionea) 1478

**Hahncappsia** Munroe, 1976

**fordi** (Capps, 1967); valid (Loxostege) 4960 1483

**alpinensis** (Capps, 1967); valid (Loxostege) 4961 1484

**marculenta** (Grote & Robinson, 1867); valid (Botys) 4962 1485

**neomarculenta** (Capps, 1967); valid (Loxostege) 4963

**pseudobliteralis** (Capps, 1967); valid (Loxostege) 4964 1486

**neobliteralis** (Capps, 1967); valid (Loxostege) 4965

**jaralis** (Schaus, 1920); valid (Phlyctaenodes) 4966 1487

**mancalis** (Lederer, 1863); valid (Botys) 4967 1488

**pergilvalis** (Hulst, 1886); valid (Botis) 4968 1489

**cochisensis** (Capps, 1967); valid (Loxostege) 4969 1490

**coloradensis** (Grote & Robinson, 1867); valid (Botys) 4970 1491

**ramsdenalis** (Schaus, 1920); valid (Phlyctaenodes) 4971 1492

**huachucalis** (Capps, 1967); valid (Loxostege) 4972 1493

**mellinialis** (Druce, 1899); valid (Epichronistis) 4973 1512

phrixalis (Dyar, 1914); syn. (Phlyctaenodes) 4973 1512

**Achyra** Guenée, 1849

Dosara Walker, 1859^12^

Eurycreon Lederer, 1863^5^

Tritaea Meyrick, 1884^5^

**bifidalis** (Fabricius, 1794); valid (Phalaena) 4974 1521

centralis (Fabricius, 1794)^5^; (Phalaena)

inornatalis (Walker, [1866]); syn. (Phlyctaenodes) 4974 1521

evanidalis (Berg, 1875); syn. (Eurycreon) 4974 1521

obsoletalis (Berg, 1875); syn. (Eurycreon) 4974 1521

orbitalis (Hampson, 1899);

syn. not Felder, Felder & Rogenhofer, 1875 (Phlyctaenodes) 1521

stolidalis (Schaus, 1940); syn. (Loxostege) 4974 1521

**rantalis** (Guenée, 1854); valid (Nymphula) 4975 1526

siriusalis (Walker, 1859); syn. (Botys) 4975 1526

licealis (Walker, 1859); syn. (Botys) 4975 1526

murcialis (Walker, 1859); syn. (Ebulea) 4975 1526

nestusalis (Walker, 1859); syn. (Scopula) 4975 1526

diotimealis (Walker, 1859); syn. (Scopula) 4975 1526

crinisalis (Walker, 1859); syn. (Scopula) 4975 1526

intractella (Walker, 1863); syn. (Nephopteryx) 4975 1526

crinitalis (Lederer, 1863);

syn. invalid emendation (Botys (Eurycreon)) 4975 1526

posticata (Grote & Robinson, 1867); syn. (Botys) 4975 1526

subfulvalis (Herrich-Schäffer, 1871); syn. (Botys) 4975 1526

communis (Grote, 1876); syn. (Botis) 4975 1526

collucidalis (Möschler, 1890); syn. (Eurycreon) 4975 1526

viscendalis (Möschler, 1890); syn. (Botys) 1526

caffreii (Flint & Malloch, 1920); syn. (Pyrausta) 4975 1526

**occidentalis** (Packard, 1873); valid (Scopula) 4976 1527

**Neohelvibotys** Munroe, 1976

**neohelvialis** (Capps, 1967); valid (Loxostege) 4977 1532

**arizonensis** (Capps, 1967); valid (Loxostege) 4978 1531

**polingi** (Capps, 1967); valid (Loxostege) 4979 1534

**Helvibotys** Munroe, 1976

**helvialis** (Walker, 1859); valid (Spilodes) 4980 1539

thycesalis (Walker, 1859); syn. (Botys) 4980 1539

apertalis (Walker, [1866]); syn. (Botys) 4980 1539

citrina (Grote & Robinson, 1867); syn. (Botys) 4980 1539

harpalis (Herrich-Schäffer, 1871); syn. (Botys) 1539

**pseudohelvialis** (Capps, 1967); valid (Loxostege) 4981 1540

**freemani** Munroe, 1976; valid (Helvibotys) 4982 1541

**pucilla** (Druce, 1895); valid (Filodes) 4984 1542

subcostalis (Dyar, 1912); syn. (Lygropia) 4983 1542

**Sitochroa** Hübner, [1825]

Spilodes Guenée, 1849

**aureolalis** (Hulst, 1886); valid (Eurycreon) 4985 1544

cyralis (Druce, 1895); syn. (Pyrausta) 4985 1544

**palealis** (Denis & Schiffermüller, 1775)^13^;

valid introduced (Pyralis)

flaveolata (Hufnagel, 1767); syn. (Phalaena)

stigmatalis (Villers, 1789); syn. (PhalaenaPyralis)

selenalis (Hübner, 1796); syn. (Pyralis)

algiralis (Allard, 1867); syn. (Spilodes)

**a. anaxisalis** (Walker, 1859); ssp. extralimital (Botys)

**b. extremalis** (Caradja, 1916); ssp. extralimital (Loxostege)

**dasconalis** (Walker, 1859); valid (Spilodes) 4986 1545

**chortalis** (Grote, 1873); valid (Eurycreon) 4987

**Arenochroa** Munroe, 1976

**flavalis** (Fernald, 1894); valid (Loxostege) 4988 1546

unipunctalis (Walter, 1928); syn. (Loxostege) 4988 1546

**Xanthostege** Munroe, 1976

**roseiterminalis** (Barnes & McDunnough, 1914);

valid (Loxostege) 4989 1547

**plana** (Grote, 1883); valid (Prothyma) 4990 1548

**Sericoplaga** Warren, 1892

**externalis** Warren, 1892; valid (Sericoplaga) 4991 1564

maclurae (Riley, 1893); syn. (Loxostege) 4991 1564

**Uresiphita** Hübner, [1825]

Uresiphoeta Agassiz, [1847]^5^; unjustified emendation

Mecyna Guenée, 1854; preocc. Doubleday, 1849

**reversalis** (Guenée, 1854); valid (Mecyna) 4992 1565

hilaralis (Herrich-Schäffer, 1871); syn. (Botys) 1565

**Paracorsia** Marion, 1959

**repandalis** (Denis & Schiffermüller, 1775)^14^;

valid introduced (Pyralis)

pallidalis (Hübner, 1796); syn. (Pyralis)

**Loxostege** Hübner, [1825]

Leimonia Hübner, [1825]^15^

Margaritia Stephens, 1827^5^

Boreophila Duponchel, [1845]

Cosmocrean Warren, 1892

Maroa Barnes & McDunnough, 1914

Polingia Barnes & McDunnough, 1914

Meridiophila Marion, 1963^16^

Parasitochroa Hannemann, 1964

**albiceralis** (Grote, 1878); valid (Botis) 4993 1566

**floridalis** Barnes & McDunnough, 1913; valid (Loxostege) 4994 1567

**lepidalis** (Hulst, 1886); valid (Prorasea) 4995 1568

**indentalis** (Grote, 1883); valid (Prorasea) 4996 1569

**kearfottalis** Walter, 1928; valid (Loxostege) 4997 1570

**terpnalis** Barnes & McDunnough, 1918; valid (Loxostege) 4998

**unicoloralis** (Barnes & McDunnough, 1914); valid (Maroa) 4999 1571

**allectalis** (Grote, 1877); valid (Botis) 5000 1572

perplexalis (Fernald, 1885); syn. (Eurycreon) 5000 1572

**typhonalis** Barnes & McDunnough, 1914; valid (Loxostege) 5001 1573

**oberthuralis** Fernald, 1894; valid (Loxostege) 5002 1574

**egregialis** Munroe, 1976; valid (Loxostege) 5003 1575

**sticticalis** (Linnaeus, 1761); valid (PhalaenaPyralis) 5004 1576

lupulina (Clerck, 1764); syn. preocc. by L., 1758 (Pyralis) 5004 1576

miana (O.F. Müller, 1764)^5^; syn. (PhalaenaTortrix)

fuscalis (Hübner, 1796);

syn. preocc. by D. & S., 1775 (Pyralis) 5004 1576

sylvata (Panzer, 1804)^5^; syn. (Pyralis)

tetragonalis (Haworth, 1811); syn. (Pyralis) 5004 1576

lupulinalis (Guenée, 1854); syn. emendation, part (Botys) 5004 1576

**a. tenebrosa** Caradja, 1939^5^; ssp. extralimital (Loxostege)

**mojavealis** Capps, 1967; valid (Loxostege) 5005

**kingi** Munroe, 1976; valid (Loxostege) 5006

**annaphilalis** (Grote, 1881); valid (Botis) 5007

**immerens** (Harvey, 1875); valid (Annaphila) 5008 1578

triumphalis Grote, 1902; syn. (Loxostege) 5008 1578

**quaestoralis** (Barnes & McDunnough, 1914); valid (Polingia) 5009 1579

**anartalis** (Grote, 1878); valid (Eurycreon) 5010

**a. lulualis** (Hulst, 1886); ssp. (Botis) 5010

**b. albertalis** Barnes & McDunnough, 1918;

ssp. (Loxostege) 5010

**c. saxicolalis** Barnes & McDunnough, 1918;

ssp. (Loxostege) 5010

**d. rainierensis** Munroe, 1976; ssp. (Loxostege) 5010

**ephippialis** (Zetterstedt, 1839); valid (Botys) 5011

dubitaria (Zetterstedt, 1839); syn. (Psodos) 5011

scandinavialis (Guenée, 1854); syn. (Boreophila) 5011

frigidalis (Guenée, 1854); syn. (Boreophila) 5011

**thallophilalis** (Hulst, 1886); valid (Botis) 5012

thrallophilalis Hulst, 1886;

incorrect original spelling (Loxostege) 5012

flavifimbrialis (Warren, 1892); syn. (Noctuelia) 5012

**brunneitincta** Munroe, 1976; valid (Loxostege) 5013

**offumalis** (Hulst, 1886); valid (Botis) 5014 1580

**sierralis** Munroe, 1976; valid (Loxostege) 5015

**a. internationalis** Munroe, 1976; ssp. (Loxostege) 5015

**b. tularealis** Munroe, 1976; ssp. (Loxostege) 5015

**c. sanpetealis** Munroe, 1976; ssp. (Loxostege) 5015

**commixtalis** (Walker, [1866]); valid (Scopula) 5016

indotatellus (Walker, 1866); syn. (Crambus) 5016

septentrionalis (Tengström, 1869); syn. (Botys) 5016

**cereralis** (Zeller, 1872); valid (Eurycreon (Spilodes)) 5017 1581

**Pyrausta** Schrank, 1802

Botys Latreille, 1802

Heliaca Hübner, 1806^5^; rejected by ICZN opinions 97 & 278

Haematia Hübner, 1818

Pyraustes Billberg, 1820; emendation

Botis Swainson, 1821; emendation

Heliaca Hübner, 1822

Tholeria Hübner, 1823^5^

Porphyritis Hübner, [1825]

Syllythria Hübner, [1825]

Panstegia Hübner, [1825]

Perilypa Hübner, [1825]

Ostreophena Sodoffsky, 1837;

repl. name for Botis Swainson, 1821

Rhodaria Duponchel, 1844^5^

Herbula Guenée, 1854

Synchromia Guenée, 1854

Cindaphia Lederer, 1863

Proteroeca Meyrick, 1884^17^

Sciorista Warren, 1890^5^

Autocosmia Warren, 1892

Aplographe Warren, 1892^5^

Anthocrypta Warren, 1892^5^

Hyaloscia Dognin, 1908

Trigonuncus Amsel, 1952

Mardinia Amsel, 1952^18^

Rattana Rose & Pajni, 1979^5^

**demantrialis** (Druce, 1895); valid (Blepharomastix) 5018 1591

atrisquamalis Dognin, 1905; syn. (Pyrausta) 1591

monotonigra Amsel, 1956; syn. (Pyrausta) 5018 1591

**nexalis** (Hulst, 1886); valid (Botis) 5019 1593

concinna (Warren, 1892); syn. (Autocosmia) 5019 1593

**sartoralis** Barnes & McDunnough, 1914; valid (Pyrausta) 5020 1594

**roseivestalis** Munroe, 1976; valid (Pyrausta) 5021 1595

**zonalis** Barnes & McDunnough, 1918; valid (Pyrausta) 5022 1596

**napaealis** (Hulst, 1886); valid (Paraedis) 5023 1597

**linealis** (Fernald, 1894); valid (Loxostege) 5024 1598

**ochreicostalis** Barnes & McDunnough, 1918; valid (Pyrausta) 5025 1599

**pilatealis** Barnes & McDunnough, 1914; valid (Pyrausta) 5026 1600

**lethalis** (Grote, 1881); valid (Botis) 5027 1601

**corinthalis** Barnes & McDunnough, 1914; valid (Pyrausta) 5028 1602

**volupialis** (Grote, 1877); valid (Botis) 5029 1603

**morenalis** (Dyar, 1908); valid (Metasia) 5030 1605

**atropurpuralis** (Grote, 1877); valid (Botis) 5031 1607

**nicalis** (Grote, 1878); valid (Stemmatophora) 5032

uxorculalis (Hulst, 1886); syn. (Botis) 5032

subnicalis (Warren, 1892); syn. (Syllythria) 5032

**grotei** Munroe, 1976; valid repl. name (Pyrausta) 5033 1609

augustalis (Grote, 1881); syn. preocc. by Felder,

Felder & Rogenhofer, 1875 (Botys) 5033 1609

**signatalis** (Walker, [1866]); valid (Rhodaria) 5034 1612

vinulenta (Grote & Robinson, 1867);

syn. repl. name (Botys) 5034 1612

medullalis (Snellen, 1899)^5^; syn. (Botys)

**pythialis** Barnes & McDunnough, 1918; valid (Pyrausta) 5035

**inveterascalis** Barnes & McDunnough, 1918; valid (Pyrausta) 5036

**inornatalis** (Fernald, 1885); valid (Botis) 5037

rosa (Druce, 1895); syn. (Syllythria) 5037

**shirleyae** Munroe, 1976; valid (Pyrausta) 5038

**coccinea** Warren, 1892; valid (Pyrausta) 5039

**bicoloralis** (Guenée, 1854); valid (Asopia) 5040

**augustalis** (Felder, Felder & Rogenhofer, 1875); valid (Botys) 5041 1621

**onythesalis** (Walker, 1859); valid (Botys) 5042 1627

**pseudonythesalis** Munroe, 1976; valid (Pyrausta) 5043 1628

**insignitalis** (Guenée, 1854); valid (Rhodaria) 5044 1629

eratalis (Walker, 1859); syn. (Botys) 1629

largalis (Walker, 1859); syn. (Asopia) 1629

ordinatalis (Walker, [1866]); syn. (Scopula) 1629

**aurea** (Hampson, 1913); valid (Pachyzancla) 5045 1632

**flavibrunnea** Hampson, 1913; valid (Pyrausta) 5046 1639

**klotsi** Munroe, 1976; valid (Pyrausta) 5047 1641

**flavofascialis** (Grote, 1882); valid (Botis) 5048 1643

**cardinalis** (Guenée, 1854)^19^; valid (Synchromia) 1646

**phoenicealis** (Hübner) 1818; valid (Haematia) 5049 1649

flegialis (Walker, 1859); syn. (Rhodaria) 5049 1649

noraxalis (Walker, 1859); syn. (Rhodaria) 5049 1649

**panopealis** (Walker, 1859); valid (Rhodaria) 5050 1650

coecilialis (Walker, 1859); syn. (Botys) 5050 1650

probalis (Walker, 1859); syn. (Rhodaria) 5050 1650

ocellusalis (Walker, 1859); syn. (Rhodaria) 5050 1650

catenalis (Walker, [1866]); syn. (Rhodaria) 5050 1650

juncturalis (Walker, [1866]); syn. (Rhodaria) 5050 1650

concatenalis (Walker, [1866]); syn. (Rhodaria) 5050 1650

heliamma (Meyrick, 1885); syn. (Myriostephes) 5050 1650

**rubricalis** (Hübner, 1796); valid (Pyralis) 5051

nescalis (Walker, 1859); syn. (Rhodaria) 5051

similalis (Lederer, 1863); syn. (Botys) 5051

**californicalis** (Packard, 1873); valid (Botys) 5052 1652

**a. sierranalis** Munroe, 1976; ssp. (Pyrausta) 5052

**pseuderosnealis** Munroe, 1976; valid (Pyrausta) 5053 1655

**dapalis** (Grote, 1881); valid (Botis) 5054 1656

**homonymalis** Munroe, 1976; valid repl. name (Pyrausta) 5055

submarginalis (Walker, [1866]);

syn. preocc. by Walker, [1866] (Herbula?) 5055

**generosa** (Grote & Robinson, 1867); valid (Botys) 5056 1657

**subgenerosa** Munroe, 1976; valid (Pyrausta) 5057

**orphisalis** Walker, 1859; valid (Pyrausta) 5058 1658

ochosalis Dyar, 1903; syn. (Pyrausta) 5058 1658

**tuolumnalis** Barnes & McDunnough, 1918; valid (Pyrausta) 5059

**insequalis** (Guenée, 1854)^20^; valid (Herbula) 5060 1659

subsequalis (Guenée, 1854); syn. (Herbula) 5060 1659

madetesalis (Walker, 1859); syn. (Isopteryx) 5060 1659

repletalis (Walker, [1866]); syn. (Herbula) 5060 1659

efficitalis (Walker, [1866]); syn. (Herbula?) 5060 1659

graminalis (Herrich-Schäffer, 1871); syn. (Botys) 5060 1659

matronalis (Grote, 1875); syn. (Botis) 5060 1659

borealis Druce, 1895;

syn. part, not Packard, 1867 (Pyrausta) 1659

**a. plagalis** Haimbach, 1908; ssp. (Pyrausta) 5060 1659

**b. fascetalis** (Berg, 1874); ssp. extralimital (Botis) 1659

cespitalis Berg, 1875;

syn. not D. & S., 1875 extralimital (Pyrausta) 1659

**c. petaluma** Munroe, 1976; ssp. (Pyrausta) 5060

**borealis** Packard, 1867^21^; valid (Pyrausta) 5060

**tatalis** (Grote, 1877); valid (Botis) 5061 1662

**retidiscalis** Munroe, 1976; valid (Pyrausta) 5062 1666

**andrei** Munroe, 1976; valid (Pyrausta) 5063 1667

**perrubralis** (Packard, 1873); valid (Botys) 5064 1676

**a. saanichalis** Munroe, 1951; ssp. (Pyrausta) 5064

**b. shastanalis** Munroe, 1976; ssp. (Pyrausta) 5064 1676

**scurralis** (Hulst, 1886); valid (Botis) 5065 1677

postrubralis Hampson, 1899; syn. (Pyrausta) 5065 1677

**a. awemealis** Munroe, 1976; ssp. (Pyrausta) 5065

**arizonicalis** Munroe, 1976; valid (Pyrausta) 5066

arizonensis Munroe, 1976;

syn. preocc. by Munroe, 1957 (Pyrausta) 5066

**semirubralis** (Packard, 1873); valid (Botys) 5067 1679

**unifascialis** (Packard, 1873); valid (Botys) 5068 1682

obnigralis (Hulst, 1886); syn. (Botis) 5068 1682

**a. subolivalis** (Packard, 1873); ssp. (Botys) 5068

hircinalis (Grote, 1875); syn. (Botis) 5068

**b. rindgei** Munroe, 1957; ssp. (Pyrausta) 5068 1682

**c. arizonensis** Munroe, 1957; ssp. (Pyrausta) 5068 1682

**tyralis** (Guenée, 1854); valid (Rhodaria) 5069 1686

erosnealis Walker, 1859; syn. variety (Pyrausta) 5069 1686

agathalis (Walker, 1859); syn. (Rhodaria) 5069 1686

diffissa (Grote & Robinson, 1867); syn. (Botys) 5069 1686

bellulalis (Hulst, 1886); syn. (Botis) 5069 1686

idessa (Druce, 1895); syn. (Syllythria) 5069 1686

**laticlavia** (Grote & Robinson, 1867); valid (Botys) 5070 1687

cinerosa (Grote & Robinson, 1867); syn. form (Botys) 5070 1687

**acrionalis** (Walker, 1859); valid (Rhodaria) 5071 1688

acuphisalis (Walker, 1859); syn. (Rhodaria) 5071 1688

proceralis (Lederer, 1863); syn. (Botys) 5071 1688

rubicundalis (Lederer, 1863);

unavailable name published in synonomy (Botys) 5071 1688

sumptuosalis Walker, [1866]; syn. (Pyrausta) 5071 1688

haruspica (Grote & Robinson, 1867); syn. (Botys) 5071 1688

rufifimbrialis (Grote, 1881); syn. (Botis) 5071 1688

**obtusanalis** Druce, 1899; valid (Pyrausta) 5072 1689

**niveicilialis** (Grote, 1875); valid (Botis) 5073

**fodinalis** (Lederer, 1863); valid (Botys) 5074

**a. monticola** Munroe, 1976; ssp. (Pyrausta) 5074

**b. septentrionicola** Munroe, 1976; ssp. (Pyrausta) 5074

**socialis** (Grote, 1877); valid (Botis) 5075

**a. perpallidalis** Munroe, 1976; ssp. (Pyrausta) 5075

**antisocialis** Munroe, 1976; valid (Pyrausta) 5076 1690

**Hyalorista** Warren, 1892

Pyraustopsis Amsel, 1956

**taeniolalis** (Guenée, 1854); valid (Rhodaria) 5077 1713

directalis (Walker, [1866]); syn. (Rhodaria) 1713

**Portentomorpha** Amsel, 1956

Apoecetes Munroe, 1956

**xanthialis** (Guenée, 1854); valid (Botys) 5078 1717

superbalis (Walker, [1866]); syn. (Botys) 5078 1717

incalis (Snellen, 1875); syn. (Botys) 5078 1717

rosealis (Möschler, 1890); syn. (Botys) 5078 1717

**Daulia** Walker, 1859^22^

Girtexta Swinhoe, 1890

**magdalena** (Fernald, 1892); valid (Botys) 5295 2993

**arizonensis** Munroe, 1957; valid (Daulia) 5296 2994

**Ecpyrrhorrhoe** Hübner, [1825]

Harpadispar Agenjo, 1952

Pyraustegia Marion, 1963

Yezobotys Munroe & Mutuura, 1969

**puralis** (South, 1901)^23^; valid introduced (Pionea)

**Spilomelinae**^24^

**Phaedropsis** Warren, 1890

Trichognathos Amsel, 1956

**chromalis** (Guenée, 1854); valid (Asopia) 5246 1724

**principialis** (Lederer, 1863)^25^; valid (Botys) 5246 1725

**stictigramma** (Hampson, 1912); valid (Sylepta) 5247 1730

**Diastictis** Hübner, 1818

Anomostictis Warren, 1892

**argyralis** Hübner, 1818; valid (Diastictis) 5253 1743

**pseudargyralis** Munroe, 1956; valid (Diastictis) 5254 1744

**ventralis** (Grote & Robinson, 1867); valid (Botis) 5255 1745

**a. seamansi** Munroe, 1956; ssp. (Diastictis) 5255

**fracturalis** (Zeller, 1872); valid (Botis) 5256 1746

**holguinalis** Munroe, 1956; valid (Diastictis) 5257 1747

**viridescens** Munroe, 1956; valid (Diastictis) 5258 1749

**robustior** Munroe, 1956; valid (Diastictis) 5259 1750

**sperryorum** Munroe, 1956; valid (Diastictis) 5260 1751

**caecalis** (Warren, 1892); valid (Anomostictis) 5261

**Framinghamia** Strand, 1920^26^

**helvalis** (Walker, 1859); valid (Pionea) 5262

oscitalis (Grote, 1880); syn. (Botis) 5262

gyralis (Hulst, 1886); syn. (Botis) 5262

botys Strand, 1920; syn. (Framinghamia) 5262

**Lygropia** Lederer, 1863

Hyperthalia Warren, 1896^27^

**fusalis** Hampson, 1904^28^, ^29^, ^30^; valid (Lygropia) 1775

hampsoni (Barnes & McDunnough, 1913); syn. (Nacoleia) 1775

**tripunctata** (Fabricius, 1794); valid (Phalaena) 5248 1758

campalis (Guenée, 1854); syn. (Botys) 5248 1758

cubanalis (Guenée, 1854); syn. (Botys) 5248 1758

**memmialis** (Walker, 1859)^31^; valid (Botys) 5248 1759

**plumbicostalis** (Grote, 1871); valid (Botys) 5249 1760

**rivulalis** Hampson, 1899^29^; valid (Lygropia) 5250

nymphulalis (Haimbach, 1908); syn. (Blepharomastix) 5250

**octonalis** (Zeller, 1873)^29^, ^30^; valid (Orobena) 5251 1781

sexmaculalis (Grote, 1876); syn. (Botis) 5251 1781

**Lypotigris** Hübner, [1825]

**reginalis** (Stoll, 1781); valid (Phalaena) 5252 1784

**Microthyris** Lederer, 1863

Crossophora Möschler, 1890; preocc. by Meyrick, 1883

Cyclocena Möschler, 1890^32^

**anormalis** (Guenée, 1854); valid (Botys) 5263 1787

helcitalis (Walker, 1859); syn. (Botys) 5263 1787

orphnealis (Walker, 1859); syn. (Botys) 5263 1787

dracusalis (Walker, 1859); syn. (Botys) 5263 1787

subaequalis (Walker, [1866]); syn. (Botys) 5263 1787

**prolongalis** (Guenée, 1854); valid (Botys) 5264 1790

sectalis (Guenée, 1854); syn. (Botys) 5264 1790

eurytalis (Walker, 1859); syn. (Botys) 5264 1790

agenoralis (Walker, 1859); syn. (Botys) 5264 1790

**lelex** (Cramer, 1777)^33^; valid (Phalaena) 2991

flexalis (Möschler, 1881); syn. (Botis) 2991

janiralis (Möschler, 1886); syn. (Botys) 2991

gestatalis (Möschler, 1890); syn. (Cyclocena) 2991

foviferalis (Hampson, 1895); syn. (Haritala) 2991

**Pantographa** Lederer, 1863

**limata** Grote & Robinson, 1867; valid (Pantographa) 5241 1797

**suffusalis** Druce, 1895^34^; valid (Pantographa) 1799

**Phostria** Hübner, [1819]

Vatica Walker, 1869^5^

Plectroctena Snellen, 1881^5^

Condega Moore, 1886^5^

Saroscelis Meyrick, 1894^5^

Plectrona Snellen, 1895^5^

Hoplisa Snellen, 1899^5^

Oedematarcha Swinhoe, 1900^5^

Parbokla Swinhoe, 1900^5^

Antennodes Swinhoe, 1906^5^

**tedea** (Stoll, 1780); valid (PhalaenaPyralis) 5265 1805

**oajacalis** (Walker, [1866]); valid (Botys?) 5266 1806

pelialis (Felder, Felder & Rogenhofer, 1875); syn. (Botys) 5266 1806

**Pleuroptya** Meyrick, 1890

Loxoscia Warren, 1890

**silicalis** (Guenée, 1854); valid (Botys) 5243 1840

cypraealis (Walker, 1859); syn. (Botys) 5243 1840

sublutalis (Druce, 1895)^5^; syn. (Condylorrhiza)

sublutalis (Warren, 1889); syn. (Hapalia) 5243 1840

penumbralis (Grote, 1877)^35^; syn. (Sylepta) 5242 1840

fluctuosalis (Dyar, [1903]); syn. (Sylepta) 1840

**Syllepte** Hübner, 1823

Sylepta Hübner, [1825]^5^

Epherema Snellen, 1892^5^

Arthriobasis Warren, 1896^5^

Polycorys Warren, 1896^5^

Haliotigris Warren, 1896^5^

Nothosalbia Swinhoe, 1900^5^

Haitufa Swinhoe, 1900^5^

Neomabra Dognin, 1905

Troctoceras Dognin, 1905^5^

Subhedylepta Strand, 1918^5^

**diacymalis** Hampson, 1912^29^, ^30^; valid (Syllepte) 5245 1860

**Cryptobotys** Munroe, 1956

**zoilusalis** (Walker, 1859); valid (Botys) 5282 1879

hilaralis (Möschler, 1886); syn. (Botys) 1879

micromphalis (Hampson, 1912); syn. (Syngamia) 1879

masculinalis (Barnes & McDunnough, 1913);

syn. (Sylepta) 5282 1879

**Herpetogramma** Lederer, 1863

Pachyzancla Meyrick, 1884

Acharana Moore, [1885]

Stenomeles Warren, 1892

Piloptila Swinhoe, 1894^5^

Pantoeocome Warren, 1896

Ptiloptila Hampson, 1899^5^

Macrobotys Munroe, 1950

Coremataria Amsel, 1956

Culcitaria Amsel, 1957; nomen nudum

**bipunctalis** (Fabricius, 1794); valid (Phalaena) 5272 1883

detritalis (Guenée, 1854); syn. (Botys) 5272 1883

lycialis (Walker, 1859); syn. (Botys) 5272 1883

philealis (Walker, 1859); syn. (Botys) 5272 1883

terricolalis (Möschler, 1881); syn. (Botys) 1883

repetitalis (Grote, 1882); syn. (Botys) 5272 1883

simplex Warren, 1892^5^; syn. (Herpetogramma)?

**fluctuosalis** (Lederer, 1863)^36^; valid (Botys) 5244 1881

ipomoealis (Capps, 1964)^36^; syn. (Pachyzancla) 5273 1881

**phaeopteralis** (Guenée, 1854); valid (Botys) 5274 1896

vecordalis (Guenée, 1854); syn. (Botys) 5274 1896

vestalis (Walker, 1859); syn. (Botys) 5274 1896

neloalis (Walker, 1859)^5^; syn. (Botys)

otreusalis (Walker, 1859)^37^; syn. (Botys)

triarialis (Walker, 1859)^5^; syn. (Botys)

additalis (Walker, 1862); syn. (Botys) 5274 1896

plebejalis (Lederer, 1863); syn. (Botys) 5274 1896

cellatalis (Walker, [1866]); syn. (Botys) 5274 1896

inhonestalis (Walker, [1866])^5^; syn. (Botys)

tridentalis (Snellen, 1872)^5^; syn. (Botys)

communalis (Snellen, 1875); syn. (Botys) 1896

intricatalis (Möschler, 1890);

syn. preocc. by (Lederer, 1863) (Botys) 1896

descripta (Warren, 1892)^38^; syn. (Acharana)

**pertextalis** (Lederer, 1863); valid (Botys) 5275

thesealis (Zeller, 1872);

syn. part, not Lederer, 1863 (Botis) 5275

gentilis (Grote, 1873); syn. (Botis) 5275

**abdominalis** (Zeller, 1872); valid (Botis) 5276

fissalis (Grote, 1882); syn. (Botis) 5276

**thestealis** (Walker, 1859); valid (Botys) 5277

magistralis (Grote, 1873); syn. (Botis) 5277

gulosalis (Hulst, 1886); syn. (Botis) 5277

**centrostrigalis** (Stephens, 1834)^39^; valid (Margaritia) 5278

**theseusalis** (Walker, 1859); valid (Botys) 5279

feudalis (Grote, 1875); syn. (Botis) 5279

**aeglealis** (Walker, 1859); valid (Botys) 5280

quinquelinealis (Grote, 1875); syn. (Botis) 5280

**sphingealis** Handfield & Handfield, 2011^40^;

valid (Herpetogramma)

**Hileithia** Snellen, 1875

**magualis** (Guenée, 1854)^41^; valid (Isopteryx) 5187 1937

medealis (Walker, 1859); syn. (Samea) 5187 1937

belusalis (Walker, 1859); syn. (Botys) 5187 1937

curtalis (Walker, [1866]); syn. (Asopia) 5187 1937

**decostalis** (Guenée, 1854)^42^; valid (Isopteryx) 1919

melicertalis (Walker, 1859); syn. (Samea) 1919

perseusalis (Walker, 1859); syn. (Zebronia) 1919

appialis Snellen, 1875; syn. (Hileithia) 1919

**aplicalis** (Guenée, 1854)^41^; valid (Isopteryx) 5188 1936

xeniolalis (Hulst, 1886); syn. (Isopteryx) 5188 1936

**rehamalis** (Dyar, 1914)^41^; valid (Bocchoris) 5189 1932

**differentialis** (Dyar, 1914)^41^; valid (Bocchoris) 5190 1926

**Pilocrocis** Lederer, 1863

Anisoctena Meyrick, 1894^5^

**ramentalis** Lederer, 1863; valid (Pilocrocis) 5281 1940

perfuscalis (Hulst, 1886); syn. (Zinckenia) 5281 1940

**Choristostigma** Warren, 1892

Namangania Amsel, 1952

**plumbosignalis** (Fernald, 1888); valid (Botis) 5128

**zephyralis** (Barnes & McDunnough, 1914); valid (Diasemia) 5129 1972

**roseopennalis** (Hulst, 1886); valid (Botis) 5130 1973

**perpulchralis** (Hampson, 1899); valid (Ischnurges) 5131 1974

chromophila (Dyar, 1914); syn. (Ischnurges) 5131 1974

microchroia (Dyar, 1916); syn. (Ischnurges) 5131 1974

**elegantalis** Warren, 1892; valid (Choristostigma) 5132 1975

argalis (Fernald, 1894); syn. (Metasia) 5132 1975

**disputalis** (Barnes & McDunnough, 1917); valid (Diasemia) 5133 1976

**leucosalis** (Barnes & McDunnough, 1914); valid (Diasemia) 5134 1978

**Geshna** Dyar, 1906

**cannalis** (Quaintance, 1898); valid (Hydrocampa) 5126 1980

**Hydriris** Meyrick, 1885

Spanista Lederer, 1863; preocc. by Foerster, 1862

Antiercta Amsel, 1956; repl. name

**ornatalis** (Duponchel, 1832); valid (Asopia) 5127 1982

saturnalis (Treitschke, 1835); syn. (Nymphula) 5127 1982

deciusalis (Walker, 1859); syn. (Pyralis) 5127 1982

invenustalis (Walker, [1866]); syn. (Botys) 5127 1982

fraterna (Butler, 1875); syn. (Cataclysta) 5127 1982

pulchellalis (Mabille, 1879)^43^; syn. (Stenia)

bifascialis (Heeger, 1938); syn. (Nymphula) 5127 1982

orientalis (Yamanaka, 1972)^44^; syn. (Ercta)

**Lineodes** Guenée, 1854

Scoptonoma Zeller, 1873

Ciraphorus Dyar, 1910

**fontella** Walsingham, 1913; valid (Lineodes) 5106 1993

**integra** (Zeller, 1873); valid (Scoptonoma) 5107 1999

**interrupta** (Zeller, 1873); valid (Scoptonoma) 5108

**triangulalis** Möschler, 1890^45^; valid (Lineodes) 5109 1989

cyclophora Hampson, 1913; syn. (Lineodes) 1989

**elcodes** (Dyar, 1910)^46^; valid (Ciraphorus) 1991

**multisignalis** Herrich-Schäffer, 1868^47^; valid (Lineodes) 2006

**vulnifica** Dyar, 1913^48^; valid (Lineodes) 2016

**Atomopteryx** Walsingham, 1891

Stenoptycha Zeller, 1863; preocc. by Agassiz, 1862

Zellerina Torre y Callejas, 1958^49^; repl. name

**solanalis** (Barnes & McDunnough, 1913);

valid (Stenoptycha) 5110 2022

**Lamprosema** Hübner, 1823

Orocala Walker, [1866]^5^

**lunulalis** Hübner, 1823; valid (Lamprosema) 5103 2029

**victoriae** Dyar, 1923; valid (Lamprosema) 5104 2032

**sinaloanensis** Dyar, 1923; valid (Lamprosema) 5105 2033

**baracoalis** Schaus, 1920^50^; valid (Lamprosema) 2045

**Neoleucinodes** Capps, 1948

**prophetica** (Dyar, 1914); valid (Leucinodes) 5102 2060

minimalis (Amsel, 1956); syn. (Leucinodes) 2060

**torvis** Capps, 1948^51^; valid (Neoleucinodes) 2061

pusilla (Amsel, 1956); syn. (Leucinodes) 2061

**Udea** Guenée, [1845]

Stantira Walker, 1863

Melanomecyna Butler, 1883

Mnesictena Meyrick, 1884

Protocolletis Meyrick, 1888

Protaulacistis Meyrick, 1899

Notophytis Meyrick, 1932

**rubigalis** (Guenée, 1854); valid (Scopula) 5079 2122

oblunalis (Lederer, 1863); syn. (Botys) 5079 2122

harveyana (Grote, 1877); syn. (Botis) 5079 2122

**profundalis** (Packard, 1873); valid (Botis) 5080 2123

**washingtonalis** (Grote, 1882); valid (Botis) 5081

invinctalis (Hulst, 1886); syn. (Botis) 5081

**a. hollandi** Munroe, 1966; ssp. (Udea) 5081

**b. nomensis** Munroe, 1966; ssp. (Udea) 5081

**c. **pribilofensis**** Munroe, 1966; ssp. (Udea) 5081

**octosignalis** (Hulst, 1886); valid (Botis) 5082 2124

straminea (Warren, 1892); syn. (Ebulea) 5082 2124

**vacunalis** (Grote, 1881); valid (Botis) 5083 2125

galactalis (Dyar, 1925); syn. (Pyrausta) 5083 2125

**torvalis** (Möschler, 1864); valid (Botys) 5084

gelida (M’Lachlan, 1878); syn. (Scoparia) 5084

**alaskalis** (Gibson, 1920); valid (Diasemia) 5085

**inquinatalis** (Lienig & Zeller, 1846); valid (Scopula) 5086

glacialis (Packard, 1867); syn. (Botys) 5086

albinalis (Della Beffa, 1942)^5^; syn. (Pionea)

**rusticalis** (Barnes & McDunnough, 1914);

valid (Phlyctaenia) 5087

**nordeggensis** (McDunnough, 1929); valid (Phlyctaenia) 5088

**berberalis** (Barnes & McDunnough, 1918);

valid (Phlyctaenia) 5089 2127

**indistinctalis** Warren, 1892; valid (Udea) 5090

**a. johnstoni** Munroe, 1966; ssp. (Udea) 5090

**sheppardi** (McDunnough, 1929); valid (Phlyctaenia) 5091

**saxifragae** (McDunnough, 1935); valid (Phlyctaenia) 5092

**brevipalpis** Munroe, 1966; valid (Udea) 5093

**cacuminicola** Munroe, 1966; valid (Udea) 5094

**beringialis** Munroe, 1966; valid (Udea) 5095

**derasa** Munroe, 1966; valid (Udea) 5096

**livida** Munroe, 1966; valid (Udea) 5097

**turmalis** (Grote, 1881); valid (Botis) 5098 2128

**a. catronalis** Munroe, 1966; ssp. (Udea) 5098

**b. tularensis** Munroe, 1966; ssp. (Udea) 5098

**c. griseor** Munroe, 1966; ssp. (Udea) 5098

**itysalis** (Walker, 1859); valid (Scoparia) 5099

variegata (Walker, 1863); syn. (Stantira) 5099

hyperborealis (Möschler, 1874); syn. (Botys) 5099

similissima (Caradja, 1916)^5^; syn. form (Pionea)

**a. mertensialis** Munroe, 1966; ssp. (Udea) 5099

**b. tillialis** (Dyar, 1904); ssp. (Phlyctaenia) 5099

**c. rindgeorum** Munroe, 1966; ssp. (Udea) 5099

**d. kodiakensis** Munroe, 1966; ssp. (Udea) 5099

**e. albimontanalis** Munroe, 1966; ssp. (Udea) 5099

**f. durango** Munroe, 1966; ssp. (Udea) 5099

**g. wasatchensis** Munroe, 1966; ssp. (Udea) 5099

**h. clarkensis** Munroe, 1966; ssp. (Udea) 5099

**i. marinensis** Munroe, 1966; ssp. (Udea) 5099

**abstrusa** Munroe, 1966; valid (Udea) 5100

**a. subarctica** Munroe, 1966; ssp. (Udea) 5100

**b. cordilleralis** Munroe, 1966; ssp. (Udea) 5100

**c. pullmanensis** Munroe, 1966; ssp. (Udea) 5100

**radiosalis** (Möschler, 1883); valid (Botys) 5101

**aenigmatica** (Heinrich, 1931)^52^; valid (Platytes)

**Anageshna** Munroe, 1956

**primordialis** (Dyar, 1906); valid (Geshna) 5176 2134

**a. vividior** Munroe, 1956; ssp. (Anageshna) 5176

**b. pallidior** Munroe, 1956; ssp. (Anageshna) 5176

**Apogeshna** Munroe, 1956

Euvalva Amsel, 1956

**stenialis** (Guenée, 1854); valid (Isopteryx) 5177 2136

australis (Hulst, 1886); syn. (Hydrocampa) 2136

**Blepharomastix** Lederer, 1863

Sozoa Walker, [1866]

Ichthyoptila Meyrick, 1936

**ranalis** (Guenée, 1854); valid (Stenia) 5182 2138

archasialis (Walker, 1859); syn. (Asopia) 5182 2138

ofellusalis (Walker, 1859); syn. (Botys) 5182 2138

olliusalis (Walker, 1859); syn. (Botys) 5182 2138

strictalis (Walker, [1866]); syn. (Botys) 5182 2138

gracilis (Grote & Robinson, 1867); syn. (Botys) 5182 2138

datisalis Druce, 1895^5^; syn. (Blepharomastix) 5182 2138

occidentalis Haimbach, 1908; syn. (Blepharomastix) 5182 2138

**pseudoranalis** (Barnes & McDunnough, 1914);

valid (Pyrausta) 5183 2139

**potentalis** (Barnes & McDunnough, 1914)^29^;

valid (Pyrausta) 5184

**achroalis** (Hampson, 1913)^29^, ^30^; valid (Pyrausta) 5185 2217

**haedulalis** (Hulst, 1886)^29^, ^30^; valid (Botis) 5186 2218

astigmalis (Hampson, 1899); syn. (Nomophila) 2218

irregulalis (Dyar, 1913); syn. (Nomophila) 2218

**schistisemalis** (Hampson, 1912)^29^, ^30^; valid (Nacoleia) 5191 2215

**Desmia** Westwood, 1832

Aediodes Guenée, 1854

Hyalitis Guenée, 1854

Arna Walker, 1856; preocc. by Walker, 1855

**funeralis** (Hübner, 1796); valid (Pyralis) 5159 2245

**maculalis** Westwood, 1832; valid (Desmia) 5160 2246

**subdivisalis** Grote, 1871; valid (Desmia) 5161 2247

nominabilis E. Hering, 1906; syn. (Desmia) 5161 2247

**ufeus** (Cramer, 1777); valid (PhalaenaNoctua) 5162 2298

orbalis (Guenée, 1854); syn. (Aediodes) 5162 2298

ufealis (Guenée, 1854)^5^; syn. emendation? (Hyalitis)

prognealis Walker, 1859; syn. (Desmia) 5162 2298

bulisalis Walker, 1859; syn. (Desmia) 5162 2298

divisalis Walker, [1866]^53^; syn. (Desmia?) 5163 2298

viduatalis Möschler, 1890; syn. (Desmia) 2298

**tages** (Cramer, 1777); valid (PhalaenaNoctua) 5164 2293

tagesalis (Guenée, 1854); syn. emendation (Hyalitis) 5164 2293

sertorialis Herrich-Schäffer, 1871; syn. (Desmia) 2293

**stenizonalis** Hampson, 1912; valid (Desmia) 5165 2290

**deploralis** Hampson, 1912; valid (Desmia) 5166 2237

**ploralis** (Guenée, 1854); valid (Aediodes) 5167 2280

propinqualis Möschler, 1880^54^; syn. (Desmia) 5164 2280

repandalis Schaus, 1920; syn. (Desmia) 2280

**desmialis** (Barnes & McDunnough, 1914); valid (Ercta) 5168 2238

kaeberalis (Haimbach, 1915); syn. (Hymenia) 5168 2238

**Diasemiodes** Munroe, 1957

**janassialis** (Walker, 1859); valid (Desmia) 5172 2304

hariolalis (Hulst, 1886); syn. (Botis) 5172 2304

**nigralis** (Fernald, 1892); valid (Pyrausta) 5173 2303

**eudamidasalis** (Druce, 1899)^29^, ^30^, ^55^; valid (Ischnurges) 5193 2306

**Diasemiopsis** Munroe, 1957

**leodocusalis** (Walker, 1859); valid (Lineodes) 5171 2307

**Diathrausta** Lederer, 1863

Tripodaula Meyrick, 1933

**reconditalis** (Walker, 1859); valid (Hymenia) 5174

minualis (Walker, [1866]); syn. (Aediodes) 5174

octomaculalis Fernald, 1887; syn. (Diathrausta) 5174

**harlequinalis** Dyar, 1913; valid (Diathrausta) 5175 2312

**a. montana** Haimbach, 1915; ssp. (Diathrausta) 5175

**b. amaura** Munroe, 1956; ssp. (Diathrausta) 5175

**c. lauta** Munroe, 1956; ssp. (Diathrausta) 5175 2312

**Ercta** Walker, 1859

**vittata** (Fabricius, 1794); valid (Phalaena) 5111 2319

hemialis (Guenée, 1854); syn. (Stenia) 5111 2319

tipulalis Walker, 1859; syn. (Ercta) 5111 2319

torquillalis (Möschler, 1890); syn. (Euclasta) 2319

valsaynalis (Kaye, 1925); syn. (Lineodes) 2319

**Hymenia** Hübner, [1825]

Zinckenia Zeller, 1852

**perspectalis** (Hübner, 1796); valid (Pyralis) 5169 2321

primordialis (Zeller, 1852)^5^; syn. (Zinckenia)

exportalis (Guenée, 1854)^5^; syn. (Spoladea)

phrasiusalis Walker, 1859; syn. (Hymenia) 5169 2321

rhinthonalis (Walker, 1859)^5^; syn. (Desmia)

spilotalis (Saalmüller, 1880)^5^; syn. (Spoladea)

**Spoladea** Guenée, 1854

**recurvalis** (Fabricius, 1775); valid (Phalaena) 5170 2322

fascialis (Stoll, 1782)^5^; syn. (Phalaena (Pyralis))

angustalis (Fabricius, 1787)^5^; syn. (Phalaena)

recurvella (Zincken, 1818); syn. emendation (Phycis) 2322

diffascialis (Hübner, 1825)^5^; syn. (Hymenia)

albifacialis (Boisduval, 1833)^5^; syn. (Hydrocampa)

animalis Guenée, 1854; syn. (Spoladea) 5170 2322

exodias (Meyrick, 1904)^56^; syn. (Hymenia)

ancylosema (Dognin, 1909); syn. (Nacoleia) 2322

formosana (Shiraki, 1910)^5^; syn. (Odezia)

**Loxostegopsis** Dyar, 1917

**polle** Dyar, 1917; valid (Loxostegopsis) 5115 2323

**xanthocrypta** (Dyar, 1913); valid (Pyrausta) 5116 2325

**merrickalis** (Barnes & McDunnough, 1918); valid (Pyrausta) 5117

**emigralis** (Barnes & McDunnough, 1918); valid (Pyrausta) 5118 2326

**curialis** Barnes & McDunnough, 1918; valid (Loxostegopsis) 5119 2327

**Nacoleia** Walker, 1859

Semioceros Meyrick, 1884

Aplomastix Warren, 1890

Orthocona Warren, 1896

**charesalis** (Walker, 1859)^57^; valid introduced (Botys)

molusalis (Walker, 1859); syn. (Botys)

vetustalis (Strand, 1918); syn. (Lampridia)

argillitis (Turner, 1937); syn. (Sylepta)

delhommealis (Legrand, 1966); syn. (Psara)

**Penestola** Möschler, 1890

**bufalis** (Guenée, 1854); valid (Stenia) 5179 2328

plebeialis (Walker, [1866]);

syn. preocc. by Lederer, 1863 (Botys) 2328

praeficalis Möschler, 1890; syn. (Penestola) 5179 2328

**simplicialis** (Barnes & McDunnough, 1913);

valid (Piletocera) 5180 2330

**Duponchelia** Zeller, 1847

**fovealis** Zeller, 1847^58^; valid introduced (Duponchelia)

canuisalis (Millière, 1868); syn. (Stenia)

eanuisalis Millière, 1869; syn. (Duponchelia?) misspelling?

griseata (Butler, 1875); syn. (Hymenia)

uniflexalis (Mabille, 1879); syn. (Stenia)

komiensis (Ghesquière, 1942); syn. (Decticogaster)

floeschlalis Legrand, 1965; syn. (Duponchelia)

**Steniodes** Snellen, 1875

Heringia Hedemann, 1894; preocc. by Rondani, 1856

Heringiella Berg, 1898; repl. name

Scaeocerandra Meyrick, 1936

**mendica** (Hedemann, 1894); valid (Heringia) 5178 2348

indianalis (Dyar, 1914); syn. (Stenia) 5178 2348

ceddalis (Schaus, 1924); syn. (Stenia) 2348

**Antigastra** Lederer, 1863

**catalaunalis** (Duponchel, 1833); valid (Botys) 5181 2375

venosalis (Walker, [1866]); syn. (Botys) 5181 2375

sionensis Caradja, 1929;

syn. infrasubsp. female (Antigastra) 2375

**Colomychus** Munroe, 1956

**talis** (Grote, 1878); valid (Botis) 5200 2379

**Condylorrhiza** Lederer, 1863

**vestigialis** (Guenée, 1854); valid (Botyodes) 5215 2380

illutalis (Guenée, 1854); syn. (Botys) 2380

tritealis (Walker, 1859); syn. (Botys) 5215 2380

mestoralis (Walker, 1859); syn. (Botys) 5215 2380

oratalis (Hulst, 1886); syn. (Eudioptis) 5215 2380

syleptalis (Strand, [1920]); syn. (Agathodes) 2380

**Diaphania** Hübner, 1818

Eudioptis Hübner, 1823

Diaphania Stephens, 1829; preocc. Hübner, 1818

Phakellura Guilding, 1830

Phacellura Agassiz, [1847]; emendation

Sestia Snellen, 1875

**olealis** (Felder, Felder & Rogenhofer, 1875);

valid (Eudioptis) 5201 2387

**nitidalis** (Stoll, 1781); valid (PhalaenaPyralis) 5202 2383

vitralis Hübner, 1818; syn. (Diaphania) 5202 2383

**arguta** (Lederer, 1863); valid (Phacellura) 5203 2410

**hyalinata** (Linnaeus, 1767); valid (PhalaenaGeometra) 5204 2434

marginalis (Stoll, 1781); syn. (PhalaenaPyralis) 5204 2434

lucernalis (Hübner, 1796); syn. (Pyralis) 5204 2434

hyalinatalis (Guenée, 1854);

syn. emendation (Phakellura) 5204 2434

sapillitalis (Weyenbergh, 1873); syn. (Pyralis) 5204 2434

**modialis** (Dyar, 1912); valid (Glyphodes) 5205 2418

**infimalis** (Guenée, 1854); valid (Phakellura) 5206 2419

**indica** (Saunders, 1851); valid (Eudioptes) 5207 2432

hyalinalis (Boisduval, 1833);

syn. not hyalinata L., 1767, emend. misident. (Botys) 5207 2432

capensis (Zeller, 1852); syn. (Eudioptis) 5207 2432

gazorialis (Guenée, 1854); syn. (Phakellura) 5207 2432

zygaenalis (Guenée, 1854); syn. (Phakellura) 5207 2432

cucurbitalis (Guenée, 1862); syn. (Phakellura) 5207 2432

intermedialis (Dognin, 1904)^5^; syn. (Glyphodes)

**lualis** (Herrich-Schäffer, 1871); valid (Botys) 5208 2393

**costata** (Fabricius, 1775)^30^, ^59^; valid (Phalaena) 5216 2458

aurocostalis (Guenée, 1854); syn. (Margarodes) 5216 2458

imitalis (Guenée, 1854); syn. (Margarodes) 2458

**Glyphodes** Guenée, 1854

Caloptychia Hübner, 1825^5^

Dysallacta Lederer, 1863^60^

Morocosma Lederer, 1863

**pyloalis** Walker, 1859; valid (Glyphodes) 5197 2478

sylpharis Butler, 1878; syn. (Glyphodes) 5197 2478

**sibillalis** Walker, 1859; valid (Glyphodes) 5198 2479

impuralis (Herrich-Schäffer, 1871); syn. (Botys) 2479

batesi Felder, Felder & Rogenhofer, 1875^61^;

syn. (Glyphodes) 2479

alitalis Hulst, 1886^ 62^; syn. (Glyphodes)

**a. berlandi** Munroe, 1956; ssp. extralimital (Glyphodes) 2479

**floridalis** (Fernald, 1901); valid (Marasmia) 5199 2480

**onychinalis** (Guenée, 1854)^63^; valid introduced (Asopia)

braurealis (Walker, 1859); syn. not checked (Zebronia)

astomalis (Felder, Felder & Rogenhofer) 1875;

syn. (Lepyrodes)

**Hoterodes** Guenée, 1854

**ausonia** (Cramer, 1777); valid (Phalaena) 5209 2495

canastralis (Hübner, [1825]);

syn. repl. name (Margaronia) 5209 2495

ausonialis Guenée, 1854; syn. emendation (Hoterodes) 5209 2495

**Leucochroma** Guenée, 1854

**corope** (Stoll, 1781); valid (Phalaena) 5210 2498

splendidalis (Stoll, 1781); syn. (Phalaena) 5210 2498

corrivalis (Hübner, [1825]); syn. repl. name (Epipagis) 5210 2498

coropealis Guenée, 1854;

syn. emendation (Leucochroma) 2498

selectalis (Walker, [1866]); syn. (Botys) 5210 2498

minoralis Warren, 1889; syn. (Leucochroma) 5210 2498

**Maruca** Walker, 1859

Siriocauta Lederer, 1863

**vitrata** (Fabricius, 1787)^64^; valid (Phalaena) 2504

testulalis (Geyer, 1832)^65^; syn. (Crochiphora) 2504

aquitilis (Guérin-Méneville, 1832); syn. (Hydrocampe) 2504

bifenestralis (Mabille, 1880); syn. (Botys) 2504

simialalis (Snellen, 1880)^5^; syn. (Siriocauta)

**Omiodes** Guenée, 1854

Lonchodes Guenée, 1854; preocc. by Gray, 1835

Spargeta Lederer, 1863

Coenostola Lederer, 1863

Hedylepta Lederer, 1863

Deba Walker, [1866]

Phycidicera Snellen, [1880]

Pelecyntis Meyrick, 1884

Charema Moore, 1888

Loxocreon Warren, 1892

Merotoma Meyrick, 1894

**indicata** (Fabricius, 1775)^66^; valid (Phalaena) 5212 2506

vulgalis (Guenée, 1854); syn. (Asopia) 5212 2506

sabalis (Walker, 1859); syn. (Botys) 5212 2506

moeliusalis (Walker, 1859); syn. (Botys) 5212 2506

connexalis (Walker, [1866]); syn. (Botys) 5212 2506

reductalis (Walker, [1866]); syn. (Botys) 5212 2506

dolosalis (Möschler, 1881); syn. (Botys) 2506

dnopheralis Mabille, 1900^5^; syn. (Omiodes)

lionnetalis (Legrand, 1966)^5^; syn. (Psara)

**rufescens** (Hampson, 1912); valid (Pilocrocis) 5213 2510

miamialis (Dyar, 1917); syn. (Sylepta) 5213 2510

**stigmosalis** (Warren, 1892); valid (Boeotarcha) 5214 2511

exogrammalis (Dyar, 1914)^67^; syn. (Boeotarcha) 2511

**martyralis** (Lederer, 1863)^68^; valid (Coenostola) 2524

vulpina (Meyrick, 1936); syn. (Hedylepta) 2524

cervinalis Amsel, 1956; syn. (Omiodes) 2520

**simialis** Guenée, 1854; valid (Omiodes) 5211 2527

jasonalis (Walker, 1859); syn. (Botys) 5211 2527

orontesalis (Walker, 1859); syn. (Botys) 5211 2527

eruptalis (Lederer, 1863); syn. (Coenostola) 5211 2527

**Palpita** Hübner, [1808]

Hapalia Hübner, 1818

Conchia Hübner, 1821

Margaronia Hübner, [1825]

Paradosis Zeller, 1852

Margarodes Guenée, 1854; preocc. by Guilding, 1829

Tobata Walker, 1859^5^

Sarothronota Lederer, 1863

Sebunta Walker, 1863

Ledereria Marschall, 1873; repl. name

Sylora Swinhoe, 1900^69^

Hvidodes Swinhoe, 1900; repl. name

Apyrausta Amsel, 1951

**flegia** (Cramer, 1777); valid (PhalaenaPyralis) 5217 2552

virginalis (Hübner, [1825]); syn. repl. name (Margaronia) 5217 2552

flegialis (Poey, 1832); syn. emendation (Pyralis) 5217 2552

villosalis (Zeller, 1852); syn. (Paradosis) 5217 2552

phantasmalis (Guenée, 1854); syn. (Margarodes) 5217 2552

flegyalis (Guenée, 1854); syn. emendation (Margarodes) 2552

**quadristigmalis** (Guenée, 1854); valid (Margarodes) 5218 2560

**persimilis** Munroe, 1959^70^; valid introduced (Palpita) 2561

**kimballi** Munroe, 1959; valid (Palpita) 5219 2557

**atrisquamalis** (Hampson, 1912); valid (Glyphodes) 5220 2544

gracilalis (Hulst, 1886);

syn. preocc. by (H.-S., 1871) (Botis) 5220 2544

**cincinnatalis** Munroe, 1952; valid (Palpita) 5221

**arsaltealis** (Walker, 1859); valid (Botys) 5222

**illibalis** (Hübner, 1818); valid (Hapalia) 5223 2542

euphaesalis (Walker, 1859)^71^; syn. (Botys) 5224 2542

subjectalis (Lederer, 1863);

syn. repl. name for euphaesalis (Walker, 1859) (Botys) 5224 2542

**maritima** Sullivan & Solis 2013^72^; valid (Palpita)

**freemanalis** Munroe, 1952; valid (Palpita) 5225 2543

**magniferalis** (Walker, 1861); valid (Botys) 5226

fascialis (Walker, 1862); syn. (Scoparia) 5226

guttulosa (Walker, 1863); syn. (Sebunta) 5226

**aenescentalis** Munroe, 1952; valid (Palpita) 5227

**Synclera** Lederer, 1863

**jarbusalis** (Walker, 1859); valid (Samea) 5196 2581

cottalis (Walker, 1859); syn. (Zebronia) 5196 2581

**Agathodes** Guenée, 1854

Stenurges Lederer, 1863

**designalis** Guenée, 1854; valid (Agathodes) 5240 2583

**a. monstralis** Guenée, 1854; ssp. (Agathodes) 5240 2583

floridalis (Hulst, 1886); syn. (Stenurges) 5240 2583

**Azochis** Walker, 1859

Catacteniza Möschler, 1890

**rufidiscalis** Hampson, 1904; valid (Azochis) 5232 2600

cubanalis Hampson, 1913; syn. (Azochis) 2600

**Compacta** Amsel, 1956

**capitalis** (Grote, 1881); valid (Botis) 5233 2602

posticata (Forbes, 1923);

syn. not G. & R., 1867 (Polygrammodes) 2602

**hirtalis** (Guenée, 1854); valid (Botys) 5234 2603

lybialis (Walker, 1859); syn. (Botys) 5234 2603

amatalis (Walker, 1859); syn. (Botys) 5234 2603

**hirtaloidalis** (Dyar, 1912); valid (Polygrammodes) 5235 2604

**Liopasia** Möschler, 1882

Terastiodes Warren, 1892

Dichotis Warren, 1892

**teneralis** (Lederer, 1863); valid (Botys) 5238 2636

**Polygrammodes** Guenée, 1854

Astura Guenée, 1854

Hilaopsis Lederer, 1863

Dichocrocopsis Dyar, 1910

**flavidalis** (Guenée, 1854); valid (Botys) 5228 2638

lacoalis (Walker, 1859); syn. (Botys) 5228 2638

cinctipedalis (Walker, [1866]); syn. (Botys) 5228 2638

**oxydalis** (Guenée, 1854)^73^; valid (Botys) 5228 2639

**langdonalis** (Grote, 1877); valid (Botis) 5229

**elevata** (Fabricius, 1777); valid (Phalaena) 5230 2689

elevalis (Guenée, 1854); syn. emendation (Astura) 5230 2689

grandimacula (Dognin, 1912); syn. (Sylepta) 5230 2689

**sanguinalis** Druce, 1895; valid (Polygrammodes) 5231 2652

**Terastia** Guenée, 1854

**meticulosalis** Guenée, 1854; valid (Terastia) 5239 2704

quadratalis (Walker, [1866])^5^; syn. (Megaphysa)

coeligenalis (Hulst, 1886); syn. (Megastes) 5239 2704

**Laniifera** Hampson, 1899

**cyclades** (Druce, 1895); valid (Pachynoa) 5236 2705

**Loxomorpha** Amsel, 1956^74^

Chrysobotys Munroe, 1956

**cambogialis** (Guenée, 1854); valid (Botys) 5154 3050

lucilla (Butler, 1878); syn. (Botys) 3050

citrinalis (Möschler, 1890); syn. (Botys) 3050

**flavidissimalis** (Grote, 1877); valid (Botis) 5155 3052

**Maracayia** Amsel, 1956^74^

**chlorisalis** (Walker, 1859); valid (Botys) 5298 3056

**Eulepte** Hübner, [1825]

Acrospila Lederer, 1863

**anticostalis** (Grote, 1871); valid (Botis) 5195 2719

levalis (Hulst, 1886); syn. (Botis) 5195 2719

**Ommatospila** Lederer, 1863

Thelda Walker, [1866]

**narcaeusalis** (Walker, 1859); valid (Leucochroma) 5294 2729

nummulalis Lederer, 1863; syn. (Ommatospila) 5294 2729

venustalis (Walker, [1866]); syn. (Leucinodes) 5294 2729

**Gonocausta** Lederer, 1863

**sabinalis** Dyar, 1914^75^; valid (Gonocausta) 2732

**Syllepis** Poey, 1832

**hortalis** (Walker, 1859); valid (Botys) 5283 2742

**Ategumia** Amsel, 1956

**ebulealis** (Guenée, 1854); valid (Samea) 5158 2749

**Diacme** Warren, 1892

**elealis** (Walker, 1859); valid (Samea) 5142 2758

taedialis (Walker, 1859); syn. (Samea) 5142 2758

**adipaloides** (Grote & Robinson, 1867); valid (Botys) 5143 2759

**phyllisalis** (Walker, 1859); valid (Samea) 5144 2761

aulicalis (Möschler, 1886); syn. (Botys) 2761

**mopsalis** (Walker, 1859); valid (Botys) 5145 2762

mettiusalis (Walker, 1859); syn. (Botys) 5145 2762

togalis (Lederer, 1863); syn. (Botys) 2762

griseicinctalis (Hampson, 1913); syn. (Paratalanta) 5145 2762

**Epipagis** Hübner, [1825]

Stenophyes Lederer, 1863

**forsythae** Munroe, 1955; valid (Epipagis) 5146 2770

**fenestralis** (Hübner, 1796)^76^; valid (Pyralis) 5147 2766

huronalis (Guenée, 1854); syn. (Samea) 5147 2766

serinalis (Walker, 1859); syn. (Phalangiodes) 5147 2766

**disparilis** (Dyar, 1910); valid (Stenophyes) 5148 2768

**Epipagis** Doubleday, [1849]

**submedialis** (Grote, 1876); valid (Botis) 5135 2774

dissectalis (Grote, 1880); syn. (Botis) 5135 2774

pilalis (Hulst, 1886); syn. (Botis) 5135 2774

**fuscimaculalis** (Grote, 1878); valid (Botis) 5136 2775

flavicoloralis (Grote, 1878); syn. (Botis?) 5136 2775

confovealis (Hulst, 1886); syn. (Botis) 5136 2775

**mustelinalis** (Packard, 1873); valid (Botys) 5137 2773

catenulalis (Grote, 1877); syn. (Botis) 5137 2773

monulalis (Hulst, 1886); syn. (Botis) 5137 2773

**luscitialis** (Barnes & McDunnough, 1914); valid (Pyrausta) 5138 2772

**Mimophobetron** Munroe, 1950

**pyropsalis** (Hampson, 1904); valid (Pyrausta) 5237 2776

rhodope (Hampson, 1913); syn. (Pyrausta) 5237 2776

liopasialis (Dyar, 1914); syn. (Pyrausta) 5237 2776

rhodopides (Strand, 1917); syn. infrasubspecific (Pyrausta) 5237 2776

**Mimorista** Warren, 1890

**subcostalis** (Hampson, 1913); valid (Sameodes) 5139 2784

**trimaculalis** (Grote, 1878); valid (Botis) 5140 2785

**tristigmalis** (Hampson, 1899); valid (Pilocrocis) 5141 2777

extremalis (Schaus, 1920); syn. (Psara) 2777

**Nomophila** Hübner, [1825]

Stenopteryx Duponchel, [1845]; preocc. by Meigen, 1830

Macronomeutis Meyrick, 1936

**triticalis** Berg, 1875^77^; valid (Nomophila) 2792

squalidalis Hampson, 1913; syn. (Nomophila) 2792

**nearctica** Munroe, 1973; valid (Nomophila) 5156 2794

**Samea** Guenée, 1854

Isopteryx Guenée, 1854; preocc. by Pictet, 1841

Pterygisus Butler, 1886; repl. name

**multiplicalis** (Guenée, 1854); valid (Isopteryx) 5151 2801

discessalis Walker, [1866]; syn. (Samea) 5151 2801

**baccatalis** (Hulst, 1892); valid (Loxostege) 5152 2804

**druchachalis** Dyar, 1924^78^; valid (Samea) 2810

**ecclesialis** Guenée, 1854; valid (Samea) 5150 2812

castellalis Guenée, 1854; syn. (Samea) 5150 2812

luccusalis Walker, 1859; syn. (Samea) 5150 2812

disertalis Walker, [1866]; syn. (Samea) 5150 2812

**Niphograpta** Warren, 1892

**albiguttalis** (Warren, 1889)^79^;

valid introduced (Epichronistis) 5149 2816

**Crocidocnemis** Warren, 1889

Somatania Möschler, 1890^80^

**pellucidalis** (Möschler, 1890); valid (Somatania) 5153 2818

suffusalis (Hampson, 1899); syn. (Sameodes) 2818

samealis (Dyar, 1914); syn. (Stenia) 2818

**Asciodes** Guenée, 1854

**gordialis** Guenée, 1854; valid (Asciodes) 5267 2823

quietalis (Walker, 1859); syn. (Scoparia) 5267 2823

confusalis (Hulst, 1886); syn. (Desmia) 5267 2823

**Psara** Snellen, 1875

Epichronistis Meyrick, 1886^5^

**obscuralis** (Lederer, 1863); valid (Botys) 5268 2851

**dryalis** (Walker, 1859); valid (Botys) 5269 2854

**Sathria** Lederer, 1863

**internitalis** (Guenée, 1854); valid (Asciodes) 5270 2863

stercoralis Lederer, 1863; syn. (Sathria) 5270 2863

serenalis (Walker, [1866]); syn. (Megaphysa) 5270 2863

**Conchylodes** Guenée, 1854

Ledereria Snellen, 1875; preocc. by Marschall, 1873

**diphteralis** (Geyer, 1832); valid (Lypotigris) 5290 2867

**salamisalis** Druce, 1895; valid (Conchylodes) 5291 2871

**ovulalis** (Guenée, 1854); valid (Spilomela) 5292 2878

**concinnalis** Hampson, 1899; valid (Conchylodes) 5293 2879

**Cnaphalocrocis** Lederer, 1863

Marasmia Lederer, 1863^81^

Dolichosticha Meyrick, 1884

Epimima Meyrick, 1886

Lasiacme Warren, 1896

Bradinomorpha Matsumura, 1920

Susumia Marumo, 1930

Prodotaula Meyrick, 1934

Neomarasmia Kalra, David, & Banerji, 1967;

unavailable (ICZN Art. 13)

**trapezalis** (Guenée, 1854); valid (Salbia) 5288 2896

creonalis (Walker, 1859); syn. (Botys) 5288 2896

neoclesalis (Walker, 1859); syn. (Botys) 5288 2896

suspicalis (Walker, 1859); syn. (Botys) 5288 2896

convectalis (Walker, [1866]); syn. (Botys) 5288 2896

bifurcalis Snellen, 1880; syn. (Cnaphalocrocis) 5288 2896

perinephes (Meyrick, 1886); syn. (Dolichosticha) 2896

andresi (Rebel, 1912)^82^; syn. (Bradina)

**cochrusalis** (Walker, 1859); valid (Hydrocampa) 5289 2895

azionalis (Walker, 1859); syn. (Botys) 5289 2895

ruptalis (Walker, [1866]); syn. (Botys) 5289 2895

perspersalis Möschler, 1890; syn. (Cnaphalocrocis) 2895

**Salbia** Guenée, 1854

Salbiomorpha Snellen, 1875^83^

**tytiusalis** (Walker, 1859); valid (Botys) 5285 2927

**mizaralis** (Druce, 1899); valid (Hedylepta) 5286 2916

**haemorrhoidalis** Guenée, 1854; valid (Salbia) 5287 2929

dircealis (Walker, 1859); syn. (Asopia) 5287 2929

futilalis (Barnes & McDunnough, 1914);

syn. (Hedylepta) 5287 2929

**Syngamia** Guenée, 1854

Ochlia Hübner, 1823^5^

**florella** (Stoll, 1781); valid (Tinea) 5284 2940

quinqualis (Hübner, 1823); syn. (Anania) 5284 2940

florellalis Guenée, 1854; syn. emendation (Syngamia) 5284 2940

**Apilocrocis** Amsel, 1956

**brumalis** (Barnes & McDunnough, 1914); valid (Sylepta) 5112 2943

**pimalis** (Barnes & Benjamin, 1926); valid (Sylepta) 5113 2949

**Diaphantania** Möschler, 1890

**impulsalis** (Herrich-Schäffer, 1871); valid (Botys) 5114 2954

**Bicilia** Amsel, 1956

**iarchasalis** (Walker, 1859); valid (Botys) 5271 2959

differalis (Walker, [1866]); syn. (Pyralis) 5271 2959

concinnalis (Möschler, 1890); syn. (Botys) 2959

vogli Amsel, 1956; syn. (Bicilia) 5271 2959

fuscalis Amsel, 1956; syn. (Bicilia) 2959

**Deuterophysa** Warren, 1889

Gonopionea Hampson, 1913

**fernaldi** Munroe, 1983; valid repl. name (Deuterophysa) 5123 3000

costimaculalis (Fernald, 1901);

syn. homonym preocc. by Warren, 1889 (Pyrausta) 5123 3000

**Cangetta** Moore, 1886

**micralis** (Hampson, 1907)^84^; valid (Deuterophysa) 3003

**Eurrhyparodes** Snellen, 1880

Molybdantha Meyrick, 1884

**lygdamis** Druce, 1902; valid (Eurrhyparodes) 5122 3022

**splendens** Druce, 1895^85^; valid (Eurrhyparodes) 3024

**Hydropionea** Hampson, 1917^86^

**oblectalis** (Hulst, 1886); valid (Botis) 5124 3038

eumoros (Dyar, 1917); syn. (Clupeosoma) 5124 3038

**fenestralis** (Barnes & McDunnough, 1914); valid (Diasemia) 5125 3037

**Microphysetica** Hanpson, 1917

Falx Amsel, 1956; preocc. by Gouan, 1770

Falcimorpha Amsel, 1957^87^; repl. name

**hermeasalis** (Walker, 1859); valid (Isopteryx) 3066

nymphulalis (Hampson, 1906); syn. (Parthenodes) 3056

philogelos (Dyar, 1922)^88^; syn. (Sufetula) 5121 3066

sinuosalis (Amsel, 1956); syn. (Falx) 5121 3056

**Palpusia** Amsel, 1956

**glaucusalis** (Walker, 1859)^89^; valid (Botys) 5269 3095

**goniopalpia** (Hampson, 1912); valid (Pilocrocis) 5297 3099

**Rhectocraspeda** Warren, 1892^90^

Pilemia Möschler, 1882; preocc. by Fairmaire, 1863

Rapoona Hedemann, 1894

**periusalis** (Walker, 1859); valid (Botys) 5157 3125

deformalis (Möschler, 1882); syn. (Pilemia) 5157 3125

tristis (Hedemann, 1894); syn. (Rapoona) 5157 3125

**Sisyracera** Möschler, 1890

Araschnopsis Amsel, 1956^91^

**subulalis** (Guenée, 1854); valid (Endotricha) 5194 3127

preciosalis (Möschler, 1882); syn. (Leucinodes) 5194 3127

**contortilinealis** (Hampson, 1895)^92^; valid (Samea) 3128

veroniae (Dyar, 1917); syn. (Nacoleia) 3128

**Odontiinae**

**Noctueliopsis** Munroe, 1961

**brunnealis** Munroe, 1972; valid (Noctueliopsis) 4830 1260

**puertalis** (Barnes & McDunnough, 1912); valid (Noctuelia) 4831 1261

**palmalis** (Barnes & McDunnough, 1918); valid (Noctuelia) 4832 1264

**atascaderalis** (Munroe, 1951); valid (Noctuelia) 4833

**aridalis** (Barnes & Benjamin, 1922); valid (Noctuelia) 4834 1265

**pandoralis** (Barnes & McDunnough, 1914); valid (Noctuelia) 4835 1266

**a. minimistricta** (Dyar, 1913); ssp. extralimital (Pyrausta) 1266

**rhodoxanthinalis** Munroe, 1974; valid (Noctueliopsis) 4836 1269

**bububattalis** (Hulst, 1886); valid (Botis) 4837 1267

tectalis (Barnes & McDunnough, 1914); syn. (Noctuelia) 4837 1267

**virula** (Barnes & McDunnough, 1918); valid (Noctuelia) 4838 1268

**Odontivalvia** Munroe, 1973

**radialis** (Munroe, 1972); valid (Noctueliopsis) 4829 1271

**Pogonogenys** Munroe, 1961

**proximalis** (Fernald, 1894); valid (Titanio) 4817 1272

**frechini** Munroe, 1961; valid (Pogonogenys) 4818

**masoni** Munroe, 1961; valid (Pogonogenys) 4819 1273

**Chrismania** Barnes & McDunnough, 1914

**pictipennalis** Barnes & McDunnough, 1914;

valid (Chrismania) 4820 1274

**Plumipalpiella** Munroe, 1995^93^; repl. name

Plumipalpia Munroe, 1961; preocc. by Hampson, 1898

**martini** (Munroe, 1961); valid (Plumipalpia) 4821 1275

**Nannobotys** Munroe, 1961

**commortalis** (Grote, 1881); valid (Botis) 4822 1276

minima (Dyar, 1917); syn. (Noctuelia) 4822 1276

**Psammobotys** Munroe, 1961

**fordi** Munroe, 1961; valid (Psammobotys) 4824 1277

**alpinalis** Munroe, 1972; valid (Psammobotys) 4825

**Heliothelopsis** Munroe, 1961

**arbutalis** (Snellen, 1875); valid (Aporodes) 4841 1278

rhea (Druce, 1894); syn. (Panameria) 4841 1278

**costipunctalis** (Barnes & McDunnough, 1914);

valid (Heliothela) 4842 1279

breiralis (Schaus, 1920); syn. (Pycnarmon) 1279

**unicoloralis** (Barnes & McDunnough, 1914);

valid (Heliothela) 4843 1280

**Mojaviodes** Munroe, 1972

**blanchardae** Munroe, 1972; valid (Mojaviodes) 4840 1281

**Mojavia** Munroe, 1961

**achemonalis** (Barnes & McDunnough, 1914);

valid (Noctuelia) 4839 1282

pulcharalis (Barnes & Benjamin, 1924);

syn. form (Noctuelia) 4839 1282

**Microtheoris** Meyrick, 1932

**vibicalis** (Zeller, 1873); valid (Botis) 4795 1283

**ophionalis** (Walker, 1859); valid (Rhodaria) 4796 1284

sesquialteralis (Zeller, 1873); syn. (Botis) 4796 1284

nasionalis (Zeller, 1873); syn. (Botis) 4796 1284

**a. lacustris** Munroe, 1961; ssp. (Microtheoris) 4796

**b. eremica** Munroe, 1961; ssp. (Microtheoris) 4796 1284

**c. baboquivariensis** Munroe, 1961; ssp. (Microtheoris) 4796 1284

**d. occidentalis** Munroe, 1961; ssp. (Microtheoris) 4796

**Rhodocantha** Munroe, 1961

**diagonalis** Munroe, 1961; valid (Rhodocantha) 4797 1285

**Frechinia** Munroe, 1961

**helianthiales** (Murtfeldt, 1897); valid (Titanio) 4798 1286

thyanalis (Druce, 1899); syn. (Pionea) 4798 1286

**murmuralis** (Dyar, 1917)^94^; valid (Titanio) 4798 1287

**lutosalis** (Barnes & McDunnough, 1914); valid (Titanio) 4799 1288

**laetalis** (Barnes & McDunnough, 1914); valid (Titanio) 4800 1289

**criddlealis** (Munroe, 1951); valid (Titanio) 4801

**texanalis** Munroe, 1961; valid (Frechinia) 4802 1290

**Procymbopteryx** Munroe, 1961

**belialis** (Druce, 1899); valid (Pionea) 4803 1291

**Cymbopteryx** Munroe, 1961

**fuscimarginalis** Munroe, 1961; valid (Cymbopteryx) 4804 1292

**unilinealis** (Barnes & McDunnough, 1918); valid (Loxostege) 4805 1293

phaeopasta (Hampson, 1919; syn. (Loxostegopsis) 4805 1293

**Neocymbopteryx** Munroe, 1973

**heitzmani** Munroe, 1973; valid (Neocymbopteryx) 4806

**Edia** Dyar, 1913

**semiluna** (Smith, 1905); valid (Lythrodes) 4807 1297

bidentalis (Barnes & McDunnough, 1912);

syn. (Cynaeda) 4807 1297

microstagma Dyar, 1913; syn. (Edia) 4807 1297

**minutissima** (Smith, 1906); valid (Lythrodes) 4808 1298

coolidgei Dyar, 1921; syn. (Edia) 4808 1298

**Dichozoma** Munroe, 1961

**parvipicta** (Barnes & McDunnough, 1918);

valid (Loxostege) 4809 1299

**Cuneifrons** Munroe, 1961

**coloradensis** Munroe, 1961; valid (Cuneifrons) 4810

**Gyros** Henry Edwards, 1881; repl. name

Oribates Henry Edwards, 1881; preocc. by Eugès, 1834

Monocona Warren, 1892

**muirii** (Henry Edwards, 1881); valid (Oribates) 4811

**a. rubralis** (Warren, 1892); ssp. (Monocona) 4811

**atripennalis** Barnes & McDunnough, 1914; valid (Gyros) 4812

**powelli** Munroe, 1959; valid (Gyros) 4813 1300

**Anatralata** Munroe, 1961

**versicolor** (Warren, 1892); valid (Aporodes) 4814 1301

**Anatralata** Munroe, 1972

**chemsaki** Munroe, 1972; valid (Eremanthe) 4815 1302

**Metaxmeste** Hübner, 1825

Hercyna Treitschke, 1828^95^

**nubicola** Munroe, 1954; valid (Metaxmeste) 4816

**Chlorobaptella** Munroe, 1995^96^; repl. name

Chlorobapta Barnes & McDunnough, 1914;

preocc. by Kraatz, 1880

**rufistrigalis** (Barnes & McDunnough, 1914);

valid (Chlorobapta) 4844 1303

**Mimoschinia** Warren, 1892

**rufofascialis** (Stephens, 1834);

valid extralimital, repl. name (Ennychia) 4826 1304

fascialis (Haworth, 1803);

syn. preocc. by Hübner, 1796 (Pyralis) 4826 1304

thalialis (Walker, 1859); syn. (Botys?) 4826 1304

perviana (Walker, 1865); syn. (Anthophila) 4826 1304

gelidalis (Walker, [1866]); syn. (Pyralis?) 4826 1304

costaemaculalis (Snellen, 1887); syn. (Thelcteria) 4826 1304

**a. novalis** (Grote, 1876); ssp. (Emprepes) 4826 1304

**b. decorata** (Druce, 1898); ssp. (Eustrotia) 4826 1304

**c. nuchalis** (Grote, 1878); ssp. (Emprepes) 4826 1304

**Porphyrorhegma** Munroe, 1961

**fortunata** Munroe, 1961; valid (Porphyrorhegma) 4823 1305

**Jativa** Munroe, 1961

**castanealis** (Hulst, 1886); valid (Orobena) 4827 1306

jativa (Barnes, 1905); syn. (Thalpochares) 4827 1306

**Pseudoschinia** Munroe, 1961

**elautalis** (Grote, 1881); valid (Eurycreon) 4828 1307

magnalis (Hulst, 1886); syn. (Emprepes) 4828 1307

**Glaucodontia** Munroe, 1972

**pyraustoides** Munroe, 1972; valid (Glaucodontia) 4845

**Cliniodes** Guenée, 1854

Idessa Walker, 1859

Exarcha Lederer, 1863

Metrea Grote, 1882^97^

Basonga Möschler, 1886^98^

Procliniodes Hayden, 2011^99^

**ostreonalis** (Grote, 1882); valid (Metrea) 4789

urticaloides (Fyles, 1894); syn. (Botys) 4789

**Glaphyriinae**^100^

**Schacontia** Dyar, 1914^101^

**themis** Solis & Goldstein, 2013; valid (Schacontia)^102^

**rasa** Solis & Goldstein, 2013; valid (Schacontia)^103^

**Alatuncusia** Amsel, 1956

**bergii** (Möschler, 1890); valid (Dichogama) 4793 800

**Dichogama** Lederer, 1863

Carbaca Walker, [1866]

**redtenbacheri** Lederer, 1863; valid (Dichogama) 4790 815

nigra Amsel, 1956; syn. infrasubspecific (Dichogama) 815

**amabilis** Möschler, 1890; valid (Dichogama) 4791 806

**colotha** Dyar, 1912; valid (Dichogama) 4792 811

**Eustixia** Hübner, 1823

Thelcteria Lederer, 1863

**pupula** Hübner, 1823; valid (Eustixia) 4794 638

**Hellula** Guenée, 1854

Oeobia Hübner, [1825]; suppressed (ICZN Op. 536)

Phyratocosma Meyrick, 1936

Ashwania Pajni & Rose, 1977^104^

**rogatalis** (Hulst, 1886); valid (Botis) 4846 639

**phidilealis** (Walker, 1859); valid (Leucochroma) 4847 640

trypheropa (Meyrick, 1936); syn. (Phryatocosma) 4847 640

**kempae** Munroe, 1972; valid (Hellula) 4848 641

**aqualis** Barnes & McDunnough, 1914; valid (Hellula) 4849 642

**subbasalis** (Dyar, 1923); valid (Lamprosema) 4850 643

**Glaphyria** Hübner, 1823

Homophysa Guenée, 1854

Berdura Möschler, 1886

**glaphyralis** (Guenée, 1854); valid (Homophysa) 4869 646

stipatalis (Walker, [1866]); syn. (Scopula) 4869 646

albolineata (Grote & Robinson, 1867); syn. (Lipocosma) 4869 646

**sequistrialis** Hübner, 1823; valid (Glaphyria) 4870 645

**basiflavalis** Barnes & McDunnough, 1913; valid (Glaphyria) 4871 647

**peremptalis** (Grote, 1878); valid (Homophysa) 4872 656

**fulminalis** (Lederer, 1863); valid (Homophysa) 4873 655

**cappsi** Munroe, 1972; valid (Glaphyria) 4874 650

**Upiga** Capps, 1964

**virescens** (Hulst, 1900); valid (Eromene) 4851 680

**Aethiophysa** Munroe, 1964

**delicata** Munroe, 1964; valid (Aethiophysa) 4875 690

**dualis** (Barnes & McDunnough, 1914); valid (Glaphyria) 4876 687

**invisalis** (Guenée, 1854)^105^; valid (Ebulea) 4877 681

lentiflualis (Zeller, 1872); syn. (Homophysa) 4877 681

**consimilis** Munroe, 1964; valid (Aethiophysa) 4878 682

**extorris** (Warren, 1892)^106^; valid (Hypolais) 5325 626

quadristrigalis (Fernald, 1894); syn. (Metasia) 5325 626

**Xanthophysa** Munroe, 1964

**psychialis** (Hulst, 1886); valid (Botis) 4879 694

**Abegesta** Munroe, 1964

**reluctalis** (Hulst, 1886); valid (Orobena) 4866 695

**remellalis** (Druce, 1899); valid (Homophysa) 4867 696

**concha** Munroe, 1964; valid (Abegesta) 4868 697

**Stegea** Munroe, 1964; repl. name

Egesta Ragonot, 1891; preocc. by Conrad, 1845

**mexicana** Munroe, 1964; valid (Stegea) 4859 705

**sola** Munroe, 1972; valid (Stegea) 4860 708

**simplicialis** (Kearfott, 1907); valid (Symphysa) 4861 700

**minutalis** (Walter, 1928); valid (Egesta) 4862 706

**powelli** Munroe, 1972; valid (Stegea) 4863 707

**eripalis** (Grote, 1878); valid (Homophysa) 4864 698

**salutalis** (Hulst, 1886); valid (Botis) 4865 699

**a. ochralis** (Haimbach, 1908); ssp. (Symphysa) 4865

**b. grisealis** Munroe, 1972; ssp. (Stegea) 4865 699

**c. riparialis** Munroe, 1972; ssp. (Stegea) 4865 699

**Paregesta** Munroe, 1964

**californiensis** Munroe, 1964; valid (Paregesta) 4852 709

**Nephrogramma** Munroe, 1964

**reniculalis** (Zeller, 1872); valid (Homophysa) 4857 711

**separata** Munroe, 1972; valid (Nephrogramma) 4858 712

**Scybalistodes** Munroe, 1964

**periculosalis** (Dyar, 1908); valid (Glaphyria) 4853 718

**vermiculalis** Munroe, 1964; valid (Scybalistodes) 4854 720

**regularis** Munroe, 1964; valid (Scybalistodes) 4855 719

**fortis** Munroe, 1972; valid (Scybalistodes) 4856 721

**Plumegesta** Munroe, 1972

**largalis** Munroe, 1972; valid (Plumegesta) 4880 722

**Ennomosia** Amsel, 1956

**basalis** (Hampson, 1897)^107^; valid (Clupeosoma) 3018

**Lipocosma** Lederer, 1863

Clarkeia Amsel, 1956; preocc. by Kozlowski, 1923

Clarkeiodes Amsel, 1957; repl. name

Lipocosmopsis Munroe, 1964

**sicalis** (Walker, 1859); valid (Leucinodes) 4881 749

perfusalis (Walker, [1866]); syn. (Pyralis) 4881 749

**diabata** Dyar, 1917; valid (Lipocosma) 4882 757

**adelalis** (Kearfott, 1903); valid (Symphysa) 4883

**intermedialis** Barnes & McDunnough, 1912;

valid (Lipocosma) 4884 762

**septa** Munroe, 1972; valid (Lipocosma) 4885 771

**albinibasalis** Munroe, 1995^108^; valid (Lipocosma) 750

albibasalis Barnes & McDunnough, 1911;

syn. preocc. by Hampson, 1906 (Lipocosma) 4886 750

**polingi** Munroe, 1972; valid (Lipocosma) 4887 768

**Lipocosmodes** Munroe, 1964

**fuliginosalis** (Fernald, 1888); valid (Lipocosma) 4888 773

**Dicymolomia** Zeller, 1872

Bifalculina Amsel, 1956

**julianalis** (Walker, 1859); valid (Cataclysta) 4889 792

decora Zeller, 1872; syn. (Dicymolomia) 4889 792

**metalophota** (Hampson, 1897); valid (Ambia) 4890 793

consortalis (Dyar, 1914); syn. (Lipocosma) 4890 793

argentipunctalis (Amsel, 1956); syn. (Bifalculina) 4890 793

**opuntialis** Dyar, 1908; valid (Dicymolomia) 4891 795

**metalliferalis** (Packard, 1873); valid (Calaclysta) 4892 796

sauberi Hedemann, 1883; syn. (Dicymolomia) 4892 796

**grisea** Munroe, 1964; valid (Dicymolomia) 4893 791

**micropunctalis** Munroe, 1964; valid (Dicymolomia) 4894 794

**Chalcoela** Zeller, 1872

**iphitalis** (Walker, 1859); valid (Cataclysta) 4895 798

aurifera Zeller, 1872; syn. (Chalcoela) 4895 798

**pegasalis** (Walker, 1859); valid (Cataclysta) 4896 799

principalis (Walker, [1866]); syn. (Cataclysta) 4896 799

egressalis (Walker, [1866]); syn. (Cataclysta) 4896 799

robinsonii (Grote, 1871); syn. (Cataclysta) 4896 799

discedalis Möschler, 1890; syn. (Chalcoela) 4896 799

**Evergestis** Hübner, [1825]^109^

Homochroa Hübner, [1825]

Mesographe Hübner, [1825]

Scopolia Hübner, [1825]

Pionea Duponchel, 1845

Orobena Guenée, 1854

Aedis Grote, 1878

Paraedis Grote, 1882

Maelinoptera Staudinger, 1893^110^

Pachyzancloides Matsumura, 1925

Reskovitsia Szent-Ivány, 1942^111^

**pallidata** (Hufnagel, 1767); valid (Phalaena) 4897

straminalis (Hübner, 1793); syn. (PhalaenaPyralis) 4897

elutalis (Hübner, 1796); syn. (Pyralis) 4897

stramentalis (Hübner, 1825); syn. (Mesographe) 4897

eunusalis (Walker, 1859); syn. (Pionea) 4897

**rimosalis** (Guenée, 1854); valid (Pionea) 4898 1334

**consimilis** Warren, 1892; valid (Evergestis) 4899 1335

**aridalis** Barnes & McDunnough, 1914; valid (Evergestis) 4900 1336

**unimacula** (Grote & Robinson, 1867); valid (Asopia) 4901

**lunulalis** Barnes & McDunnough, 1914; valid (Evergestis) 4902 1346

**nolentis** Heinrich, 1940; valid (Evergestis) 4903 1347

**simulatilis** (Grote, 1880); valid (Aedis) 4904 1349

brunneogrisea (Henry Edwards, 1886); syn. (Prorasea) 4904 1349

**angustalis** (Barnes & McDunnough, 1918);

valid (Phlyctaenia) 4905 1348

**a. catalinae** Munroe, 1974; ssp. (Evergestis) 4905

**b. arizonae** Munroe, 1974; ssp. (Evergestis) 4905 1348

**vinctalis** Barnes & McDunnough, 1914; valid (Evergestis) 4906 1350

**a. muricoloralis** Munroe, 1974; ssp. (Evergestis) 4906

**palousalis** Munroe, 1974^112^; valid (Evergestis) 4907

**a. obscuralias** Munroe, 1995; ssp. repl. name (Evergestis)

obscuralis Barnes & McDunnough, 1914;

syn. preocc. by (Hampson, 1912) (Evergestis) 4907

**comstocki** Munroe, 1974; valid (Evergestis) 4908 1351

**funalis** (Grote, 1878); valid (Aedis) 4909 1352

**a. insulalis** Barnes & McDunnough, 1914; ssp. (Evergestis) 4909

**b. columbialis** Munroe, 1974; ssp. (Evergestis) 4909

**c. angelina** Munroe, 1974; ssp. (Evergestis) 4909 1352

**d. wallacensis** Munroe, 1974; ssp. (Evergestis) 4909

**subterminalis** Barnes & McDunnough, 1914;

valid (Evergestis) 4910

**eurekalis** Barnes & McDunnough, 1914; valid (Evergestis) 4911

**a. fuscistrigalis** Munroe, 1974; ssp. (Evergestis) 4911

**obliqualis** (Grote, 1883); valid (Paraedis) 4912 1353

**dischematalis** Munroe, 1995^113^; valid repl. name (Evergestis) 1354

dimorphalis Munroe, 1974;

syn. preocc. Osthelder, 1938 (Evergestis) 4913 1354

**triangulalis** Barnes & McDunnough, 1914; valid (Evergestis) 4914 1355

**borregalis** Munroe, 1974; valid (Evergestis) 4915 1356

**Prorasea** Grote, 1878

**simalis** Grote, 1878; valid (Prorasea) 4916 1357

**gracealis** Munroe, 1974; valid (Prorasea) 4917 1358

**praeia** (Dyar, 1917); valid (Cornifrons) 4918

**fernaldi** Munroe, 1974; valid (Prorasea) 4919

**sideralis** (Dyar, 1917); valid (Cornifrons) 4920 1359

**pulveralis** (Warren, 1892); valid (Cornifrons) 4921

**Cornifrons** Lederer, 1858

Ventosalis Marion, 1957^5^

**actualis** Barnes & McDunnough, 1918; valid (Cornifrons) 4922

**phasma** Dyar, 1917; valid (Cornifrons) 4923 1360

chlorophasma Dyar, 1917; syn. (Cornifrons) 4923 1360

**Cylindrifrons** Munroe, 1951

**succandidalis** (Hulst, 1886); valid (Botis) 4924

simplex (Warren, 1895); syn. (Cavifrons) 4924

**Orenaia** Duponchel, 1845

**trivialis** Barnes & McDunnough, 1914; valid (Orenaia) 4925

subargentalis (Barnes & McDunnough, 1918);

syn. (Titanio) 4925

**coloradalis** Barnes & McDunnough, 1914; valid (Orenaia) 4926

**arcticalis** Munroe, 1974; valid (Orenaia) 4927

**sierralis** Munroe, 1974; valid (Orenaia) 4928

**alticolalis** (Barnes & McDunnough, 1914); valid (Titanio) 4929

**pallidivittalis** Munroe, 1956; valid (Orenaia) 4930

**macneilli** Munroe, 1974; valid (Orenaia) 4931

**Evergestella** Munroe, 1974

**evincalis** (Möschler, 1890); valid (Botys) 4932 1361

**Trischistognatha** Warren, 1892

**pyrenealis** (Walker, 1859); valid (Botys) 4933 1363

medonalis (Walker, 1859); syn. (Botys) 4933 1363

implicitalis (Möschler, 1890); syn. (Orobena) 1363

dyaralis (Fernald, 1901); syn. (Evergestis) 4933 1363

**Musotiminae**^114^

**Undulambia** Lange, 1956

**striatalis** (Dyar, 1906); valid (Ambia) 4740 942

**polystichalis** Capps, 1965; valid (Undulambia) 4741 934

**rarissima** Munroe, 1972; valid (Undulambia) 4742 941

**Neomusotima** Yoshiyasu, 1985

**conspurcatalis** (Warren, 1896)^115^; valid introduced (Ambia)

fuscalis (Snellen, 1901)^116^; syn. (Musotima)

**Scopariinae**

**Gesneria** Hübner, 1824-25

Scoparona Chapman, 1912

**centuriella** (Denis & Schiffermüller, 1775);

valid extralimital (Tinea) 4703

centurionalis Hübner, 1824-25;

syn. unjustified emendation (Gesneria) 4703

numeralis (Zetterstedt, 1839); syn. (Scopula) 4703

quadratella (Zetterstedt, 1839); syn. (Phycis) 4703

centurialis (Guenée, 1854); syn. emendation (Scoparia) 4703

confluella (Krulikowsky, 1908);

syn. form infrasubspecific (Scoparia) 4703

obscura (Caradja, 1916)^5^;

syn. aberration infrasubspecific (Scoparia)

**a. borealis** (Duponchel, 1836); ssp. (Eudorea) 4703

**b. caecalis** (Walker, 1859); ssp. (Hypena) 4703

caliginosalis (Walker, [1866]); syn. (Scopula) 4703

**c. beringiella** Munroe, 1972; ssp. (Gesneria) 4703

**d. ninguidalis** (Hulst, 1886); ssp. (Scoparia) 4703

**e. sachalinensis** Matsumura, 1925^5^;

ssp. extralimital (Gesneria)

**rindgeorum** Munroe, 1972; valid (Gesneria) 4704

**Cosipara** Munroe, 1972

**tricoloralis** (Dyar, 1904); valid (Scoparia) 4705 823

**modulalis** Munroe, 1972; valid (Cosipara) 4706 824

**chiricahuae** Munroe, 1972; valid (Cosipara) 4707 825

**Scoparia** Haworth, 1811

Scopea Haworth, 1828

Eudorea Curtis, 1827

Phegea Gistel, 1848^5^

Epileucia Stephens, 1852^5^

Tetraprosopus Butler, 1882

Xeroscopa Meyrick, 1884

Sineudonia Leraut, 1986^117^

**rigidalis** Barnes & McDunnough, 1912; valid (Scoparia) 4708 829

**denigata** Dyar, 1929; valid (Scoparia) 4709 830

**normalis** Dyar, 1904; valid (Scoparia) 4710 831

**palloralis** Dyar, 1906; valid (Scoparia) 4711 834

obispalis Dyar, 1906; syn. (Scoparia) 4711 834

cervalis McDunnough, 1927; syn. (Scoparia) 4711 834

**californialis** Munroe, 1972; valid (Scoparia) 4712 835

**apachealis** Munroe, 1972; valid (Scoparia) 4713 836

**a. pinalensis** Munroe, 1972; ssp. (Scoparia) 4713 836

**b. utalis** Munroe, 1972; ssp. (Scoparia) 4713

**ruidosalis** Munroe, 1972; valid (Scoparia) 4714 837

**blanchardi** Munroe, 1972; valid (Scoparia) 4715 838

**biplagialis** Walker, [1866]; valid (Scoparia) 4716

libella Grote, 1878; syn. (Scoparia) 4716

**a. bellaeislae** Munroe, 1972; ssp. (Scoparia) 4716

**b. fernaldalis** Dyar, 1904; ssp. (Scoparia) 4716

**c. pacificalis** Dyar, 1921; ssp. (Scoparia) 4716

alaskalis Barnes & Benjamin, 1922; syn. (Scoparia) 4716

**d. afognakalis** Munroe, 1972; ssp. (Scoparia) 4716

**penumbralis** Dyar, 1906; valid (Scoparia) 4717

**cinereomedia** Dyar, 1904; valid (Scoparia) 4718

truncatalis McDunnough, 1923; syn. (Scoparia) 4718

**basalis** Walker, [1866]; valid (Scoparia) 4719

**dominicki** Munroe, 1972; valid (Scoparia) 4720

**huachucalis** Munroe, 1972; valid (Scoparia) 4721

**Eudonia** Billberg, 1820

Boiea Zetterstedt, 1839^5^

Eudoria Chapman, 1912

Witlesia Chapman, 1912

Dipleurina Chapman, 1912^118^

Malageudonia Leraut, 1989^118^

Vietteina Leraut, 1989^118^

**rectilinea** (Zeller, 1874); valid (Scoparia) 4722 860

refugalis (Hulst, 1886); syn. (Scoparia) 4722 860

nominatalis (Hulst, 1886); syn. (Scoparia) 4722 860

**commortalis** (Dyar, 1921); valid (Scoparia) 4723

**expallidalis** (Dyar, 1906); valid (Scoparia) 4724 861

rufitinctalis (Hampson, 1907); syn. (Scoparia) 4724 861

**franciscalis** Munroe, 1972; valid (Eudonia) 4725

**torniplagalis** (Dyar, 1904); valid (Scoparia) 4726 862

**a. alialis** (Barnes & McDunnough, 1912); ssp. (Scoparia) 4726 862

**b. perfectalis** Munroe, 1972; ssp. (Eudonia) 4726 862

**albertalis** (Dyar, 1929); valid (Scoparia) 4727

**vivida** Munroe, 1972; valid (Eudonia) 4728

**spaldingalis** (Barnes & McDunnough, 1912); valid (Scoparia) 4729

**spenceri** Munroe, 1972; valid (Eudonia) 4730 863

**rotundalis** Munroe, 1972; valid (Eudonia) 4731 864

**franclemonti** Munroe, 1972; valid (Eudonia) 4732 865

**schwarzalis** (Dyar, 1906); valid (Scoparia) 4733 867

**leucophthalma** (Dyar, 1929); valid (Scoparia) 4734

**a. petaluma** Munroe, 1972; ssp. (Eudonia) 4734

**echo** (Dyar, 1929); valid (Scoparia) 4735

**a. gartrelli** Munroe, 1972; ssp. (Eudonia) 4735

**bronzalis** (Barnes & Benjamin, 1922); valid (Scoparia) 4736 872

**alpina** (J. Curtis, 1850)^119^; valid (Eudorea)

borealis (Tengström, 1848); syn. (Eudorea)

gracilalis (Stainton, 1855); syn. (Eudorea)

lugubralis (Walker, [1866]); syn. (Scoparia) 4737

lapponica (Caradja, 1916); syn. variety (Scoparia)

phycitinalis (Dyar, 1929); syn. (Scoparia) 4737

persimilalis (McDunnough, 1961); syn. (Eudoria) 4737

japanalpina Inoue, 1982; syn. (Eudonia)

**a. madgei** Munroe, 1972; ssp. (Eudonia) 4737

**strigalis** (Dyar, 1906); valid (Scoparia) 4738 878

**heterosalis** (McDunnough, 1961); valid (Eudoria) 4739 879

**Crambinae**^120^, ^121^

**Hemiplatytes** Barnes & Benjamin, 1924

Alamogordia Dyar & Heinrich, 1927

**epia** (Dyar, 1912); valid (Diatraea) 5507 187

damon (Barnes & McDunnough, 1918); syn. (Platytes) 5507 187

**prosenes** (Dyar, 1912); valid (Diatraea) 5508 188

**parallela** (Kearfott, 1908); valid (Diatraea) 5509 189

**Eufernaldia** Hulst, 1900

**cadarellus** (Druce, 1896); valid (Crambus) 5338 1

argenteonervella Hulst, 1900; syn. (Eufernaldia) 5338 1

**Surattha** Walker, 1863

**santella** Kearfott, 1908^122^; valid (Surattha) 5326 7

**indentella** Kearfott, 1908^122^; valid (Surattha) 5327 6

**Prionapteryx** Stephens, 1834

Prionopteryx Zeller, 1863; emendation

Nuarace Walker, 1863

Pindicitora Walker, 1863^5^

Calarina Walker, 1866

Hypotomorpha Rebel, 1892

Platytesia Strand, 1918^5^

Loxophantis Meyrick, 1935^123^

Alloea Turner, 1947^124^; preocc. by Haliday, 1833

**yavapai** (Kearfott, 1908); valid (Eugrotea) 5332 17

**nebulifera** Stephens, 1834; valid (Prionapteryx) 5333 15

**achatina** Zeller, 1863; valid (Prionopteryx) 5334 9

delectalis (Hulst, 1886)^5^; syn. (Crambus) 5334 9

**cuneolalis** (Hulst, 1886); valid (Crambus) 5335 16

**serpentella** Kearfott, 1908; valid (Prionapteryx) 5336

**Mesolia** Ragonot, 1889

Eugrotea Fernald, 1896

Euparolia Dyar, 1914

Deuterolia Dyar, 1914

**baboquivariella** (Kearfott, 1907); valid (Prionapteryx) 5328 20

**oraculella** Kearfott, 1908; valid (Mesolia) 5329 22

**huachucaella** Kearfott, 1908^125^; valid (Mesolia) 5330 21

**incertellus** (Zincken, 1821); valid (Chilo) 5331 23

olivella (Grote, 1881); syn. (Prionopteryx) 5331 23

dentella (Fernald, 1896); syn. (Eugrotea) 5331 23

**Pseudoschoenobius** Fernald, 1891

**opalescalis** (Hulst, 1886); valid (Schoenobius) 5337 5

griseosparsa (Hampson, 1896); syn. (Prionopteryx) 5337 5

**Thopeutis** Hübner, 1818

Tetrachila Hübner, 1808^5^;

name rejected, ICZN Opinion 789

Cephis Ragonot, 1892

Stenochilo Hampson, 1896

Hombergia de Joannis, 1910

**forbesellus** (Fernald, 1896); valid (Chilo) 5473

**Occidentalia** Dyar & Heinrich, 1927^126^

**comptulatalis** (Hulst, 1886); valid (Crambus) 5474

**Xubida** Schaus, 1922

**linearellus** (Zeller, 1863); valid (Crambus) 5499 142

multilineatella (Hulst, 1887); syn. (Spermatophthora) 5499 142

**panalope** (Dyar, 1917); valid (Platytes) 5500 143

acerata (Dyar, 1917); syn. (Platytes) 5500 143

**relovae** Klots, 1970; valid (Xubida) 5501 144

**punctilineella** (Barnes & McDunnough, 1913);

valid (Platytes) 5502 145

**lipan** Klots, 1970; valid (Xubida) 5503 146

**dentilineatella** (Barnes & McDunnough, 1913);

valid (Platytes) 5504 147

**puritellus** (Kearfott, 1908); valid (Chilo) 5505 148

dinephelalis (Dyar, 1917); syn. (Platytes) 5505 148

**chiloidellus** (Barnes & McDunnough, 1913);

valid (Crambus) 5506 149

**Haimbachia** Dyar, 1909

**squamulellus** (Zeller, 1881); valid (Chilo) 5482 156

**arizonensis** Capps, 1965; valid (Haimbachia) 5483 157

**pallescens** Capps, 1965; valid (Haimbachia) 5484 158

**indistinctalis** Capps, 1965; valid (Haimbachia) 5485 159

**discalis** Dyar & Heinrich, 1927; valid (Haimbachia) 5486 150

**floridalis** Capps, 1965; valid (Haimbachia) 5487 160

**albescens** Capps, 1965; valid (Haimbachia) 5488

**placidellus** (Haimbach, 1907); valid (Crambus) 5489

**cochisensis** Capps, 1965; valid (Haimbachia) 5490 161

**diminutalis** Capps, 1965; valid (Haimbachia) 5491 162

**Eoreuma** Ely, 1910

**densellus** (Zeller, 1881); valid (Chilo) 5492 166

**loftini** (Dyar, 1917); valid (Chilo) 5493 164

opinionellus (Dyar, 1917); syn. (Chilo) 5493 164

**evae** Klots, 1970; valid (Eoreuma) 5494 167

**confederata** Klots, 1970; valid (Eoreuma) 5495 169

**multipunctellus** (Kearfott, 1908); valid (Chilo) 5496 170

**callista** Klots, 1970; valid (Eoreuma) 5497 171

**crawfordi** Klots, 1970; valid (Eoreuma) 5498

**arenella** A. Blanchard & Knudson, 1983^127^; valid (Eoreuma) 172

**Epina** Walker, 1866

Diatraenopsis Dyar & Heinrich, 1927

**dichromella** Walker, 1866; valid (Epina) 5468 186

differentialis (Fernald, 1888); syn. (Diatraea) 5468 186

matanzalis (Schaus, 1922); syn. (Chilo) 5468 186

**alleni** (Fernald, 1888); valid (Diatraea) 5469 185

**Chilo** Zincken, 1817

Chilona Sodoffsky, 1837

Borer Guenée, 1862

Diphryx Grote, 1881

Nephalia Turner, 1911

Hypiesta Hampson, 1919

Silveria Dyar, 1925

Chilotraea Kapur, 1950

**plejadellus** Zincken, 1821; valid (Chilo) 5470 192

sabulifera (Walker, 1863); syn. (Jartheza) 5470 192

prolatella (Grote, 1881); syn. (Diphryx) 5470 192

oryzaeellus Riley, 1882; syn. (Chilo) 5470 192

**erianthalis** Capps, 1963; valid (Chilo) 5471 191

**demotella** Walker, 1866; valid (Chilo) 5472 193

idalis (Fernald, 1896); syn. (Diatraea) 5472 193

fernaldalis Dyar & Heinrich, 1927; syn. (Chilo) 5472 193

**Diatraea** Guilding, 1828

Iesta Dyar, 1909

Diatraerupa Schaus, 1913

Trinidadia Dyar & Heinrich, 1927

Eodiatraea Box, 1953

Crambidiatraea Box & Capps, 1955

Zeadiatraea Box, 1955

**saccharalis** (Fabricius, 1794); valid (Phalaena) 5475 245

sacchari (Fabricius, 1798);

syn. emendation (Phalaena) 5475 245

sacchari Guilding, 1828;

secondary homonym preocc. by (F., 1798) (Diatraea) 245

leucaniellus (Walker, 1863); syn. (Crambus) 245

lineosellus (Walker, 1863); syn. (Crambus) 245

obliteratellus (Zeller, 1863); syn. (Chilo) 245

grenadensis Dyar, 1911; syn. (Diatraea) 245

pedidocta Dyar, 1911; syn. (Diatraea) 245

continens Dyar, 1911; syn. (Diatraea) 245

brasiliensis Gorkum & Waal, 1913; syn. (Diatraea) 245

incomparella Dyar & Heinrich, 1927; syn. (Diatraea) 245

**crambidoides** (Grote, 1880); valid (Chilo) 5476 213

zeacolella Dyar, 1911; syn. (Diatraea) 5476 213

tripsacicola Dyar, 1921; syn. (Diatraea) 5476 213

**mitteri** Solis, 2015^128^; valid (Diatraea)

**venosalis** (Dyar, 1917); valid (Haimbachia) 5477 253

**evanescens** Dyar, 1917; valid (Diatraea) 5478 216

sobrinalis Schaus, 1922; syn. (Diatraea) 5478 216

**grandiosella** Dyar, 1911; valid (Diatraea) 5479 220

**lineolata** (Walker, 1856); valid (Leucania) 5480 228

culmicolellus (Zeller, 1863); syn. (Chilo) 228

neuricellus (Zeller, 1863); syn. (Chilo) 228

pallidostricta Dyar, 1911; syn. (Diatraea) 228

**lisetta** (Dyar, 1909); valid (Iesta) 5481 229

cancellalis (Dyar, 1914); syn. (Iesta) 229

adulcia (Dyar, 1916); syn. (Iesta) 229

**Argyria** Hübner, 1818^129^, ^130^

**nummulalis** Hübner, 1818; valid (Argyria) 5460 64

argentana (Martyn, 1797)^131^;

syn. nomen nudum (Tortrix) 64

argentana Fernald, 1896;

syn. preocc. by (D. & S., 1775) (Argyria) 5460 64

**subaenescens** (Walker, 1863); valid (Urola) 5461 65

fuscipes (Zeller, 1863); syn. (Catharylla) 5461 65

**rufisignella** (Zeller, 1872); valid (Catharylla) 5462 66

rileyella Dyar, 1913; syn. (Argyria) 5462 66

**lacteella** (Fabricius, 1794); valid (Tinea) 5463 52

albana (Fabricius, 1798); syn. (Pyralis) 5463 52

pusillalis Hübner, 1818; syn. (Argyria) 5463 52

abronalis (Walker, 1859); syn. nomen dubium (Zebronia) 52

gonogramma Dyar, 1915; syn. (Argyria) 52

**auratellus** (Clemens, 1860); valid (Crambus) 5465 73

pulchella (Walker, 1863); syn. (Urola) 5465 73

**critica** Forbes, 1920; valid (Argyria) 5466 74

**tripsacas** (Dyar, 1921); valid (Crambus) 5467 84

**Urola** Walker, 1863

**nivalis** (Drury, 1773); valid (PhalaenaPyralis) 5464 93

argentata (Emmons, 1854); syn. (Geometra) 5464 93

michrochysella Walker, 1863; syn. (Urola) 5464 93

**Microcausta** Hampson, 1895

**flavipunctalis** Barnes & McDunnough, 1913;

valid (Microcausta) 5456 47

**bipunctalis** Barnes & McDunnough, 1914;

valid (Microcausta) 5457 48

**Diptychophora** Zeller, 1866

Scissolia Barnes & McDunnough, 1914

Colimaea Dyar, 1925

Mysticomima Meyrick, 1931

**harlequinalis** (Barnes & McDunnough, 1914);

valid (Scissolia) 5458 32

**incisalis** (Dyar, 1925); valid (Colimaea) 5459 30

**Euchromius** Guenée, 1845

Eromene Hübner, [1825]; preocc. by Hübner, 1821

Ommatopteryx Kirby, 1897

Pseudoancylolomia Ahmad, Zaidi, & Kamaluddin, 1982^132^

**ocellea** (Haworth, 1811); valid (Palparia) 5454 312

cyrilli (O.-G. Costa, 1829)^5^; syn. (Crambus)

funiculella (Treitschke, 1832)^5^; syn. (Phycis)

texana (Robinson, 1870); syn. (Eromene) 5454 312

gigantea (Turati, 1924); syn. (Eromene) 312

qadrii (Ahmad, Zaidi, & Kamaluddin, 1982)^132^;

syn. (Pseudoancylolomia)

**californicalis** (Packard, 1873); valid (Eromene) 5455

**Platytes** Guenée, 1845

Nagahama Marumo, 1933^5^

**vobisne** Dyar, 1920^133^; valid (Platytes) 5394

**Catoptria** Hübner, 1825

Tetrachila Hübner, 1806; unavailable

Exoria Hübner, 1825

**trichostomus** (Christoph, 1858); valid (Crambus) 5406

albisinuatella (Packard, 1867); syn. (Eudorea) 5406

tristis Kirpichnikova, 1994^134^; syn. (Catoptria)

**maculalis** (Zetterstedt, 1839); valid (Scopula) 5407

cacuminellus (Zeller, 1850)^5^; syn. (Crambus)

albimaculella (Burmann, 1948)^5^; syn. aberration (Crambus)

albisignata (Burmann, 1948)^5^; syn. aberration (Crambus)

**latiradiellus** (Walker, 1863); valid (Crambus) 5408

interruptus (Grote, 1877); syn. (Crambus) 5408

**oregonicus** (Grote, 1880); valid (Crambus) 5409

bartellus (Barnes & McDunnough, 1918); syn. (Crambus) 5409

**Thaumatopsis** Morrison, 1874

Propexus Grote, 1880

**edonis** (Grote, 1880); valid (Crambus (Propexus)) 5438 459

**pexellus** (Zeller, 1863); valid (Crambus) 5439 460

macropterellus (Zeller, 1863); syn. (Crambus) 5439 460

longipalpus Morrison, 1874; syn. (Thaumatopsis) 5439 460

**a. gibsonella** Kearfott, 1908; ssp. (Thaumatopsis) 5439

**b. coloradella** Kearfott, 1908; ssp. (Thaumatopsis) 5439 460

**c. strictalis** (Dyar, 1914); ssp. (Ubida) 5439 460

idion Dyar, 1919; syn. (Thaumatopsis) 5439 460

**magnificus** (Fernald, 1891); valid (Propexus) 5440 462

**fernaldella** Kearfott, 1905; valid (Thaumatopsis) 5441 463

**a. nortella** Kearfott, 1905; ssp. (Thaumatopsis) 5441

**b. lagunella** Dyar, 1912; ssp. (Thaumatopsis) 5441 463

**atomosella** Kearfott, 1908; valid (Thaumatopsis) 5442 464

**floridella** Barnes & McDunnough, 1913;

valid (Thaumatopsis) 5443 465

**fieldella** Barnes & McDunnough, 1912;

valid (Thaumatopsis) 5444 466

**repandus** (Grote, 1880); valid (Crambus (Propexus)) 5445 467

**crenulatella** Kearfott, 1908; valid (Thaumatopsis) 5446 468

**pectinifer** (Zeller, 1877); valid (Crambus) 5447 470

**actuellus** Barnes & McDunnough, 1918;

valid (Thaumatopsis) 5448 469

**solutellus** (Zeller, 1863); valid (Crambus) 5449

striatellus Fernald, 1896; syn. (Thaumatopsis) 5449

daeckeellus Kearfott, 1903; syn. (Thaumatopsis) 5449

**bolterellus** (Fernald, 1887)^135^; valid (Crambus) 5452

**digrammellus** (Hampson, 1919)^136^; valid (Crambus) 434

**Tehama** Hulst, 1888

**bonifatella** (Hulst, 1887); valid (Spermatophthora) 5453 458

inornatellus (Walker, 1863); syn. (Crambus) 5453 458

nevadellus (Kearfott, 1908); syn. (Crambus) 5453 458

**Fissicrambus** Bleszynski, 1963

**albilineellus** (Fernald, 1893)^137^; valid (Crambus) 5384

**quadrinotellus** (Zeller, 1877)^138^; valid (Crambus) 5385

**fissiradiellus** (Walker, 1863); valid (Crambus) 5430 441

curtellus (Walker, 1863); syn. (Crambus) 5430 441

gestatellus (Möschler, 1890); syn. (Crambus) 5430 441

**profanellus** (Walker, 1866); valid (Crambus) 5431 442

**intermedius** (Kearfott, 1908); valid (Crambus) 5432 443

**haytiellus** (Zincken, 1821); valid (Chilo) 5433 444

**hemiochrellus** (Zeller, 1877); valid (Crambus) 5434 445

**mutabilis** (Clemens, 1860); valid (Crambus) 5435 455

fuscicostellus (Zeller, 1863); syn. (Crambus) 5435 455

**minuellus** (Walker, 1863)^30^, ^133^; valid (Crambus) 5437 456

santiagellus (Schaus, 1922); syn. (Crambus) 5437 456

habanella (Schaus, 1922); syn. (Culladia) 5437 456

**Microcrambus** Bleszynski, 1963

**copelandi** Klots, 1968; valid (Microcrambus) 5418 496

**biguttellus** (Forbes, 1920); valid (Crambus) 5419 492

**elegans** (Clemens, 1860); valid (Crambus) 5420 500

terminellus (Zeller, 1863); syn. (Crambus) 5420 500

**polingi** (Kearfott, 1908); valid (Crambus) 5421 520

**minor** (Forbes, 1920); valid (Crambus) 5422 516

**discludellus** (Möschler, 1890); valid (Crambus) 5423 499

micralis (Hampson, 1919; syn. (Crambus) 5423 499

domingellus (Schaus, 1922); syn. (Crambus) 5423 499

discobolus Bleszynski, 1963; syn. (Microcrambus) 5423 499

**kimballi** Klots, 1968; valid (Microcrambus) 5424 512

**matheri** Klots, 1968; valid (Microcrambus) 5425 514

**croesus** Bleszynski, 1967; valid (Microcrambus) 5426 497

**Loxocrambus** Forbes, 1920

**coloradellus** (Fernald, 1893)^139^; valid (Crambus) 5387

**canellus** Forbes, 1920; valid (Loxocrambus) 5427

**mohaviellus** Forbes, 1920; valid (Loxocrambus) 5428

**awemensis** McDunnough, 1929; valid (Loxocrambus) 5429

**hospition** (Bleszynski, 1963)^140^; valid (Fissicrambus) 5436 457

**Neodactria** B. Landry, 1995^141^

**luteolellus** (Clemens, 1860); valid (Crambus) 5379 419

duplicatus (Grote, 1880); syn. (Crambus) 5379 419

holochrellus (Fernald, 1896)^5^;

syn. nomen nudum (Crambus)

**a. refotalis** (Hulst, 1886); ssp. (Crambus) 5379 419

ulae (Cockerell, 1888); syn. (Crambus) 5379 419

edredellus (Schaus, 1922); syn. (Crambus) 419

**zeellus** (Fernald, 1885); valid (Crambus) 5380 420

**caliginosellus** (Clemens, 1860); valid (Crambus) 5381 421

**murellus** (Dyar, 1904); valid (Crambus) 5382

simpliciellus (Kearfott, 1908); syn. (Crambus) 5382

**modestellus** (Barnes & McDunnough, 1918); valid (Crambus) 5383 422

**glenni** B. Landry & Metzler, 2002^142^; valid (Neodactria)

**daemonis** B. Landry & R. Brown, 2005^143^; valid (Neodactria)

**oktibbeha** B. Landry & R. Brown, 2005^143^; valid (Neodactria)

**cochisensis** B. Landry & Albu, 2012^144^; valid (Neodactria)

**Arequipa** Walker, 1863

**turbatella** Walker, 1863; valid (Arequipa) 5392

bipunctellus (Zeller, 1863); syn. (Crambus) 5392

**Pediasia** Hübner, [1825]

Carvanca Walker, 1856

Pseudopediasia Ganev, 1987^145^

Oseriates Fazekas, 1991^146^

**aridella** (Thunberg, 1794); valid (Tinea) 5410

salinellus (Tutt, 1887)^5^; syn. (Crambus)

kenderesiensis Fazekas, 1987^5^; syn. (Pediasia)

**a. edmontellus** (McDunnough, 1923); ssp. (Crambus) 5410

**b. caradjaellus** (Rebel, 1907)^5^; ssp. extralimital (Crambus)

monotonus (Filipjev, 1927)^5^; syn. extralimital (Crambus)

nepos (Rothschild, 1911)^5^; syn. extralimital (Crambus)

kasyi Ganev, 1983^5^; syn. extralimital (Pediasia)

mikkolai (Ganev, 1987)^147^;

syn. extralimital (Pseudopediasia)

**c. ludovicellus** (Marion, 1952)^5^; ssp. extralimital (Crambus)

**truncatellus** (Zetterstedt, 1839); valid (Chilo) 5411

lienigiellus (Zeller, 1843); syn. (Crambus) 5411

abtrusellus (Walker, 1863); syn. (Crambus) 5411

rufinalis (Walker, [1866]); syn. (Hypena) 5411

**browerellus** (Klots, 1942); valid (Crambus) 5412

**a. katahdini** (Klots, 1942); ssp. (Crambus) 5412

**trisecta** (Walker, 1856); valid (Carvanca) 5413

interminellus (Walker, 1863); syn. (Crambus) 5413

fuscisquamellus (Zeller, 1863)^5^;

syn. nomen nudum (Crambus)

exsiccatus (Zeller, 1863); syn. (Crambus) 5413

biliturellus (Zeller, 1874); syn. (Crambus) 5413

**laciniellus** (Grote, 1880); valid (Crambus) 5414

**ericellus** (Barnes & McDunnough, 1918); valid (Crambus) 5415

**abnaki** (Klots, 1942); valid (Crambus) 5416

**dorsipunctellus** (Kearfott, 1908); valid (Crambus) 5417

geminatellus (Zeller, 1863); syn. (Crambus) 5417

**La** Bleszynski, 1966

**cerveza** B. Landry, 1995^148^; valid (La)

**Parapediasia** Bleszynski, 1966

Parapediasia Bleszynski, 1963^5^; nomen nudum

**hulstellus** (Fernald, 1885)^149^; valid (Crambus) 5386 403

**decorellus** (Zincken, 1821); valid (Chilo) 5450 426

polyactinellus (Zeller, 1863); syn. (Crambus) 5450 426

goodellianus (Grote, 1880); syn. (Crambus) 5450 426

bonusculalis (Hulst, 1886); syn. (Crambus) 5450 426

**teterrellus** (Zincken, 1821)^30^, ^134^; valid (Chilo) 5451 436

camurellus (Clemens, 1860); syn. (Crambus) 5451 436

**ligonellus** (Zeller, 1881)^150^; valid (Crambus) 430

**torquatella** B. Landry, 1995^151^; valid (Parapediasia)

**Almita** B. Landry, 1995^152^

**portalia** B. Landry, 1995; valid (Almita)

**texana** B. Landry, 1995; valid (Almita)

**Raphiptera** Hampson, 1896

**argillaceellus** (Packard, 1868); valid (Crambus) 5393 410

**a. minimellus** (Robinson, 1870); ssp. (Crambus) 5393

**Agriphila** Hübner, [1825]

Alisa Ganev & Hacker, 1984^5^

**biarmicus** (Tengström, 1865); valid extralimital (Crambus) 5395

pallidus (Strand, 1900)^5^; syn. (Crambus)

**a. illatella** (Fuchs, 1902)^5^; ssp. extralimital (Crambus)

**b. alpina** Bleszynski, 1957^5^; ssp. extralimital (Agriphila)

**c. paganellus** (McDunnough, 1925); ssp. (Crambus) 5395

**straminella** (Denis & Schiffermüller, 1775); valid (Tinea) 5396

marginellus (Stephens, 1834)^5^; syn. (Tinea)

subarcticellus (Strand, 1920)^5^;

syn. infrasubspecific (Crambus)

**plumbifimbriellus** (Dyar, 1904); valid (Crambus) 5397

**costalipartella** (Dyar, 1921); valid (Crambus) 5398

**ruricolellus** (Zeller, 1863); valid (Crambus) 5399

canadellus (Haimbach, 1930); syn. (Crambus) 5399

**undatus** (Grote, 1881); valid (Crambus) 5400

**anceps** (Grote, 1880); valid (Crambus) 5401 413

**biothanatalis** (Hulst, 1886); valid (Crambus) 5402 414

behrensellus (Fernald, 1887); syn. (Crambus) 5402 414

**vulgivagellus** (Clemens, 1860); valid (Crambus) 5403

aurifimbrialis (Walker, 1863); syn. (Crambus) 5403

chalybirostris (Zeller, 1863); syn. (Crambus) 5403

**attenuatus** (Grote, 1880); valid (Crambus) 5404

**Fernandocrambus** Aurivillius, 1922

Juania Aurivillius, 1922

**harpipterus** (Dyar, 1916); valid (Crambus) 5389 342

**ruptifascia** (Hampson, 1919; valid (Crambus) 5390 358

**Chrysoteuchia** Hübner, 1825

Amphibolia Snellen, 1884^153^; preocc. by Macquart, 1843

Veronese Bleszynski, 1962^154^

**topiarius** (Zeller, 1866); valid (Crambus) 5391

**a. vachellellus** (Kearfott, 1903); ssp. (Crambus) 5391

**Crambus** Fabricius, 1798

Palparia Haworth, 1811

Chilus Billberg, 1820

Tetrachila Hübner, 1822

Argyroteuchia Hübner, [1825]

**pascuella** (Linnaeus, 1758);

valid extralimital (PhalaenaTinea) 5339

scirpellus La Harpe, 1864^5^; syn. extralimital (Crambus)

fumipalpellus Mann, 1871^5^; syn. extralimital (Crambus)

acutulellus Chrétien, 1896^5^; syn. extralimital (Crambus)

collutellus Fuchs, 1902^5^; syn. extralimital (Crambus)

obscurellus Kuchlein, 1958^5^;

syn. extralimital, form (Crambus)

**a. extinctellus** Zeller, 1857^5^;

ssp. extralimital, in Staud., 1857 (Crambus)

**b. floridus** Zeller, 1872; ssp. (Crambus) 5339

**hamella** (Thunberg, 1794); valid (Tinea) 5340

ensigerella (Hübner, 1810-13)^5^; syn. (Tinea)

baccaestria (Haworth, 1811)^5^; syn. (Palparia)

hastiferellus Walker, 1863; syn. part, female (Crambus) 5340

**a. carpenterellus** Packard, 1874; ssp. (Crambus) 5340

**alienellus** (Germar & Kaulfuss, 1817);

valid extralimital (Chilo) 5341

zinckenellus (Sodoffsky, 1830)^5^; syn. (Chilo)

tigurinellus Duponchel, 1836^5^; syn. (Crambus)

ocellellus (Zetterstedt, 1839)^5^; syn. (Chilo)

hemnesensis Strand, 1919^5^;

syn. aberration infrasubspecific (Crambus)

moensis Strand, 1919^5^;

syn. aberration infrasubspecific (Crambus)

ranenensis Strand, 1919^5^;

syn. aberration infrasubspecific (Crambus)

**a. labradoriensis** Christoph, 1858; ssp. (Crambus) 5341

moestellus Walker, 1863; syn. (Crambus) 5341

**b. dissectus** Grote, 1880; ssp. (Crambus) 5341

**bidens** Zeller, 1872; valid (Crambus) 5342

**perlella** (Scopoli, 1763); valid (Phalaena) 5343

argentella (Fabricius, 1775)^5^; syn. (Tinea)

dealbella (Thunberg, 1788)^5^; syn. (Tinea)

arbustella (Fabricius, 1794)^5^; syn. (Tinea)

argyreus Stephens, 1834^5^; syn. (Crambus)

arbustorum Stephens, 1834^5^; syn. (Crambus)

warringtonellus Stainton, 1849^5^; syn. (Crambus)

pseudorostellus Müller-Rutz, 1923^155^; syn. (Crambus)

obscurellus Osthelder, 1939^5^;

syn. aberration infrasubspecific (Crambus)

**a. innotatellus** Walker, 1863; ssp. (Crambus) 5343

sericinellus Zeller, 1863; syn. (Crambus) 5343

inornatellus Clemens, 1864; syn. (Crambus) 5343

**b. aurellus** Zerny, 1914^5^; ssp. extralimital (Crambus)

**c. cupriacellus** Zerny, 1914^5^; ssp. extralimital (Crambus)

**d. flavonitellus** Zerny, 1935^5^; ssp. extralimital (Crambus)

auratus (D. Lucas, 1956)^5^;

syn. form, extralimital (Platytes)

**e. kirinellus** Bleszynski, 1965^5^;

ssp. extralimital (Crambus)

**f. monochromella** Herrich-Schäffer, 1852^156^;

ssp. extralimital (Crambus)

rostellus La Harpe, 1855^5^; syn. extralimital (Crambus)

**g. nigerrimus** Caradja, 1916^5^; ssp. extralimital (Crambus)

**h. pamiri** Bleszynski, 1959^5^; ssp. extralimital (Crambus)

**i. hachimantaiensis** Okano, 1957^5^;

ssp. extralimital (Crambus)

**unistriatellus** Packard, 1867; valid (Crambus) 5344

exesus Grote, 1880; syn. (Crambus) 5344

**whitmerellus** Klots, 1942; valid (Crambus) 5345

**a. browni** Klots, 1942; ssp. (Crambus) 5345

**tutillus** McDunnough, 1921; valid (Crambus) 5346

**awemellus** McDunnough, 1921; valid (Crambus) 5347

**lyonsellus** Haimbach, 1915; valid (Crambus) 5348

**youngellus** Kearfott, 1908; valid (Crambus) 5349

**daeckellus** Haimbach, 1907; valid (Crambus) 5350

**gausapalis** Hulst, 1886; valid (Crambus) 5351

**trichusalis** Hulst, 1886; valid (Crambus) 5352

**cockleellus** Kearfott, 1908; valid (Crambus) 5353

**ainslieellus** Klots, 1942; valid (Crambus) 5354

**praefectellus** (Zincken, 1821); valid (Chilo) 5355 372

involutellus Clemens, 1860; syn. (Crambus) 5355 372

**a. oslarellus** Haimbach, 1908; ssp. (Crambus) 5355

**bigelovi** Klots, 1967; valid (Crambus) 5356

**leachellus** (Zincken, 1818); valid (Chilo) 5357 373

pulchellus Zeller, 1863; syn. (Crambus) 5357 373

lativittellus Zeller, 1863; syn. (Crambus) 5357 373

hastiferellus Walker, 1863; syn. part male (Crambus) 5357 373

**cypridalis** Hulst, 1886; valid (Crambus) 5358 374

**occidentalis** Grote, 1880; valid (Crambus) 5359 375

agricolellus Dyar, 1923; syn. (Crambus) 5359 375

**rickseckerellus** Klots, 1940; valid (Crambus) 5360 376

**albellus** Clemens, 1860; valid (Crambus) 5361

**agitatellus** Clemens, 1860; valid (Crambus) 5362

alboclavellus Zeller, 1863; syn. (Crambus) 5362

carolinellus Haimbach, 1915; syn. (Crambus) 5362

**saltuellus** Zeller, 1863; valid (Crambus) 5363

**multilinellus** Fernald, 1887; valid (Crambus) 5364 377

**girardellus** Clemens, 1860; valid (Crambus) 5365

nivihumellus Walker, 1863; syn. (Crambus) 5365

**watsonellus** Klots, 1942; valid (Crambus) 5366 378

**sanfordellus** Klots, 1942; valid (Crambus) 5367 379

**braunellus** Klots, 1940; valid (Crambus) 5368 380

**quinquareatus** Zeller, 1877; valid (Crambus) 5369 381

extorralis Hulst, 1886; syn. (Crambus) 5369 381

argentictus Hampson, 1919; syn. (Crambus) 5369 381

**sperryellus** Klots, 1940; valid (Crambus) 5370

**leuconotus** Zeller, 1881; valid (Crambus) 5371 384

**satrapellus** (Zincken, 1821); valid (Chilo) 5372 386

elegantellus Walker, 1863; syn. (Crambus) 5372 386

aculeilellus Walker, 1863; syn. (Crambus) 5372 386

aureorufus Hampson, 1919; syn. (Crambus) 5372 386

**cyrilellus** Klots, 1942; valid (Crambus) 5373 390

**harrisi** Klots, 1967; valid (Crambus) 5374 391

**johnsoni** Klots, 1942; valid (Crambus) 5375 392

**sargentellus** Klots, 1942; valid (Crambus) 5376 393

**angustexon** Bleszynski, 1962; valid (Crambus) 5377 394

**laqueatellus** Clemens, 1860; valid (Crambus) 5378

semifusellus Walker, 1863; syn. (Crambus) 5378

**dimidiatellus** Grote, 1883^133^, ^157^; valid misplaced (Crambus) 5388

leucorhabdon Hampson, 1919; syn. (Crambus) 5388

**angulatus** Barnes & McDunnough, ^30^, ^133^, ^157^;

valid misplaced (Crambus) 5405 415

diegonellus Dyar, 1923; syn. (Crambus) 5405 415

**Schoenobiinae**

**Carectocultus** A. Blanchard, 1975^158^

**perstrialis** (Hübner, [1825]); valid (Agriphila) 5307 538

repugnatalis (Walker, 1863)^159^; syn. (Chilo) 5308 539

serriradiellus (Walker, 1863); syn. (Crambus) 5307 538

macrinellus (Zeller, 1866); syn. (Schoenobius) 5307 538

funerellus (Hampson, 1901); syn. (Chilo) 539

consortalis (Dyar, 1909); syn. (Argyria) 5308 539

**dominicki** A. Blanchard, 1975; valid (Carectocultus) 5309 537

**Leptosteges** Warren, 1889^160^

**xantholeucalis** (Guenée, 1854); valid (Parthenodes) 5300 552

fasciella (Fernald, 1887); syn. (Scirpophaga) 5300 552

**flavicostella** (Fernald, 1887); valid (Scirpophaga) 5301 544

**flavifascialis** (Barnes & McDunnough, 1913); valid (Patissa) 5302 545

**parthenialis** (Dyar, 1917); valid (Patissa) 5303 548

**chrysozona** (Dyar, 1917); valid (Patissa) 5304 543

**sordidalis** (Barnes & McDunnough, 1913); valid (Patissa) 5305 550

**vestaliella** (Zeller, 1872); valid (Scirpophaga) 5306 551

**Rupela** Walker, 1863

Storteria Barnes & McDunnough, 1913

**segrega** Heinrich, 1937; valid (Rupela) 5310 563

**tinctella** (Walker, 1863); valid (Salapola) 5311 566

zelleri (Möschler, 1882); syn. (Scirpophaga) 5311 566

holophaealis (Hampson, 1904); syn. (Scirpophaga) 5311 566

unicolor (Barnes & McDunnough, 1913); syn. (Storteria) 5311 566

**sejuncta** Heinrich, 1937; valid (Rupela) 5312 571

**Donacaula** Meyrick, 1890^161^

**sordidellus** (Zincken, 1821); valid (Chilo) 5313 597

**unipunctellus** (Robinson, 1870); valid (Schoenobius) 5314 599

**tripunctellus** (Robinson, 1870); valid (Schoenobius) 5315 598

**melinellus** (Clemens, 1860); valid (Chilo) 5316

**dispersellus** (Robinson, 1870); valid (Schoenobius) 5316

**albicostellus** (Fernald, 1888); valid (Schoenobius) 5316

uniformellus (Dyar, 1917); syn. (Schoenobius) 5316

**aquilellus** (Clemens, 1860); valid (Chilo) 5317

clemensellus (Robinson, 1870); syn. (Schoenobius) 5317

**pallulellus** (Barnes & McDunnough, 1912);

valid (Schoenobius) 5318 595

**longirostrellus** (Clemens, 1860); valid (Chilo) 5319

amblyptepennis (Dyar, 1917); syn. (Schoenobius) 5320

**roscidellus** (Dyar, 1917); valid (Schoenobius) 5321 596

bicolorellus (Hoffman, 1934); syn. (Schoenobius) 601

**nitidellus** (Dyar, 1917); valid (Schoenobius) 5322 594

**uxorialis** (Dyar, 1921); valid (Schoenobius) 5323 600

**maximellus** (Fernald, 1891); valid (Schoenobius) 5324 593

**Lathrotelinae**^162^

**Sufetula** Walker, 1859

Loetrina Walker, 1863

Mirobriga Walker, 1863

Pseudochoreutes Snellen, 1880

Nannomorpha Turner, 1908

Perforadix Sein, 1930^163^

**diminutalis** (Walker, [1866]); valid (Isopteryx) 5120 2933

dematrialis (Druce, 1896); syn. (Hydrocampa) 5120 2933

**carbonalis** Hayden, 2013^164^; valid (Sufetula)

**Acentropinae**^165^

**Oligostigmoides** Lange, 1956

**cryptalis** (Druce, 1896)^166^; valid (Cataclysta) 4766 1013

cryptale (Hampson, 1897);

syn. emendation (Oligostigma) 1013

**Langessa** Munroe, 1972

**nomophilalis** (Dyar, 1906); valid (Nymphula) 4758 1053

**Elophila** Hübner, 1822

Elophila Hübner, 1806^5^; nomen nudum

Hydrocampus Berthold, 1827

Hydrocampa Stephens, 1829

Hydrocampe Latreille, 1829^5^

Synclita Lederer, 1863^167^

Munroessa Lange, 1956^168^

Cyrtogramme Yoshiyasu, 1985

**ekthlipsis** (Grote, 1876)^169^; valid (Hydrocampa) 4747

**icciusalis** (Walker, 1859); valid (Leucochroma) 4748 1057

formosalis (Clemens, 1860); syn. (Hydrocampa) 4748 1057

genuialis (Lederer, 1863); syn. (Hydrocampa) 4748 1057

**a. albiplaga** (Munroe, 1972); ssp. (Munroessa) 4748

**b. avalona** (Munroe, 1972); ssp. (Munroessa) 4748

**faulalis** (Walker, 1859); valid (Leucochroma) 4749 1058

pacalis (Grote, 1881); syn. (Hydrocampa) 4749 1058

**nebulosalis** (Fernald, 1887); valid (Hydrocampa) 4750 1059

**gyralis** (Hulst, 1886); valid (Hydrocampa) 4751 1055

dentilinea (Hampson, 1897); syn. (Nymphula) 4751 1055

serralinealis (Barnes & Benjamin, 1924)^170^;

syn. (Nymphula) 4751 1055

**tinealis** (Munroe, 1972); valid (Synclita) 4754 1064

**obliteralis** (Walker, 1859); valid (Isopteryx) 4755 1061

proprialis (Fernald, 1888); syn. (Hydrocampa) 4755 1061

**atlantica** (Munroe, 1972); valid (Synclita) 4756

**occidentalis** (Lange, 1956); valid (Synclita) 4757 1063

**Contiger** Lange, 1956

**vittatalis** (Dyar, 1906); valid (Oligostigma) 4752 1067

**Parapoynx** Hübner, [1825]

Paraponyx Guenée, 1854^5^

Eustales Clemens, 1860; preocc. by Schoenherr, 1826

Sironia Clemens, 1860; preocc. by Hübner, 1823

Nymphaeella Grote, 1880

Hydreuretis Meyrick, 1885^171^

Microdracon Warren, 1890^172^

Cosmophylla Turner, 1908^171^

**maculalis** (Clemens, 1860); valid (Sironia) 4759 1076

seminivella (Walker, 1866); syn. (Nephopteryx) 4759 1076

dispar (Grote, 1880); syn. (Nymphaeella) 4759 1076

foeminalis (Dyar, 1906);

syn. aberration, infrasubspecific (Nymphula) 4759 1076

masculinalis (Dyar, 1906);

syn. aberration, infrasubspecific (Nymphula) 4759 1076

**obscuralis** (Grote, 1881); valid (Oligostigma) 4760 1078

**badiusalis** (Walker, 1859); valid (Cymoriza) 4761 1079

albalis (Robinson, 1869); syn. (Oligostigma) 4761 1079

**curviferalis** (Walker, [1866]); valid (Oligostigma) 4762 1080

**seminealis** (Walker, 1859); valid (Oligostigma) 4763 1081

tedyuscongalis (Clemens, 1860); syn. (Eustales) 4763 1081

**allionealis** Walker, 1859; valid (Paraponyx) 4764 1082

itealis (Walker, 1859)^173^; syn. (Hydrocampa) 4764 1082

aptalis Lederer, 1863; syn. (Parapoynx) 4764 1082

cretacealis Lederer, 1863; syn. (Parapoynx) 4764 1082

plenilinealis Grote, 1881; syn. (Paraponyx) 4764 1082

**diminutalis** Snellen, 1880; valid (Parapoynx) 4765 1091

dicentra Meyrick, 1885^174^; syn. (Paraponyx)

pallida (Butler, 1886)^5^; syn. (Oligostigma)

uxorialis (Strand, 1919)^175^; syn. (Nymphula)

**Neocataclysta** Lange, 1956

**magnificalis** (Hübner, [1796]); valid (Pyralis) 4743 1096

lamialis (Walker, 1859); syn. (Cataclysta) 4743 1096

helopalis (Clemens, 1860); syn. (Cataclysta?) 4743 1096

**Chrysendeton** Grote, 1881

**medicinalis** (Grote, 1881); valid (Cataclysta) 4744 1104

**kimballi** Lange, 1956; valid (Chrysendeton) 4745 1105

**imitabilis** (Dyar, 1917); valid (Elophila) 4746 1106

**nigrescens** Heppner, 1991^176^; valid (Chrysendeton)

**Nymphuliella** Lange, 1956

**daeckealis** (Haimbach, 1915); valid (Diathrausta) 4753

broweri (Heinrich, 1940); syn. (Nymphula) 4753

**Argyractis** Hampson, 1897

**drumalis** (Dyar, 1906)^177^; valid (Elophila) 4770 1129

**Neargyractis** Lange, 1956

**slossonalis** (Dyar, 1906); valid (Elophila) 4769 1144

**Usingeriessa** Lange, 1956

**onyxalis** (Hampson, 1897); valid (Cataclysta) 4767 1151

cancellalis (Dyar, 1917); syn. (Elophila) 4767 1151

**brunnildalis** (Dyar, 1906); valid (Elophila) 4768 1150

**Petrophila** Guilding, 1830

Parargyractis Lange, 1956

**daemonalis** (Dyar, 1907); valid (Elophila) 4771 1162

**cappsi** (Lange, 1956); valid (Parargyractis) 4772 1194

**kearfottalis** (Barnes & McDunnough, 1917);

valid (Cataclysta) 4773 1193

kearfottalis (Dyar, 1906);

syn. infrasubspecific, nomen nudum (Elophila) 1193

**bifascialis** (Robinson, 1869); valid (Cataclysta) 4774 1195

**jaliscalis** (Schaus, 1906); valid (Cataclysta) 4775 1231

satanalis (Dyar, 1917); syn. (Elophila) 4775 1231

**hodgesi** (Munroe, 1972); valid (Parargyractis) 4776

**fulicalis** (Clemens, 1860); valid (Cataclysta) 4777 1228

angulatalis (Lederer, 1863); syn. (Cataclysta) 4777 1228

**santafealis** (Heppner, 1976); valid (Parargyractis) 4778 1229

**canadensis** (Munroe, 1972); valid (Parargyractis) 4779

**confusalis** (Walker, [1866]); valid (Cataclysta) 4780

truckeealis (Dyar, 1917); syn. (Elophila) 4780

**avernalis** (Grote, 1878); valid (Chrysendeton) 4781 1225

confusalis (Barnes & McDunnough, 1913);

syn. preocc. by Walker, [1866] (Argyractis) 4781 1225

**cronialis** (Druce, 1896); valid (Cataclysta) 4782 1223

**longipennis** (Hampson, 1906); valid (Argyractis) 4783 1224

**schaefferalis** (Dyar, 1906); valid (Elophila) 4784 1226

castusalis (Schaus, 1924); syn. (Argyractis) 4784 1226

**heppneri** A. Blanchard & Knudson, 1983^178^;

valid (Petrophila) 1230

**Eoparargyractis** Lange, 1956

Eoparargyractis Lange, 1956; nomen nudum

**irroratalis** (Dyar, 1917); valid (Elophila) 4785 1251

**floridalis** Lange, 1956; valid (Eoparargyractis) 4786 1250

**plevie** (Dyar, 1917); valid (Elophila) 4787 1249

**Oxyelophila** Forbes, 1922

**callista** Forbes, 1922; valid (Argyractis (Oxyelophila)) 4788 1252

**Acentria** Stephens, 1829^179^

Setina Hübner, 1819^5^; preocc. by Schrank, 1802

Zancle Stephens, 1833

Acentropus Curtis, 1834

**ephemerella** (Denis & Schiffermüller, 1775);

valid introduced (Tinea)

nivea (Olivier, 1791); syn. (Phryganea) 5299

sembris (Hübner, 1809); syn. (Bombyx)

phryganea (Hübner, 1809); syn. (Bombyx)

ephemera (Hübner, 1819); syn. (Setina)

hansoni (Stephens, 1833); syn. (Zancle) 5299

garnonsii (Curtis, 1834); syn. (Acentropus) 5299

nivosa (Stephens, 1834)^5^; syn. (Phryganea)

newae (Kolenati, 1858); syn. (Acentropus)

latipennis (Möschler, 1860); syn. (Acentropus)

badensis (Nolcken, 1869); syn. (Acentropus)

germanicus (Nolcken, 1869); syn. (Acentropus)

**a. obscurus** (Tengström, 1869); ssp. extralimital (Acentropus)

**Pyralidae**

**Chrysauginae**

**Caphys** Walker, 1863

Ugra Walker, 1863

Euexippe Ragonot, 1891

**arizonensis** Munroe, 1970; valid (Caphys) 5537 3267

**Parachma** Walker, [1866]

Zazaca Walker, [1866]

Perseis Ragonot, 1891; preocc. by Gistl, 1848

Perseistis Strand, 1921; repl. name

Artopsis Dyar, 1908^180^

**ochracealis** Walker, [1866]; valid (Parachma) 5538 3457

auratalis (Walker, [1866]); syn. (Zazaca) 5538 3457

culiculalis (Hulst, 1886); syn. (Asopia) 5538 3457

borregalis (Dyar, 1909)^181^; syn. (Artopsis) 5539 3452

nua (Dyar, 1914); syn. (Artopsis) 5538 3457

**Basacallis** Cashatt, 1984^182^

**tarachodes** (Dyar, 1914); valid (Parachma) 5540 3251

**Acallis** Ragonot, 1891

Polloccia Dyar, 1910^183^

**gripalis** (Hulst, 1886); valid (Aglossa) 5541 3208

fernaldi Ragonot, 1891; syn. (Acallis) 5541 3208

angustipennis (Warren, 1891); syn. (Ugra) 5541 3208

centralis Dyar, 1910^184^; syn. (Acallis) 5542 3207

**alticolalis** (Dyar, 1910)^185^; valid (Polloccia) 5543 3206

**Zaboba** Dyar, 1914

**mitchelli** (Dyar, 1914)^186^; valid (Acallis) 5544 3579

**unicoloralis** Munroe, 1970; valid (Zaboba) 5545 3581

**Anemosella** Dyar, 1914

Balidarcha Dyar, 1914^187^

**basalis** Dyar, 1914; valid (Anemosella) 3215

polingalis Barnes & Benjamin, 1926^188^; syn. (Anemosella) 5548 3218

**nevalis** (Barnes & Benjamin, 1925); valid (Lepidomys) 5546 3216

**obliquata** (Henry Edwards, 1886); valid (Earias) 5547 3217

albistrigalis (Barnes & McDunnough, 1913);

syn. (Chalinitis) 5547 3217

**viridalis** (Barnes & McDunnough, 1912); valid (Chalinitis) 5549 3219

cuis (Dyar, 1914)^189^; syn. (Balidarcha) 5549 3219

**Lepidomys** Guenée, 1852

Chalinitis Ragonot, 1891

**irrenosa** Guenée, 1852; valid (Lepidomys) 5550 3400

olealis (Ragonot, 1891); syn. (Chalinitis) 5550 3400

**Epitamyra** Ragonot, 1891

**albomaculalis** (Möschler, 1890)^190^; valid (Tamyra) 3336

**Paragalasa** Cashatt, 2013^191^

**exospinalis** Cashatt, 2013; valid (Paragalasa)

**Negalasa** Barnes & McDunnough, 1913

**fumalis** Barnes & McDunnough; 1913; valid (Negalasa) 5551 3436

**rubralis** Barnes & McDunnough, 1913^192^; spp. (Negalasa) 5551 3437

**Galasa** Walker, 1866

Cordylopeza Zeller, 1873

**nigrinodis** (Zeller, 1873); valid (Cordylopeza) 5552 3352

rubrana Fitch, 1891;

nomen nudum published in syn. (Galasa) 5552 3352

palmipes Grote & Robinson, 1891;

nomen nudum published in syn. (Galasa) 5552 3352

**nigripunctalis** (Barnes & McDunnough, 1913);

valid (Cordylopeza) 5553 3353

fulvusana Haimbach, 1915; syn. (Galasa) 5553 3353

**Penthesilea** Ragonot, 1891

**difficilis** (Felder, Felder & Rogenhofer, 1875); valid (Amblyura) 3475

leucogramma (Hampson, 1904)^193^; syn. (Tetraschistis) 5554 3475

**sacculalis** Ragonot, 1891; valid (Penthesilea) 5555 3476

**a. baboquivariensis** Cashatt, 2013^194^; ssp. (Penthesilea)

**Tosale** Walker, 1863

Fabatana Walker, [1866]

Siparocera Grote, 1875

Callocera Grote, 1875; unavailable (ICZN Art. 11d)

Siparocera Robinson, 1876; preocc. by Grote, 1875

Restidia Dyar, 1914

**oviplagalis** (Walker, [1866]); valid (Fabatana) 5556 3552

anthoecioides (Grote & Robinson, 1867); syn. (Asopia) 5556 3552

nobilis (Grote, 1875); syn. (Siparocera) 3552

nobilis (Robinson, 1876); syn. redescription (Siparocera) 5556 3552

**similalis** Barnes & Benjamin, 1924; valid (Tosale) 5557 3553

**aucta** Hampson, 1892; valid (Tosale) 5558 3544

**Salobrena** Walker, 1863

Oectoperia Zeller, [1876]

Ballonicha Möschler, 1886^195^

Teucronoma Meyrick, 1936^195^

**sincera** (Zeller, [1876]); valid (Oectoperia) 5559 3499

**recurvata** (Möschler, 1886); valid (Ballonicha) 3497

rubiginea (Hampson, 1897)^196^; syn. (Abaera) 5560 3497

**vacuana** (Walker, 1863); valid (Rucuma) 3501

birectalis (Hampson, 1897)^197^; syn. (Epitamyra) 5561 3501

**Satole** Dyar, 1908

**ligniperdalis** Dyar, 1908; valid (Satole) 5562 3508

**Clydonopteron** Riley, 1880^198^

**sacculana** (Bosc, [1800]); valid (Pyralis) 3306

tecomae Riley, 1880^199^; syn. (Clydonopteron) 5563 3306

**Bonchis** Walker, 1862

Ethnistis Lederer, 1863

Gazaca Walker, [1866]

Vurna Walker, [1866]

Zarania Walker, [1866]

**munitalis** (Lederer, 1863); valid (Ethnistis) 5564 3259

instructalis (Walker, [1866]); syn. (Vurna) 5564 3259

cossalis (Walker, [1866]); syn. (Zarania) 5564 3259

dirutalis (Walker, [1866]); syn. (Gazaca) 5564 3259

**Streptopalpia** Hampson, 1895

**minusculalis** (Möschler, 1890); valid (Tamyra) 5565 3524

deera (Druce, 1895); syn. (Galasa) 5565 3524

ustalis Hampson, 1895; syn. (Streptopalpia) 5565 3524

**Arta** Grote, 1875

Xantippides Dyar, [1908]^200^

**statalis** Grote, 1875; valid (Arta) 5566 3237

**epicoenalis** Ragonot, 1891; valid (Arta) 5567 3231

descansalis (Dyar, [1908])^201^; syn. (Xantippides) 5567 3231

beatifica (Dyar, 1921)^202^; syn. (Xantippe) 5569 3564

**brevivalvalis** Cashatt, 2013^203^; valid (Arta)

**olivalis** Grote, 1878; valid (Arta) 5568 3234

**Condylolomia** Grote, 1873

**participialis** Grote, 1873; valid (Condylolomia) 5571 3309

**Heliades** Ragonot, 1891

**mulleolella** (Hulst, 1887); valid (Pempelia) 5574 3372

uranides (Dyar, 1921)^204^; syn. (Xantippe) 5570 3578

**lindae** Cashatt, 2013^205^; valid (Heliades)

**huachucalis** (Haimbach, 1915)^206^; valid (Pyrausta) 5574 3372

**Galleriinae**

**Galleria** Fabricius, 1798

Adeona Rafinesque, 1815^5^

Cerioclepta Sodoffsky, 1837

Vindana Walker, 1866

**mellonella** (Linnaeus, 1758); valid (PhalaenaTinea) 5622 3584

cereana (Blom, 1764); syn. (Phalaena) 5622 3584

cerella (Fabricius, 1775); syn. emendation (Tinea) 5622 3584

cerea (Haworth, 1811); syn. emendation (Tinea) 5622 3584

cerealis Hübner, 1825; syn. emendation (Galleria) 5622 3584

obliquella (Walker, 1866); syn. (Vindana) 5622 3584

austrinia Felder, Felder & Rogenhofer, 1875;

syn. (Galleria) 5622 3584

crombrugghella Dufrane, 1930;

syn. infrasubspecific, aberration (Galleria) 5622 3584

**Achroia** Hübner, [1819]

Meliphora Guenée, 1845

Vobrix Walker, 1864

**grisella** (Fabricius, 1794); valid nomen protectum (Tinea) 5623 3585

cinerana (Fabricius, 1781); syn. supressed name (Pyralis?) 3585

aluearia (Fabricius, 1798); syn. (Galleria) 5623 3585

cinereola (Hübner, 1802); syn. (Bombyx) 5623 3585

alvea (Haworth, 1811); syn. emendation (Galleria) 5623 3585

alvearia (Stephens, 1829)^5^; syn. unavailable name (Galleria)

alveariella (Guenée, 1845); syn. emendation (Meliphora) 5623 3585

anticella (Walker, 1863); syn. (Tinea) 5623 3585

obscurevittella Ragonot, 1901; syn. (Achroia) 5623 3585

major Dufrane, 1930;

syn. infrasubspecific, aberration (Achroia) 5623 3585

ifranella Lucas, 1956;

syn. infrasubspecific, variety (Achroia) 3585

**Trachylepidia** Ragonot, 1887

Aganactesis Dyar, 1921

**fructicassiella** Ragonot, 1887; valid (Trachylepidia) 5624 3586

indecora (Dyar, 1921); syn. (Aganactesis) 5624 3586

**Omphalocera** Lederer, 1863

**cariosa** Lederer, 1863; valid (Omphalocera) 5625 3587

dentosa Grote, 1881; syn. (Omphalocera) 5625 3587

**occidentalis** Barnes & Benjamin, 1924; valid (Omphalocera) 5626 3589

**munroei** Martin, 1956; valid emendation (Omphalocera) 5627 3588

**Thyridopyralis** Dyar, 1901

**gallaerandialis** Dyar, 1901; valid (Thyridopyralis) 5628 3590

**Aphomia** Hübner, [1825]

Ilithyia Berthold, 1827

Melia Curtis, 1828; preocc. Bosc, 1813

Melissoblaptes Zeller, 1839

Aphomoea Agassiz, [1847]; emendation

Arenipses Hampson, 1901^207^

**sociella** (Linnaeus, 1758); valid (PhalaenaTinea) 5629

colonella (Linnaeus, 1758); syn. (Tinea) 5629

tribunella (Denis & Schiffermüller, 1775); syn. (Tinea) 5629

socia (Fabricius, 1798); syn. emendation (Lithosia) 5629

colonum (Fabricius, 1798); syn. emendation (Crambus) 5629

colonatus (Haworth, 1809); syn. emendation (Crambus) 5629

rufinella Krulikowsky, 1909;

syn. infrasubspecific (Aphomia) 5629

grisea Turati, 1913^5^; syn. (Aphomia)

asiatica Caradja, 1916^5^; syn. variety (Aphomia)

virescens Skala, 1929; syn. infrasubspecific (Aphomia) 5629

minor Dufrane, 1930; syn. infrasubspecific (Aphomia) 5629

lanceolata Dufrane, 1930; syn. infrasubspecific (Aphomia) 5629

eritrella Della Beffa, 1941^5^; syn. variety (Aphomia)

pedemontella Della Beffa, 1941^5^; syn. variety (Aphomia)

**terrenella** Zeller, 1848; valid (Aphomia) 5630

furellus (Zeller, 1873); syn. (Melissoblaptes) 5630

decorella (Hulst, 1892)^208^; syn. (Paralipsa) 5633

**fulminalis** (Zeller, 1872)^209^; valid (Melissoblaptes) 3592

**gularis** (Zeller, 1877)^210^; valid (Melissoblaptes) 5632

modesta (Butler, 1879); syn. (Paralipsa) 5632

tenebrosus (Butler, 1879); syn. (Melissoblaptes) 5632

**cephalonica** (Stainton, 1866)^210^; valid (Melissoblaptes) 5634 3594

oeconomellus (Mann, 1872); syn. (Melissoblaptes) 5634 3594

translineella (Ragonot, 1901); syn. (Corcyra) 5634 3594

theobromae (Dyar, 1913); syn. (Tineopsis) 5634 3594

lineata (Legrand, 1966); syn. (Anerastia) 3594

**Epimorius** Zeller, 1877

Athaliptis Schaus, 1913^211^

**testaceellus** Ragonot, 1887^212^; valid (Epimorius) 3598

**Stenopaschia** Hampson, 1906

Stenopaschia Dyar, 1914^5^; preocc. by Hampson, 1906

Tapinolopha Dyar, 1918^5^

**trichopteris** Dyar, 1914^213^; valid (Stenopaschia) 3611

variegata (Dyar, 1918); syn. (Tapinolopha) 3611

**Cacotherapia** Dyar, 1904^214^

Macrotheca Ragonot, 1891; preocc. by Waagen, 1880

**interalbicalis** (Ragonot, 1891); valid (Macrotheca) 5635 3621

vulnifera (Dyar, 1917); syn. (Macrotheca) 5635 3621

**bilinealis** (Barnes & McDunnough, 1918);

valid (Macrotheca) **comb. n.** 5636 3618

**angulalis** (Barnes & McDunnough, 1918);

valid (Macrotheca) **comb. n.** 5637 3617

**unicoloralis** (Barnes & McDunnough, 1913);

valid (Macrotheca) 5638 3627

**unipuncta** (Dyar, 1913); valid (Macrotheca) 5639 3628

**ponda** Dyar, 1907; valid (Cacotherapia) 5640 3626

**nigrocinereella** (Hulst, 1900); valid (Aurora) 5641 3624

**flexilinealis** Dyar, 1905; valid (Cacotherapia) 5642 3620

**leucocope** (Dyar, 1917); valid (Macrotheca) 5643 3623

**lecerfialis** (Barnes & Benjamin, 1925); valid (Macrotheca) 5644 3622

**Alpheias** Ragonot, 1891

Amestria Ragonot, 1891

**vicarilis** Dyar, 1913; valid (Alpheias) 5645 3636

**transferens** Dyar, 1913; valid (Alpheias) 5646 3635

**querula** Dyar, 1913; valid (Alpheias) 5647 3634

**oculiferalis** (Ragonot, 1891); valid (Amestria) 5648 3633

**Alpheioides** Barnes & McDunnough, 1912

**parvulalis** Barnes & McDunnough, 1912;

valid (Alpheioides) 5649 3637

**Decaturia** Barnes & McDunnough, 1912

**pectinalis** Barnes & McDunnough, 1912;

valid (Decaturia) 5650 3638

**Phycitinae**

**Acrobasis** Zeller, 1839

Conobathra Meyrick, 1886^215^

Mineola Hulst, 1890

Seneca Hulst, 1890

Numonia Ragonot, 1893^216^

Trachycera Ragonot, 1893^215^

Acrocaula Hulst, 1900

Hylopylora Meyrick, 1933^216^

Rhodophaeopsis Amsel, 1950^216^

Catacrobasis Gozmány, 1958

Cyprusia Amsel, 1958^5^

Cyphita Roesler, 1971^217^

**indigenella** (Zeller, 1848); valid (Myelois) 5651

nebulo (Walsh, 1860); syn. (Phycita) 5651

nebulella Riley, 1872; syn. (Phycita (Acrobasis)) 5651

zelatella (Hulst, 1887); syn. (Myelois) 5651

grossbecki (Barnes & McDunnough, 1917)^218^;

syn. (Mineola) 5652

**vaccinii** Riley, 1884; valid (Acrobasis) 5653

**amplexella** Ragonot, 1887; valid (Acrobasis) 5654

**tricolorella** Grote, 1878; valid (Acrobasis) 5655 3922

scitulella (Hulst, 1900); syn. (Mineola) 5655 3922

**comptella** Ragonot, 1887; valid (Acrobasis) 5656 3923

kofa (Opler, 1977)^219^; syn. (Rhodophaea) 5697 3923

fria (Opler, 1977)^219^; syn. (Rhodophaea) 5699 3923

neva (Opler, 1977)^219^; syn. (Rhodophaea) 5700 3923

**minimella** Ragonot, 1889; valid (Acrobasis) 5657

nigrosignella Hulst, 1890; syn. (Acrobasis) 5657

**palliolella** Ragonot, 1887; valid (Acrobasis) 5659

albocapitella Hulst, 1888; syn. (Acrobasis) 5659

feltella Dyar, 1910^220^; syn. (Acrobasis) 5658

**caryalbella** Ely, 1913; valid (Acrobasis) 5660

**juglandis** (LeBaron, 1872); valid (Phycita) 5661 3925

**sylviella** Ely, 1908; valid (Acrobasis) 5662

**kylesi** Neunzig, 1986^221^; valid (Acrobasis)

**tumidulella** (Ragonot, 1887)^222^; syn. (Cateremna) 5693

kearfottella Dyar, 1905; syn. (Acrobasis) 5663

**caryae** Grote, 1881; valid (Acrobasis) 5664

**carpinivorella** Neunzig, 1970; valid (Acrobasis) 5665

**elyi** Neunzig, 1970; valid (Acrobasis) 5666

**texana** Neunzig, 1986^221^; valid (Acrobasis)

**nuxvorella** Neunzig, 1970; valid (Acrobasis) 5667

**juglanivorella** Neunzig, 1986^221^; valid (Acrobasis)

**caulivorella** Neunzig, 1986^221^; valid (Acrobasis)

**evanescentella** Dyar, 1908; valid (Acrobasis) 5668

**stigmella** Dyar, 1908; valid (Acrobasis) 5669

**aurorella** Ely, 1910; valid (Acrobasis) 5670

**exsulella** (Zeller, 1848); valid (Myelois) 5672

septentrionella Dyar, 1925; syn. (Acrobasis) 5672

peplifera Dyar, 1925^223^; syn. (Acrobasis) 5671

**angusella** Grote, 1880; valid (Acrobasis) 5673

eliella Dyar, 1908; syn. (Acrobasis) 5673

**demotella** Grote, 1881; valid (Acrobasis) 5674

**latifasciella** Dyar, 1908; valid (Acrobasis) 5675

**irrubriella** Ely, 1908; valid (Acrobasis) 5676

**normella** Dyar, 1908; valid (Acrobasis) 5677

malipennella Dyar, 1908^224^; syn. (Acrobasis) 5678

secundella Ely, 1913^224^; syn. (Acrobasis) 5681

**ostryella** Ely, 1913; valid (Acrobasis) 5680

**coryliella** Dyar, 1908; valid (Acrobasis) 5682

**cirroferella** Hulst, 1892; valid (Acrobasis) 5684 3926

myricella Barnes & McDunnough, 1917^225^;

syn. (Acrobasis) 5692 3926

**cunulae** Dyar & Heinrich, 1929; valid (Acrobasis) 5685

**caryivorella** Ragonot, 1887; valid (Acrobasis) 5686

comacornella (Hulst, 1900)^226^; syn. (Acrocaula) 5687

**betulella** Hulst, 1890; valid (Acrobasis) 5688

hebescella Hulst, 1890^227^; syn. (Acrobasis) 5683

**betulivorella** Neunzig, 1975; valid (Acrobasis) 5689

**rubrifasciella** Packard, 1874; valid (Acrobasis) 5690

dyarella Ely, 1910^228^; syn. (Acrobasis) 5679

alnella McDunnough, 1922; syn. (Acrobasis) 5690

**comptoniella** Hulst, 1890; valid (Acrobasis) 5691

**blanchardorum** Neunzig, 1973; valid (Acrobasis) 5694 3924

**caliginella** (Hulst, 1887)^229^; valid (Nephopteryx) 5695 3929

caliginoidella (Dyar, 1905); syn. (Myelois) 5695 3929

cruza (Opler, 1977)^230^; syn. (Rhodophaea) 5696 3929

durata (Opler, 1977)^230^; syn. (Rhodophaea) 5698 3929

yuba (Opler, 1977)^230^; syn. (Rhodophaea) 5701 3929

**suavella** (Zincken, 1818)^229^; valid (Phycis) 5702

porphyrea (Stephens, 1834); syn. (Phycita) 5702

supposita (Heinrich, 1940); syn. (Mineola) 5702

**pallicornella** (Ragonot, 1887); valid (Rhodophaea) 5703 3930

**Anabasis** Heinrich, 1956

**ochrodesma** (Zeller, 1881); valid (Myelois) 5704 3931

crassisquamella (Hampson, 1901); syn. (Acrobasis) 5704 3931

**Hypsipyla** Ragonot, 1888

**grandella** (Zeller, 1848); valid (Nephopteryx) 5705 3938

cnabella Dyar, 1914; syn. (Hypsipyla) 5705 3938

**Anypsipyla** Dyar, 1914

**univitella** Dyar, 1914^231^; valid (Anypsipyla) 3964

**Crocidomera** Zeller, 1848

**imitata** Neunzig, 1990^232^; valid (Crocidomera)

**Cuniberta** Heinrich, 1956

**subtinctella** (Ragonot, 1887); valid (Nephopteryx) 5707

**Adanarsa** Heinrich, 1956

**intransitella** (Dyar, 1905); valid (Rhodophaea) 5708

**Bertelia** Barnes & McDunnough, 1913

**grisella** Barnes & McDunnough, 1913; valid (Bertelia) 5709 3954

**dupla** A. Blanchard, 1976; valid (Bertelia) 5710 3955

**Hypargyria** Ragonot, 1888

**slossonella** (Hulst, 1900); valid (Salebria) 5711 3957

tenuella (Barnes & McDunnough, 1913);

syn. (Acrobasis) 5711 3957

**Chararica** Heinrich, 1956

**annuliferella** (Dyar, 1905); valid (Myelois) 5712 3959

**hystriculella** (Hulst, 1887); valid (Acrobasis) 5713 3958

**bicolorella** (Barnes & McDunnough, 1917);

valid (Rhodophaea) 5714

**Myelopsis** Heinrich, 1956

**immundella** (Hulst, 1890); valid (Myelois) 5717 3961

**subtetricella** (Ragonot, 1889); valid (Myelois) 5718

zonulella (Ragonot, 1889); syn. (Myelois) 5718

obnupsella (Hulst, 1890); syn. (Myelois) 5718

**minutularia** (Hulst, 1887)^233^; valid (Dioryctria) 5719 3960

coniella (Ragonot, 1887)^234^; syn. (Myelois) 5716 3960

nefas (Dyar, 1922)^234^; syn. (Rampylla) 5716 3960

**alatella** (Hulst, 1887); valid (Acrobasis) 5720 3962

rectistrigella (Ragonot, 1887); syn. (Myelois) 5720 3962

fragilella (Dyar, 1904); syn. (Myelois) 5720 3962

piazzella (Dyar, 1925); syn. (Myelois) 5720 3962

**Myelopsoides** Neunzig, 1998^235^

**venustus** Neunzig, 1998; valid (Myelopsoides)

**Apomyelois** Heinrich, 1956

Ectomyelois Heinrich, 1956^236^

Spectrobates Roesler, 1956 (not Meyrick, 1935)

**bistriatella** (Hulst, 1887); valid (Dioryctria) 5721

bilineatella (Ragonot, 1887); syn. (Myelois) 5721

subcognata (Ragonot, 1887)^5^; syn. (Myelois)

neophanes (Durrant, 1915)^5^; syn. (Myelois)

**ceratoniae** (Zeller, 1839); valid (Myelois) 5723 3966

ceratoniella (F. v. Röslerstamm, 1839); syn. (Phycis) 5723 3966

pryerella (Vaughan, 1870); syn. (Trachonitis) 5723 3966

tuerkheimiella (Sorhagen, 1881); syn. (Myelois) 5723 3966

zellerella (Sorhagen, 1881); syn. (Euzophera) 5723 3966

dentilinella (Hampson, 1896); syn. (Phycita) 3966

psarella (Hampson, 1903); syn. (Hypsipyla) 5723 3966

rivulalis (Warren & Rothschild, 1905);

syn. (Heterographis) 3966

oporedestella (Dyar, 1911); syn. (Myelois) 5723 3966

phoenicis (Durrant, 1915); syn. (Myelois) 5723 3966

durandi (Lucas, 1950); syn. (Laodamia) 3966

**Amyelois** Amsel, 1956

Paramyelois Heinrich, 1956

**transitella** (Walker, 1863); valid (Nephopteryx) 5724 3971

notatalis (Walker, 1863); syn. (Nephopteryx) 5724 3971

solitella (Zeller, 1881); syn. (Myelois) 5724 3971

duplipunctella (Ragonot, 1887); syn. (Myelois) 5724 3971

venipars (Dyar, 1914); syn. (Myelois) 5724 3971

cassiae (Dyar, 1917); syn. (Emporia) 5724 3971

**Fundella** Zeller, 1848

Ballovia Dyar, 1913

**pellucens** Zeller, 1848; valid (Fundella) 5725 4013

cistipennis (Dyar, 1913); syn. (Ballovia) 5725 4013

**argentina** Dyar, 1919; valid (Fundella) 5726 4014

eucasis Dyar, 1919; syn. (Fundella) 5726 4014

agapella Schaus, 1923^237^; syn. (Fundella)

**ignobilis** Heinrich, 1956^238^; valid (Fundella) 4016

**Promylea** Ragonot, 1887

**lunigerella** Ragonot, 1887; valid (Promylea) 5727

**a. glendella** (Dyar, 1906); ssp. (Myelois) 5727

**Anadelosemia** Dyar, 1919

**texanella** (Hulst, 1892); valid (Myelois) 5728 4035

dulciella (Hulst, 1900); syn. (Myelois) 5728 4035

**condigna** Heinrich, 1956; valid (Anadelosemia) 5729

**Dasypyga** Ragonot, 1887

**alternosquamella** Ragonot, 1887; valid (Dasypyga) 5730 4036

stictophorella Ragonot, 1887; syn. (Dasypyga) 5730 4036

**salmocolor** A. Blanchard, 1970; valid (Dasypyga) 5731 4037

**Scorylus** Heinrich, 1956

**cubensis** Heinrich, 1956^239^; valid (Scorylus) 4045

**Davara** Walker, 1859

Homalopalpia Dyar, 1914

Eucardinia Dyar, 1918

**caricae** (Dyar, 1913); valid (Ulophora) 5732 4046

dalera (Dyar, 1914); syn. (Homalopalpia) 5732 4046

**Sarasota** Hulst, 1900

Cuba Dyar, 1919

**plumigerella** Hulst, 1900; valid (Sarasota) 5733

**Atheloca** Heinrich, 1956

**subrufella** (Hulst, 1887); valid (Nephopteryx) 5734 4069

filiolella (Hulst, 1888); syn. (Nephopteryx) 5734 4069

ptychis (Dyar, 1919); syn. (Hyalospila) 5734 4069

**Triozosneura** A. Blanchard, 1973

**dorsonotata** A. Blanchard, 1973; valid (Triozosneura) 5735 4082

**Monoptilota** Hulst, 1900

**pergratialis** (Hulst, 1886); valid (Nephopteryx) 5736 4092

grotella (Ragonot, 1887); syn. (Nephopteryx) 5736 4092

nubilella Hulst, 1900; syn. (Monoptilota) 5736 4092

**Zamagiria** Dyar, 1914

Anegcephalesis Dyar, 1917^240^

**australella** (Hulst, 1900); valid (Selagia) 5737

bumeliella (Barnes & McDunnough, 1913);

syn. (Immyrla) 5737

**laidion** (Zeller, 1881); valid (Myelois) 5739 4099

deia Dyar, 1919; syn. (Zamagiria) 5739 4099

striella Dyar, 1919; syn. (Zamagiria) 5739 4099

**arctella** (Ragonot, 1887); valid (Phycita) 5740 4103

cathaeretes (Dyar, 1917); syn. (Anegcephalesis) 5740 4103

**Philocrotona** Neunzig, 2003^241^

**kendalli** (A. Blanchard, 1970); valid (Zamagiria) 5738 4102

**Ancylostomia** Ragonot, 1893

**stercorea** (Zeller, 1848); valid (Myelois) 5741 4105

ignobilis (Butler, 1878); syn. (Anerastia) 5741 4105

diffissella (Zeller, 1881); syn. (Pempelia) 5741 4105

**Caristanius** Heinrich, 1956

**decoloralis** (Walker, 1863); valid (Trachonitis) 5742 4110

metagrammalis (Walker, 1863); syn. (Nephopteryx) 5742 4110

furfurella (Hulst, 1887); syn. (Nephopteryx) 5742 4110

floridellus (Hulst, 1890); syn. (Elasmopalpus) 5742 4110

**minimus** Neunzig, 1977; valid (Caristanius) 5743 4111

**Etiella** Zeller, 1839

Rhamphodes Guenée, 1845

Mella Walker, 1859

Alata Walker, 1863; preocc. by Linck, 1783

Arucha Walker, 1863

Modiana Walker, 1863

Ceratamma Butler, 1881

**zinckenella** (Treitschke, 1832); valid (Phycis) 5744 4113

etiella (Treitschke, 1835); syn. repl. name (Phycis) 5744 4113

colonnellus (Costa, [1836]); syn. (Chilo) 5744 4113

majorellus (Costa, [1836]); syn. (Chilo) 5744 4113

dymnusalis (Walker, 1859); syn. (Mella) 5744 4113

heraldella (Guenée, 1862); syn. (Rhamphodes) 5744 4113

anticalis (Walker, 1863); syn. (Alata) 5744 4113

indicatalis (Walker, 1863); syn. (Arucha) 5744 4113

hastiferella (Walker, 1866); syn. (Alata) 5744 4113

decipiens Staudinger, 1870;

syn. infrasubspecific, aberration (Etiella) 5744 4113

spartiella Rondani, 1876; syn. (Etiella) 5744 4113

sabulinus (Butler, 1879); syn. (Crambus) 5744 4113

madagascariensis Saalmüller, 1880; syn. (Etiella) 5744 4113

schisticolor Zeller, 1881; syn. (Etiella) 5744 4113

villosella Hulst, 1887; syn. (Etiella) 5744 4113

rubribasella Hulst, 1890; syn. (Etiella) 5744 4113

**Glyptocera** Ragonot, 1889

**consobrinella** (Zeller, 1872); valid (Nephopteryx) 5745

busckella (Dyar, 1904); syn. (Ambesa) 5745

**Pima** Hulst, 1888

**boisduvaliella** (Guenée, 1845); valid (Epischnia) 5746

farrella (Curtis, 1850); syn. (Anerastia) 5746

lafauryiella (Constant, 1865); syn. (Myelois) 5746

**albiplagiatella** (Packard, 1874); valid (Myelois) 5747 4114

**occidentalis** Heinrich, 1956^242^; valid (Pima) 5747 4114

**fosterella** Hulst, 1888; valid (Pima) 5748

**albocostalialis** (Hulst, 1886); valid (Ephestia) 5750 4115

subcostella (Ragonot, 1887)^243^; syn. (Epischnia) 5750 4115

albocostalis (Hulst, 1890); syn. emendation (Epischnia) 5750 4115

**fulvirugella** (Ragonot, 1887); valid (Epischnia) 5751

vividella (McDunnough, 1935)^244^; syn. (Epischnia) 5749

**granitella** (Ragonot, 1887); valid (Epischnia) 5752 4116

piperella (Dyar, 1904); syn. (Megasis) 5752 4116

**parkerella** (Schaus, 1924); valid (Epischnia) 5753

**fergusoni** Neunzig, 2003^245^; valid (Pima)

**Pimodes** A. Blanchard, 1976

**insularis** A. Blanchard, 1976; valid (Pimodes) 5754 4117

**caliginosus** Neunzig, 2003^246^; valid (Pimodes)

**Interjectio** Heinrich, 1956

**denticulella** (Ragonot, 1887); valid (Pristophora) 5755

ruderella (Ragonot, 1887)^247^; syn. (Epischnia) 5757

**columbiella** (McDunnough, 1935); valid (Ambesa) 5756

**niviella** (Hulst, 1888); valid (Lipographis) 5758

**Ambesa** Grote, 1880

**laetella** Grote, 1880; valid (Ambesa) 5759

**walsinghami** (Ragonot, 1887); valid (Pristophora) 5760 4118

monodon Dyar, 1913; syn. (Ambesa) 5760 4118

**mirabella** Dyar, 1908^248^; valid (Ambesa) 5760 4118

**lallatalis** (Hulst, 1886); valid (Nephopteryx) 5761

**dentifera** Neunzig, 2003^249^; valid (Ambesa)

**Catastia** Hübner, 1825

Diosia Duponchel, 1832

**bistriatella** (Hulst, 1895); valid (Pyla) 5762

**incorruscella** (Hulst, 1895); valid (Pyla) 5763

**actualis** (Hulst, 1886); valid (Nephopteryx) 5764

**subactualis** Neunzig, 2003^250^; valid (Catastia)

**Glyphocystis** A. Blanchard, 1973

**viridivallis** A. Blanchard, 1973; valid (Glyphocystis) 5765 4119

**Immyrla** Dyar, 1906

**nigrovittella** Dyar, 1906; valid (Immyrla) 5766

**Oreana** Hulst, 1888

**unicolorella** (Hulst, 1887); valid (Dioryctria) 5767

leucophaella (Hulst, 1892); syn. (Myelois) 5767

**Olybria** Heinrich, 1956

**aliculella** (Hulst, 1887); valid (Myelois) 5768 4120

oberthuriella (Ragonot, 1887); syn. (Salebria) 5768 4120

**furciferella** (Dyar, 1904); valid (Salebria) 5769

**Salebriacus** Heinrich, 1956

**odiosella** (Hulst, 1887); valid (Nephopteryx) 5770 4121

bakerella (Dyar, 1904); syn. (Salebria) 5770 4121

yumaella (Dyar, 1905); syn. (Salebria) 5770 4121

**Salebriaria** Heinrich, 1956

**ademptandella** (Dyar, 1908)^251^; valid (Salebria) 5771

**turpidella** (Ragonot, 1888); valid (Salebria) 5771

**nubiferella** (Ragonot, 1887); valid (Salebria) 5772

**borealis** Neunzig, 1988^252^; valid (Salebriaria)

**chisosensis** Neunzig, 1988^252^; valid (Salebriaria) 4122

**engeli** (Dyar, 1906); valid (Salebria) 5773

**roseopunctella** Neunzig, 2003^253^; valid (Salebriaria)

**fasciata** Neunzig, 1988^252^; valid (Salebriaria)

**rufimaculatella** Neunzig, 1988^252^; valid (Salebriaria)

**annulosella** (Ragonot, 1887); valid (Nephopteryx) 5774

**robustella** (Dyar, 1908)^251^; valid (Salebria) 5774

**bella** Neunzig, 1988^252^; valid (Salebriaria)

**fergusonella** (A. Blanchard & Knudson, 1983)^254^, ^255^;

valid (Psorosina)

**grandidentalis** Neunzig, 1988^252^; valid (Salebriaria) 4124

**tenebrosella** (Hulst, 1887); valid (Nephopteryx) 5775

quercicolella (Ragonot, 1887); syn. (Nephopteryx) 5775

heinrichalis (Dyar, 1917); syn. (Salebria) 5775

**equivoca** Neunzig, 1988^252^; valid (Salebriaria) 4123

**integra** Neunzig, 1988^252^; valid (Salebriaria)

**maximella** Neunzig, 1988^252^; valid (Salebriaria)

**simpliciella** Neunzig, 1988^252^; valid (Salebriaria)

**carolynae** Neunzig, 1988^252^; valid (Salebriaria)

**kanawha** Neunzig, 2003^253^; valid (Salebriaria)

**squamopalpiella** Neunzig, 1988^252^; valid (Salebriaria)

**floridana** Neunzig, 2003^253^; valid (Salebriaria)

**pallidella** Neunzig, 2003^253^; valid (Salebriaria)

**pumilella** (Ragonot, 1887); valid (Salebria) 5776

georgiella (Hulst, 1895); syn. (Salebria) 5776

**Quasisalebria** Heinrich, 1956

**atratella** (A. Blanchard & Knudson, 1985)^256^, ^257^;

valid (Salebriaria)

**fructetella** (Hulst, 1892)^257^; valid (Myelois) 5777

rectistrigella (Dyar, 1908); syn. (Salebria) 5777

**occidentalis** (Neunzig, 1988)^257^, ^258^; valid (Salebriaria)

**admixta** Heinrich, 1956; valid (Quasisalebria) 5779 4125

**Ortholepis** Ragonot, 1887

Metriostola Ragonot, 1893

**jugosella** Ragonot, 1887; valid (Ortholepis) 5780

**myricella** McDunnough, 1958; valid (Ortholepis) 5781

**baloghi** Neunzig, 2003^259^; valid (Ortholepis)

**rhodorella** McDunnough, 1958; valid (Ortholepis) 5782

**pasadamia** (Dyar, 1917); valid (Immyrla) 5783

**Polopeustis** Ragonot, 1893

**arctiella** (Gibson, 1920); valid (Pyla) 5784

**Meroptera** Grote, 1882

Emmerita Hampson, 1930

**mirandella** Ragonot, 1893; valid (Meroptera) 5785 4127

**anaimella** A. Blanchard & Knudson, 1985^260^;

valid (Meroptera) 4126

**nevadensis** Neunzig, 2003^261^; valid (Meroptera)

**cviatella** Dyar, 1905; valid (Meroptera) 5786

**pravella** (Grote, 1878); valid (Pempelia) 5787

**abditiva** Heinrich, 1956; valid (Meroptera) 5788

**Sciota** Hulst, 1888^262^

Denticera Amsel, 1961

Apodentinodia Roesler, 1969

Clasperopsis Roesler, 1969

Paranephopterix Roesler, 1969

**subfuscella** (Ragonot, 1887); valid (Salebria) 5789

semiobscurella (Hulst, 1890); syn. (Salebria) 5789

**delassalis** (Hulst, 1886); valid (Nephopteryx) 5790

purpurella (Hulst, 1892); syn. (Salebria) 5790

pudibundella (Ragonot, 1893); syn. (Salebria) 5790

**fraudifera** (Heinrich, 1956)^263^, ^264^; valid (Nephopteryx)

**rubescentella** (Hulst, 1900); valid (Mineola) 5791

**fernaldi** (Ragonot, 1887); valid (Salebria) 5792

**dammersi** (Heinrich, 1956); valid (Nephopteryx) 5793 4128

**floridensis** (Heinrich, 1956)^264^; valid (Nephopteryx) 5793

**vetustella** (Dyar, 1904); valid (Salebria) 5794

**inconditella** (Ragonot, 1893); valid (Salebria) 5795 4129

**subcaesiella** (Clemens, 1860); valid (Pempelia) 5796

contatella (Grote, 1880); syn. (Pempelia) 5796

**virgatella** (Clemens, 1860); valid (Pempelia) 5797

quinquepunctella (Grote, 1880); syn. (Pempelia) 5797

**carneella** (Hulst, 1887); valid (Nephopteryx) 5798

inquilinella (Ragonot, 1887); syn. (Nephopteryx) 5798

**basilaris** (Zeller, 1872); valid (Nephopteryx) 5799

**tarmitalis** (Hulst, 1886)^265^; valid (Pempelia) 5800

**levigatella** (Hulst, 1892)^264^; valid (Salebria) 5800

**californiana** Neunzig, 2003^266^; valid (Sciota)

**yuconella** (Dyar, 1925)^264^; valid (Salebria) 5800

**bifasciella** (Hulst, 1887); valid (Nephopteryx) 5801 4130

nogalesella (Dyar, 1905); syn. (Salebria) 5801 4130

**uvinella** (Ragonot, 1887); valid (Meroptera) 5802

afflictella (Hulst, 1900); syn. (Salebria) 5802

liquidambarella (Dyar, 1904); syn. (Meroptera) 5802

**celtidella** (Hulst, 1890); valid (Salebria) 5803 4131

**rubrisparsella** (Ragonot, 1887); valid (Pristophora) 5804

rufibasella (Ragonot, 1887); syn. (Nephopteryx) 5804

croceella Hulst, 1888; syn. (Sciota) 5804

texanella (Hulst, 1900); syn. (Psorosa) 5804

**gilvibasella** (Hulst, 1890); valid (Nephopteryx) 5805 4132

lacteella (Hulst, 1900); syn. (Salebria) 5805 4132

**quasisubfuscella** Neunzig, 2003^266^; valid (Sciota)

**crassifasciella** (Ragonot, 1887); valid (Nephopteryx) 5806

decipientella (Dyar, 1905); syn. (Nephopteryx) 5806

crataegella (Barnes & McDunnough, 1917);

syn. (Nephopteryx) 5806

**Tlascala** Hulst, 1890

**reductella** (Walker, 1863); valid (Nephopterix) 5808 4134

gleditschiella (Fernald, 1881); syn. (Pempelia) 5808 4134

**Tulsa** Heinrich, 1956

**finitella** (Walker, 1863); valid (Nephopteryx) 5809 4135

melanellus (Hulst, 1890); syn. (Elasmopalpus) 5809 4135

**umbripennis** (Hulst, 1895); valid (Pinipestis) 5810

gillettella (Dyar, 1904); syn. (Ortholepis) 5810

**oregonella** (Barnes & McDunnough, 1918); valid (Tlascala) 5811

**Telethusia** Heinrich, 1956

**ovalis** (Packard, 1873); valid (Pempelia) 5812

latisfasciatella (Packard, [1874]); syn. (Nephopteryx) 5812

rhypodella (Hulst, 1887)^267^; syn. (Glyptoteles) 5813

geminipunctella (Ragonot, 1893); syn. (Nephopteryx) 5812

modestella (Hulst, 1900); syn. (Nephopteryx) 5812

**Phobus** Heinrich, 1956

**brucei** (Hulst, 1895); valid (Dioryctria) 5814

**funerella** (Dyar, 1925); valid (Salebria) 5815

**curvatella** (Ragonot, 1887); valid (Nephopteryx) 5816 4139

**incertus** Heinrich, 1956; valid (Phobus) 5817

**Actrix** Heinrich, 1956

**nyssaecolella** (Dyar, 1904); valid (Tacoma) 5818

**dissimulatrix** Heinrich, 1956; valid (Actrix) 5819

**Stylopalpia** Hampson, 1901

**scobiella** (Grote, 1880); valid (Nephopteryx) 5820 4141

decimerella (Hulst, 1888); syn. (Lipographis) 5820 4141

**luniferella** Hampson, 1901^268^, ^269^; valid (Stylopalpia) 4140

**Hypochalcia** Hübner, 1825

Araxes Stephens, 1834

**ahenella** (Denis & Schiffermüller, 1775); valid (Tinea)

aeneella (Hübner, 1796); syn. (Tinea)

obscuratus (Haworth, 1796); syn. (Crambus)

tetrix (Haworth, 1811); syn. (Palparia)

rubiginella (Treitschke, 1832); syn. (Phycis)

bistrigella (Duponchel, 1836); syn. (Phycis)

fulginella (Duponchel, 1836); syn. (Phycis)

arduella (Bruand, 1848); syn. (Oncocera)

luridella (Schläger, 1848); syn. (Phycis)

bruneo-violaceella (Bruand, 1850); syn. (Phycis)

ghilianii Staudinger, 1870; syn. (Hypochalcia)

fasciatella Staudinger, 1881; syn. (Hypochalcia)

hulstiella Ragonot, 1887^270^; valid (Hypochalcia) 5821

caucasica Ragonot, 1893; syn. (Hypochalcia)

lugubrella E. M. Hering, 1924; syn. (Hypochalcia)

robustella Toll, 1938; syn. (Hypochalcia)

**Pyla** Grote, 1882

Pyla Ragonot, 1887^5^

Matilella Leraut 2001^5^

**fasciolalis** (Hulst, 1886); valid (Pinipestis) 5822

**impostor** Heinrich, 1956; valid (Pyla) 5823

**aequivoca** Heinrich, 1956; valid (Pyla) 5824

gaspeensis McDunnough, 1958^271^; syn. (Pyla) 5825

**araeneola** Balogh & Wilterding, 1998^272^; valid (Pyla)

**insinuatrix** Heinrich, 1956; valid (Pyla) 5826

**aenigmatica** Heinrich, 1956; valid (Pyla) 5827

**criddlella** Dyar, 1907; valid (Pyla) 5828

**fusca** (Haworth, 1811); valid (Phycis) 5829

spadicella (Zincken, 1818); syn. (Phycis) 5829

carbonariella (F. v. Röslerstamm, 1834–1843);

syn. (Phycis) 5829

janthinella (Duponchel, 1836); syn. (Phycis) 5829

posticella (Zetterstedt, 1839)^273^; syn. (Phycis)

annulatella (Zetterstedt, 1839)^273^; syn. (Phycis)

bilineata (Curtis, 1850)^273^; syn. (Phycita)

moestella (Walker, 1863); syn. (Nephopteryx) 5829

procellariana (Walker, 1863); syn. (Paedisca) 5829

frigidella (Packard, 1867); syn. (Eudorea) 5829

cacabella (Hulst, 1887); syn. (Pinipestis) 5829

triplagiatella (Dyar, 1904); syn. (Salebria) 5829

**hypochalciella** (Ragonot, 1887); valid (Nephopteryx) 5830

blackmorella Dyar, 1921; syn. (Pyla) 5830

**hanhamella** Dyar, 1904; valid (Pyla) 5831

**westerlandi** Wilterding & Balogh, 2002^274^; valid (Pyla)

**scintillans** (Grote, 1881); valid (Nephopteryx) 5832

feella Dyar, 1921; syn. (Pyla) 5832

sylphiella Dyar, 1921; syn. (Pyla) 5832

**longispina** Neunzig, 2003^275^; valid (Pyla)

**serrata** Neunzig, 2003^275^; valid (Pyla)

**rainierella** Dyar, 1904; valid (Pyla) 5833

**aeneella** Hulst, 1895; valid (Pyla) 5834

**aeneoviridella** Ragonot, 1887; valid (Pyla) 5835

**metalicella** Hulst, 1895; valid (Pyla) 5836

**fasciella** Barnes & McDunnough, 1917; valid (Pyla) 5837

**nigricula** Heinrich, 1956; valid (Pyla) 5838

**viridisuffusella** Barnes & McDunnough, 1917; valid (Pyla) 5839

**Utah** Ferris, 2012^276^

**sanrafaelensis** Ferris, 2012; valid (Utah)

**Phycitopsis** Ragonot, 1887

**flavicornella** Ragonot, 1887;

valid identity uncertain (Phycitopsis) 5840

**Dioryctria** Zeller, 1846

Pinipestis Grote, 1878

Dioryctroides Mutuura & Munroe, 1974^277^

Ocrisia Ragonot, 1893^277^

**abietivorella** (Grote, 1878); valid (Pinipestis) 5841 4145

reniculella (Grote, 1880); syn. (Pinipestis) 5841 4145

elegantella (Hulst, 1892); syn. (Myelois) 5841 4145

**taedae** Schaber & Wood, 1971; valid (Dioryctria) 5842

**reniculelloides** Mutuura & Munroe, 1973; valid (Dioryctria) 5843

**pseudotsugella** Munroe, 1959; valid (Dioryctria) 5844

**rossi** Munroe, 1959; valid (Dioryctria) 5845 4154

**auranticella** (Grote, 1883); valid (Nephopteryx) 5846 4151

miniatella Ragonot, 1887; syn. (Dioryctria) 5846 4151

xanthaenobares Dyar, 1911; syn. (Dioryctria) 5846 4151

**disclusa** Heinrich, 1953; valid (Dioryctria) 5847

**erythropasa** (Dyar, 1914); valid (Pinipestis) 5848 4152

**pygmaeella** Ragonot, 1887; valid (Dioryctria) 5849

**ponderosae** Dyar, 1914; valid (Dioryctria) 5850

**okanaganella** Mutuura, Munroe & Ross, 1969;

valid (Dioryctria) 5851

**hodgesi** Neunzig, 2003^278^; valid (Dioryctria)

**zimmermani** Grote, 1877;

valid (Nephopteryx (Dioryctria)) 5852

austriana (Cosens, 1906); syn. (Retinia) 5852

**resinosella** Mutuura, 1982^279^; valid (Dioryctria)

**delectella** (Hulst, 1895)^280^; valid (Salebria) 5852

**amatella** (Hulst, 1887); valid (Nephopteryx) 5853

**yatesi** Mutuura & Munroe, 1979^281^; valid (Dioryctria)

**cambiicola** (Dyar, 1914); valid (Pinipestis) 5854

**tumicolella** Mutuura, Munroe & Ross, 1969;

valid (Dioryctria) 5855

**contortella** Mutuura, Munroe & Ross, 1969;

valid (Dioryctria) 5856

**monticolella** Mutuura, Munroe & Ross, 1969;

valid (Dioryctria) 5857

**banksiella** Mutuura, Munroe & Ross, 1969;

valid (Dioryctria) 5858

**albovittella** (Hulst, 1890); valid (Pinipestis) 5859

**westerlandi** Donahue & Neunzig, 2002^282^; valid (Dioryctria)

**fordi** Donahue & Neunzig, 2002^282^; valid (Dioryctria)

**mutuurai** Neunzig, 2003^278^; valid (Dioryctria)

**baumhoferi** Heinrich, 1956; valid (Dioryctria) 5860

**pentictonella** Mutuura, Munroe & Ross, 1969;

valid (Dioryctria) 5861

**muricativorella** Neunzig, 2003^278^; valid (Dioryctria)

**vancouverella** Mutuura, Munroe & Ross, 1969^280^;

valid (Dioryctria) 5861

**durangoensis** Mutuura & Neunzig, 1986^283^;

valid (Dioryctria) 4160

**gulosella** (Hulst, 1890); valid (Acrobasis) 5862

**subtracta** Heinrich, 1956; valid (Dioryctria) 5863

**clarioralis** (Walker, 1863); valid (Nephopteryx) 5863.1

brunneella (Dyar, 1904); syn. (Ulophora) 5863.1

**inyoensis** Neunzig, 2003^278^; valid (Dioryctria)

**sierra** Neunzig, 2003^278^; valid (Dioryctria)

**caesirufella** A. Blanchard & Knudson, 1983^284^;

valid (Dioryctria)

**ebeli** Mutuura & Munroe, 1979^281^; valid (Dioryctria)

**merkeli** Mutuura & Munroe, 1979^281^; valid (Dioryctria)

**taedivorella** Neunzig & Leidy, 1989^285^; valid (Dioryctria)

**Oryctometopia** Ragonot, 1888

**fossulatella** Ragonot, 1888; valid (Oryctometopia) 5864 4162

moeschleri (Ragonot, 1888); syn. (Phycita) 5864 4162

**Sarata** Ragonot, 1887

**edwardsialis** (Hulst, 1886); valid (Megaphycis) 5865

polyphemella (Ragonot, 1887); syn. (Megaphycis) 5865

**pullatella** (Ragonot, 1887); valid (Megasis) 5866

**punctella** (Dyar, 1914); valid (Megasis) 5867 4165

septentrionaria Heinrich, 1956^286^; syn. (Sarata) 5867 4165

**incanella** Hulst, 1895; valid (Sarata) 5868

aridella (Dyar, 1905); syn. (Megasis) 5868

**atrella** (Hulst, 1890); valid (Megasis) 5869

**caudellella** (Dyar, 1904)^287^; valid (Megasis) 5870

**dophnerella** Ragonot, 1887; valid (Sarata) 5871

**nigrifasciella** Ragonot, 1887; valid (Sarata) 5872

**cinereella** Hulst, 1900; valid (Sarata) 5873

**rubrithoracella** (Barnes & McDunnough, 1913);

valid (Megasis) 5874

**tephrella** Ragonot, 1893; valid (Sarata) 5875

**alpha** Heinrich, 1956^286^; valid unassociated female (Sarata) 5876

**beta** Heinrich, 1956; valid unassociated female (Sarata) 5877

**gamma** Heinrich, 1956; valid unassociated female (Sarata) 5878

**iota** Heinrich, 1956; valid unassociated female (Sarata) 5879

**perfuscalis** (Hulst, 1886); valid (Nephopteryx) 5880

excantalis (Hulst, 1886); syn. (Anerastia) 5880

**epsilon** Heinrich, 1956; valid unassociated female (Sarata) 5881

**phi** Heinrich, 1956; valid unassociated female (Sarata) 5882

**kappa** Heinrich, 1956; valid unassociated female (Sarata) 5883

**delta** Heinrich, 1956; valid unassociated female (Sarata) 5884

**Philodema** Heinrich, 1956

**rhoiella** (Dyar, 1904); valid (Sarata) 5885

**Lipographis** Ragonot, 1887

**fenestrella** (Packard, 1873); valid (Pempelia) 5886 4166

leoninella (Packard, 1873)^288^; syn. (Pempelia) 5887

humilis Ragonot, 1887; syn. (Lipographis) 5886 4166

pallidella (Dyar, 1904); syn. (Pyla) 5887

**truncatella** (W. S. Wright, 1916); valid (Hypochalcia) 5888 4167

**umbrella** (Dyar, 1908); valid (Sarata) 5889 4168

**unicolor** Ferris, 2012^289^; valid (Lipographis)

**Quasisarata** Neunzig, 2003^290^

**subosseella** (Hulst, 1892)^291^; valid (Lipographis) 4169

**Adelphia** Heinrich, 1956

**petrella** (Zeller, 1846); valid (Pempelia) 5890 4170

rubiginella (Walker, 1863); syn. (Nephopteryx) 5890 4170

rufinalis (Walker, 1863); syn. (Nephopteryx) 5890 4170

hapsella (Hulst, 1887); syn. (Nephopteryx) 5890 4170

**Pseudadelphia** Neunzig, 2003^292^

**ochripunctella** (Dyar, 1908); valid (Salebria) 5891 4171

**Ufa** Walker, 1863

**lithosella** (Ragonot, 1887); valid (Selagia) 5892 4173

luteella (Hulst, 1900); syn. (Honora) 5892 4173

roseitinctella (Dyar, 1912)^293^; syn. (Ancylostomia) 5893 4174

**senta** Heinrich, 1956; valid (Ufa) 5894 4175

**rubedinella** (Zeller, 1848); valid (Pempelia) 5895 4176

translucida (Walker, 1863); syn. (Acrobasis) 5895 4176

rufescentalis (Walker, 1863); syn. (Nephopteryx) 5895 4176

minualis (Walker, 1863); syn. (Nephopteryx) 5895 4176

deprivalis (Walker, 1863); syn. (Nephopteryx) 5895 4176

venezuelalis Walker, 1863; syn. (Ufa) 5895 4176

pyrrhochrellus (Ragonot, 1888); syn. (Elasmopalpus) 5895 4176

**Elasmopalpus** Blanchard, 1852

**lignosellus** (Zeller, 1848); valid (Pempelia) 5896 4178

angustellus Blanchard, 1852; syn. (Elasmopalpus) 5896 4177

tartarella (Zeller, 1872); syn. (Pempelia) 5896 4178

incautella (Zeller, 1872); syn. (Pempelia) 5896 4178

major (Zeller, 1874); syn. (Pempelia) 5896 4178

anthracellus Ragonot, 1888; syn. (Elasmopalpus) 5896 4178

carbonella (Hulst, 1888); syn. (Dasypyga) 5896 4178

puer Dyar, 1919; syn. (Elasmopalpus) 5896 4178

**Acroncosa** Barnes & McDunnough, 1917

**albiflavella** Barnes & McDunnough, 1917; valid (Acroncosa) 5897

**minima** Neunzig, 2003^294^; valid (Acroncosa)

**castrella** Barnes & McDunnough, 1917^295^; valid (Acroncosa) 5897

**similella** Barnes & McDunnough, 1917; valid (Acroncosa) 5898

**Passadena** Hulst, 1900

**flavidorsella** (Ragonot, 1887); valid (Anoristia) 5899 4179

canescentella (Hulst, 1890); syn. (Meroptera) 5899 4179

constantella Hulst, 1900; syn. (Passadena) 5899 4179

cinctella (Hulst, 1900); syn. (Megasis) 5899 4179

**Passadenoides** Neunzig, 2003^296^

**donahuei** Neunzig, 2003; valid (Passadenoides)

**pullus** Neunzig, 2003; valid (Passadenoides)

**montanus** Ferris, 2004^297^; valid (Passadenoides)

**Chorrera** Dyar, 1914

**extrincica** (Dyar, 1919)^298^; valid (Rhodophaea) 4185

**Ulophora** Ragonot, 1890

Acromeseres Dyar, 1919

**groteii** Ragonot, 1890; valid (Ulophora) 5900

tephrosiella Dyar, 1904; syn. (Ulophora) 5900

**Tacoma** Hulst, 1888

**feriella** Hulst, 1888; valid (Tacoma) 5901 4187

submedianella Dyar, 1913; syn. (Tacoma) 5901 4187

**Ragonotia** Grote, 1888; repl. name

Ciris Ragonot, 1887; preocc. by Koch, 1846

Psammia Hampson, 1930

**dotalis** (Hulst, 1886); valid (Anerastia) 5902 4188

discigerella (Ragonot, 1887); syn. (Ciris) 5902 4188

olivella (Hulst, 1888); syn. (Anoristia) 5902 4188

saganella Hulst, 1890; syn. (Ragonotia) 5902 4188

indianella (Dyar, 1923); syn. (Megasis) 5902 4188

megasis (Dyar, 1923)^5^; syn. (Megasis)

flavipicta (Hampson, 1930); syn. (Psammia) 5902 4188

**Martia** Ragonot, 1887

Urula Hulst, 1900

**arizonella** Ragonot, 1887; valid (Martia) 5903 4190

incongruella (Hulst, 1900); syn. (Urula) 5903 4190

**Eumysia** Dyar, 1925

**mysiella** (Dyar, 1905); valid (Yosemitia) 5904 4193

**maidella** (Dyar, 1905); valid (Yosemitia) 5905 4194

**pallidipennella** (Hulst, 1895)^299^; valid (Volusia) 4195

**fuscatella** (Hulst, 1900); valid (Zophodia) 5906 4196

**semicana** Heinrich, 1956; valid (Eumysia) 5907

**idahoensis** Mackie, 1958; valid (Eumysia) 5908

**Arcola** Shaffer, 1995

Vogtia Pastrana, 1961; preocc. by (Kölliker, 1853)

**malloi** (Pastrana, 1961)^300^; valid introduced (Vogtia) 4202

**Macrorrhinia** Ragonot, 1887

Dolichorrhinia Ragonot, 1888; repl. name

Ocala Hulst, 1892^301^

Divitiaca Barnes & McDunnough, 1913^301^

**ochrella** (Barnes & McDunnough, 1913); valid (Divitiaca) 5909 4198

simulella (Barnes & McDunnough, 1913)^302^;

syn. (Divitiaca) 5910 4199

**parvulella** (Barnes & McDunnough, 1913); valid (Divitiaca) 5911 4197

**a. consociata** (Heinrich, 1956)^5^; ssp. extralimital (Divitiaca)

**aureofasciella** Ragonot, 1887; valid (Macrorrhinia) 5912 4203

**endonephele** (Hampson, 1918)^303^; valid (Rinaphe) 4200

ignetincta (Hampson, 1918)^303^, ^304^; syn. (Rinaphe) 4201

signifera A. Blanchard, 1976^304^; syn. (Macrorrhinia) 5913 4203

**dryadella** (Hulst, 1892); valid (Ocala) 5914 4207

platanella (Grossbeck, 1917); syn. (Dolichorrhinia) 5914 4207

**Maricopa** Hulst, 1890^305^

Valdivia Ragonot, 1888; preocc. by White, 1847

**lativittella** (Ragonot, 1887); valid (Ciris) 5915 4209

aureomaculella (Dyar, 1903); syn. (Zophodia) 5915 4209

**Protasia** Heinrich, 1956

**mirabilicornella** (Dyar, 1908)^306^; valid (Valdivia) 4211

**Ancylosis** Zeller, 1839

Heterographis Ragonot, 1885^5^

Staudingeria Ragonot, 1887^307^

Hedemannia Ragonot, 1887^5^

Syria Ragonot, 1887^5^

Mona Hulst, 1888; preocc. by Reichenbach, 1863

Cabotia Ragonot, 1888^5^

Hypographia Ragonot, 1890^5^

Hypogryphia Ragonot, 1890^5^; unnecessary replac. name

Encystia Hampson, 1901

Hulstia Hampson, 1901^5^

Harnocha Dyar, 1914^5^

Acornigerula Amsel, 1935

Cornigerula Amsel, 1935

Iransharia Amsel, 1959^5^

Pseudocabotia A. Blanchard & Knudson, 1985^308^, ^309^

**morrisonella** (Ragonot, 1887); valid (Heterographis) 5916 4212

coloradensis (Ragonot, 1887); syn. (Heterographis) 5916 4212

olbiella (Hulst, 1888); syn. (Mona) 5916 4212

ignistrigella (Ragonot, 1901); syn. (Heterographis) 5916 4212

palloricostella (Walter, 1928); syn. (Honora) 5916 4212

**albipenella** (Hulst, 1887); valid (Pempelia) 5917 4213

olivacella (Dyar, 1904); syn. (Staudingeria) 5917 4213

perluteella (Dyar, 1904); syn. (Staudingeria) 5917 4213

**undulatella** (Clemens, 1860); valid (Nephopteryx) 5918 4214

rubiginalis (Walker, [1866]); syn. (Scoparia) 5918 4214

obsipella (Hulst, 1888); syn. (Honora) 5918 4214

oblitella (Ragonot, 1889);

syn. not Zeller, 1848 (Heterographis) 5918 4214

fumosella (Hulst, 1900); syn. (Honora) 5918 4214

**bonhoti** (Hampson, 1901)^310^; valid (Encystia) 4225

**balconiensis** (A. Blanchard & Knudson, 1985)^308^;

valid (Pseudocabotia) 4226

**Honora** Grote, 1878

**mellinella** Grote, 1878; valid (Honora) 5919 4215

ochrimaculella Ragonot, 1887; syn. (Honora) 5919 4215

**subsciurella** Ragonot, 1887; valid (Honora) 5920

**sciurella** Ragonot, 1887; valid (Honora) 5921

**dotella** Dyar, 1910; valid (Honora) 5922 4216

**montinatatella** (Hulst, 1887); valid (Spermatophthora) 5923

canicostella Ragonot, 1887; syn. (Honora?) 5923

**perdubiella** (Dyar, 1905); valid (Zophodia) 5924

**dulciella** Hulst, 1900^29^; valid (Honora) 5925

**Canarsia** Hulst, 1890

Canarsiana Strand, 1920

**ulmiarrosorella** (Clemens, 1860); valid (Nephopteryx) 5926 4227

pneumatella (Hulst, 1887); syn. (Stenoptycha) 5926 4227

ulmella (Ragonot, 1887); syn. (Psorosa) 5926 4227

fuscatella (Hulst, 1888); syn. (Honora) 5926 4227

gracilella Hulst, 1900; syn. (Canarsia) 5926 4227

feliculella Dyar, 1907; syn. (Canarsia) 5926 4227

discocellularis (Strand, 1920); syn. (Canarsiana)

**Eurythmidia** Hampson, 1901

**ignidorsella** (Ragonot, 1887); valid (Eurythmia) 5927 4230

**Wunderia** Grossbeck, 1917

**neaeriatella** Grossbeck, 1917; valid (Wunderia) 5928 4231

**Diviana** Ragonot, 1888

Dannemora Hulst, 1890

**eudoreella** Ragonot, 1888; valid (Diviana) 5929

edentella (Hulst, 1890); syn. (Dannemora) 5929

**Palatka** Hulst, 1892

Palatka Hulst, 1891; nomen nudum

**nymphaeella** (Hulst, 1892); valid (Diviana) 5930 4234

verecuntella (Grossbeck, 1917); syn. (Diviana) 5930 4234

**powelli** Neunzig & Solis, 1996^311^; valid (Palatka)

**Psorosina** Dyar, 1904

**hammondi** (Riley, 1872); valid (Pempelia) 5931

angulella Dyar, 1904; syn. (Psorosina) 5931

**Patriciola** Heinrich, 1956

**semicana** Heinrich, 1956; valid (Patriciola) 5932

**Anderida** Heinrich, 1956

**sonorella** (Ragonot, 1887);

valid emendation (Euzophera) 5933 4239

senorella (Ragonot, 1887);

syn. incorrect original spelling (Euzophera) 5933 4239

placidella (Dyar, 1908); syn. (Euzophera) 5933 4239

**peorinella** A. Blanchard & Knudson, 1985^312^; valid (Anderida) 4240

**Cassiana** Heinrich, 1956

**malacella** (Dyar, 1914)^313^; valid (Vitula) 4241

**Mescinia** Hampson, 1901

**estrella** Barnes & McDunnough, 1913; valid (Mescinia) 5934 4245

**berosa** Dyar, 1914^314^; valid (Mescinia) 4250

**parvula** (Zeller, 1881)^315^; valid (Ephestia) 4247

neoparvula Neunzig & Dow, 1993^316^; syn. (Mescinia) 4248

**texanica** Neunzig, 1997^317^; valid (Mescinia)

**Phestinia** Hampson, 1930

**costella** Hampson, 1930^318^; valid (Phestinia) 4256

**Comotia** Dyar, 1914

**torsicornis** Dyar, 1914^319^; valid (Comotia) 4257

**Homoeosoma** Curtis, 1833

Phycidea Zeller, 1839

Lotria Guenée, 1845

Anhomoeosoma Roesler, 1965^5^

**electella** (Hulst, 1887); valid (Anerastia) 5935 4267

opalescella (Hulst, 1887); syn. (Ephestia) 5935 4267

texanella Ragonot, 1887; syn. (Homoeosoma) 5935 4267

tenuipunctella Ragonot, 1887; syn. (Homoeosoma) 5935 4267

differtella Barnes & McDunnough, 1913;

syn. (Homoeosoma) 5935 4267

**phaeoboreas** Goodson & Neunzig, 1993^320^;

valid (Homoeosoma)

**stypticella** Grote, 1878; valid (Homeosoma) 5936

**striatellum** Dyar, 1905; valid (Homoeosoma) 5937 4274

breviplicitum Heinrich, 1956^321^; syn. (Homoeosoma) 5938 4274

imitator Heinrich, 1956^321^; syn. (Homoeosoma) 5940 4274

**asylonnastes** Goodson & Neunzig, 1993^320^;

valid (Homoeosoma)

**oslarellum** Dyar, 1905; valid (Homoeosoma) 5938 4270

**parvalbum** A. Blanchard & Knudson, 1985^322^;

valid (Homoeosoma) 4272

**oxycercus** Goodson & Neunzig, 1993^320^; valid (Homoeosoma) 4271

**illuviella** Ragonot, 1888; valid (Homoeosoma) 5939 4269

candidella Hulst, 1888; syn. (Homoeosoma) 5939 4269

**emendator** Heinrich, 1956^323^; valid (Homoeosoma) 5939

**albescentella** Ragonot, 1887; valid (Homoeosoma) 5941 4266

elongellum Dyar, 1903; syn. (Homoeosoma) 5941 4266

**impressalis** Hulst, 1886; valid (Homeosoma) 5942

**inornatella** (Hulst, 1900); valid (Euzophera) 5943

**nanophasma** Neunzig, 1997^324^; valid (Homoeosoma)

**uncanalis** (Hulst, 1886); valid (Nephopteryx) 5936

**deceptorium** Heinrich, 1956; valid (Homoeosoma) 5944

**ammonastes** Goodson & Neunzig, 1993^320^;

valid (Homoeosoma)

**pedionnastes** Goodson & Neunzig, 1993^320^;

valid (Homoeosoma) 4273

**eremophasma** Goodson & Neunzig, 1993^320^;

valid (Homoeosoma) 4268

**ardaloniphas** Goodson & Neunzig, 1993^320^;

valid (Homoeosoma)

**Patagonia** Ragonot, 1901

**peregrinum** (Heinrich, 1956); valid (Homoeosoma) 5945 4294

**Phycitodes** Hampson, 1917^325^

Rotruda Heinrich, 1956

**mucidella** (Ragonot, 1887)^326^; valid (Homoeosoma) 5946 4295

**reliquellum** (Dyar, 1904)^326^; valid (Homoeosoma) 5946 4295

**Unadilla** Hulst, 1890

Strymax Dyar, 1914

**maturella** (Zeller, 1881); valid (Homoeosoma) 4299

floridensis Heinrich, 1956^327^; syn. (Unadilla) 5947 4301

**erronella** (Zeller, 1881); valid (Homoeosoma) 4298

ubacensis (Zeller, 1881); syn. (Homoeosoma) 4298

nasutella Hulst, 1890^328^; syn. (Unadilla) 5948

bipunctella (Hampson, 1901); syn. (Ephestia) 4298

dorae (Dyar, 1914); syn. (Strymax) 4298

pyllis (Dyar, 1914); syn. (Strymax) 4298

**Laetilia** Ragonot, 1889

**coccidivora** (J. H. Comstock, 1879); valid (Dakruma) 5949 4304

**a. cardini** Dyar, 1918; ssp. (Laetilia) 4304

**dilatifasciella** (Ragonot, 1887)^329^; valid (Zophodia) 5949 4304

**hulstii** Cockerell, 1897^329^; valid (Laetilia) 5949 4304

quadricolorella (Dyar, 1904); syn. (Atascosa) 5949 4304

**zamacrella** Dyar, 1925; valid (Laetilia) 5950

**myersella** Dyar, 1910; valid (Laetilia) 5951

**ephestiella** (Ragonot, 1887); valid (Dakruma) 5952

lustrella (Dyar, 1903); syn. (Maricopa) 5952

**fiskeella** Dyar, 1904; valid (Laetilia) 5953

**cinerosella** Neunzig, 1997^330^; valid (Laetilia)

**bellivorella** Neunzig, 1997^330^; valid (Laetilia)

**Rostrolaetilia** A. Blanchard & Ferguson, 1975

**placidella** (Barnes & McDunnough, 1918);

valid (Parramatta) 5954

**minimella** A. Blanchard & Ferguson, 1975;

valid (Rostrolaetilia) 5955

**placidissima** A. Blanchard & Ferguson, 1975;

valid (Rostrolaetilia) 5956

**utahensis** A. Blanchard & Ferguson, 1975;

valid (Rostrolaetiia) 5957

**coloradella** A. Blanchard & Ferguson, 1975;

valid (Rostrolaetilia) 5958

**eureka** A. Blanchard & Ferguson, 1975;

valid (Rostrolaetilia) 5959

**nigromaculella** (Hulst, [1901]); valid (Aurora) 5960 4310

**ardiferella** (Hulst, 1888); valid (Altoona) 5961 4311

**texanella** A. Blanchard & Ferguson, 1975;

valid (Rostrolaetilia) 5962 4312

**pinalensis** A. Blanchard & Ferguson, 1975;

valid (Rostrolaetilia) 5963 4313

**Welderella** A. Blanchard, 1978

**parvella** (Dyar, 1906); valid (Ollia) 5964 4314

**Baphala** Heinrich, 1956

**pallida** (J. H. Comstock, 1880)^331^; valid (Dakruma) 5949 4304

basimaculatella (Ragonot, 1887); syn. (Vitula) 5965

**eremiella** (Dyar, 1910)^332^; valid (Laetilia) 5965

**phaeolella** Neunzig, 1997^333^; valid (Baphala)

**Rhagea** Heinrich, 1956

**packardella** (Ragonot, 1887); valid (Zophodia) 5966 4322

orobanchella (Dyar, 1904); syn. (Zophodia) 5966 4322

**stigmella** (Dyar, 1910); valid (Zophodia) 5967 4321

maculicula (Dyar, 1913); syn. (Yosemitia) 5967 4321

**Zophodia** Hübner, [1825]

Dakruma Grote, 1878

**grossulariella** (Hübner, [1809]); valid (Tinea) 5968 4323

convolutella (Hübner, 1796)^5^; syn. (Tinea)

grossularialis Hübner, 1825; syn. emendation (Zophodia) 5968 4323

grossulariae (Riley, 1869); syn. (Pempelia) 5968 4323

turbatella (Grote, 1878); syn. (Dakruma) 5968 4323

franconiella (Hulst, 1890); syn. (Euzophera) 5968 4323

bella Hulst, 1892; syn. (Zophodia) 5968 4323

pallidella Lambillon, 1921^5^; syn. (Zophodia)

ihouna Dyar, 1925; syn. (Zophodia) 5968 4323

dilativitta Dyar, 1925; syn. (Zophodia) 5968 4323

magnificans Dyar, 1925; syn. (Zophodia) 5968 4323

**epischnioides** Hulst, 1900; valid misplaced (Zophodia) 5969

**multistriatella** (A. Blanchard & Knudson, 1981)^334^;

valid (Ozamia)

**Melitara** Walker, 1863

Megaphycis Grote, 1882

Olycella Dyar, 1928^335^

**prodenialis** Walker, 1863; valid (Melitara) 5970 4324

bollii (Zeller, 1872); syn. (Zophodia) 5970 4324

**dentata** (Grote, 1876); valid (Zophodia) 5971 4325

**texana** Neunzig, 1997^336^; valid (Melitara)

**doddalis** Dyar, 1925^337^; valid (Melitara) 5971 4325

**apicigrammella** A. Blanchard & Knudson, 1985^338^;

valid (Melitara) 4326

**junctolineella** Hulst, 1900; valid (Melitara) 5972 4327

**a. pectinatella** (Hampson, 1901); ssp. extralimital? (Olyca) 4327

**subumbrella** (Dyar, 1925); valid (Olyca) 5973 4329

**Alberada** Heinrich, 1939

**parabates** (Dyar, 1913); valid (Melitara) 5974 4331

**bidentella** (Dyar, 1908); valid (Zophodia) 5975 4332

holochlora (Dyar, 1925)^339^; syn. (Zophodia) 5976

**californiensis** Neunzig, 1997^340^; valid (Alberada)

**franclemonti** Neunzig, 1997^340^; valid (Alberada)

**candida** Neunzig, 1997^340^; valid (Alberada)

**Cactoblastis** Ragonot, 1901

Neopyralis Brèthes, 1920

**cactorum** (Berg, 1885)^341^; valid introduced (Zophodia) 4334

**Cahela** Heinrich, 1939

**ponderosella** (Barnes & McDunnough, 1918); valid (Olyca) 5977 4339

purgatoria (Dyar, 1925); syn. (Zophodia) 5977 4339

interstitialis (Dyar, 1925); syn. (Cactobrosis) 5977 4339

phoenicis (Dyar, 1925); syn. (Cactobrosis) 5977 4339

**Rumatha** Heinrich, 1939

**glaucatella** (Hulst, 1888); valid (Honora) 5978 4340

**bihinda** (Dyar, 1922); valid (Zophodia) 5979 4342

**jacumba** Neunzig, 1997^342^; valid (Rumatha)

**polingella** (Dyar, 1906); valid (Zophodia) 5980 4341

**Yosemitia** Ragonot, 1901

**graciella** (Hulst, 1887); valid (Spermatophthora) 5981 4343

longipennella (Hulst, 1888)^343^; syn. (Zophodia) 5982 4344

**fieldiella** (Dyar, 1913); valid (Zophodia) 5983

**Eremberga** Heinrich, 1939

**leuconips** (Dyar, 1925); valid (Cactobrosis) 5984 4349

**creabates** (Dyar, 1923); valid (Olyca) 5985 4350

**insignis** Heinrich, 1939; valid (Eremberga) 5986 4351

**Ozamia** Hampson, 1901

**fuscomaculella** (W. S. Wright, 1916); valid (Euzophera) 5987 4359

heliophila Dyar, 1925; syn. (Ozamia) 5987 4359

**clarefacta** Dyar, 1919^344^; valid (Ozamia) 5987 4359

**thalassophila** Dyar, 1925; valid (Ozamia) 5988 4360

**lucidalis** (Walker, 1863)^345^; valid (Trachonitis) 4358

**Cactobrosis** Dyar, 1914

**fernaldialis** (Hulst, 1886); valid (Megaphycis) 5989 4366

gigantella (Ragonot, 1888); syn. (Euzophera) 5989 4366

cinereella (Hulst, 1900); syn. (Honora) 5989 4366

**Echinocereta** Neunzig, 1997^346^

**strigalis** (Barnes & McDunnough, 1912); valid (Euzophera) 5991 4370

**Lascelina** Heinrich, 1956

**canens** Heinrich, 1956; valid (Lascelina) 5992 4373

**Metephestia** Hampson, 1901

**simplicula** (Zeller, 1881); valid (Ephestia) 5993 4374

**Selga** Heinrich, 1956

**arizonella** (Hulst, 1900); valid (Heterographis) 5994 3963

**californica** Neunzig, 1990^347^; valid (Selga)

**Pseudocabima** Heinrich, 1956

**arizonensis** Heinrich, 1956^348^; valid (Pseudocabima) 3994

**Euzophera** Zeller, 1867; repl. name

Stenoptycha Heinemann, 1865; preocc. by Agassiz, 1862

Melia Heinemann, 1865;

preocc. by Bosc [in Risso], 1813, repl. name

Pistogenes Meyrick, 1937

Ahwazia Amsel, 1949

Cymbalorissa Gozmány, 1958^5^

Longignathia Roesler, 1965

Quadrempista Roesler, 1973^5^

**semifuneralis** (Walker, 1863); valid (Nephopteryx) 5995 4390

pallulella (Hulst, 1887); syn. (Stenoptycha) 5995 4390

**aglaeella** Ragonot, 1887^349^; valid (Euzophera) 5995 4391

**habrella** Neunzig, 1990^350^; valid (Euzophera)

**vinnulella** Neunzig, 1990^350^; valid (Euzophera)

**magnolialis** Capps, 1964; valid (Euzophora) 5996

**ostricolorella** Hulst, 1890; valid (Euzophera) 5997

**nigricantella** Ragonot, 1887; valid (Euzophera) 5998 4392

griselda Dyar, 1913; syn. (Euzophera) 5998 4392

**Eulogia** Heinrich, 1956

**ochrifrontella** (Zeller, [1876]); valid (Ephestia) 5999

ferruginella (Ragonot, 1887); syn. (Euzophera) 5999

**Ephestiodes** Ragonot, 1887

**gilvescentella** Ragonot, 1887; valid (Ephestiodes) 6000 4398

nigrella Hulst, 1900; syn. (Ephestiodes) 6000 4398

**infimella** Ragonot, 1887; valid (Ephestiodes) 6001

**monticolus** Neunzig, 1990^351^; valid (Ephestiodes) 4399

**erythrella** Ragonot, 1887; valid (Ephestiodes) 6002

coloradella (Hulst, 1900); syn. (Eurythmia) 6002

benjaminella Dyar, 1904; syn. (Ephestiodes) 6002

**mignonella** Dyar, 1908; valid (Ephestiodes) 6003

**griseus** Neunzig, 1990^351^; valid (Ephestiodes)

**erasa** Heinrich, 1956; valid (Ephestiodes) 6004

**Australephestiodes** Neunzig, 1990^352^

**stictella** (Hampson, 1901)^353^; valid (Unadilla) 4404

uniformella (Hampson, 1901); syn. (Ephestiodes) 4404

granulella (Hampson, 1901); syn. (Ephestiodes) 4404

**Moodnodes** Neunzig, 1990^354^

**plorella** (Dyar, 1914)^355^; valid (Ephestiodes) 4406

vestilla (Dyar, 1914); syn. (Eurythmia) 4406

**Moodna** Hulst, 1890

**ostrinella** (Clemens, 1860); valid (Ephestia) 6005

obtusangulella (Ragonot, 1887); syn. (Hornigia) 6005

pelviculella Hulst, 1890; syn. (Moodna) 6005

**pallidostrinella** Neunzig, 1990^356^; valid (Moodna) 4410

**bisinuella** Hampson, 1901; valid (Moodna) 6006 4411

**Vitula** Ragonot, 1887

Hornigia Ragonot, 1887; preocc. by Ragonot, 1885

Manhatta Hulst, 1890^357^

**edmandsii** (Packard, 1865); valid (Nephopteryx) 6007

dentosella Ragonot, 1887; syn. (Vitula) 6007

edmandsae Heinrich, 1956; syn. emendation (Vitula) 6007

**serratilineella** Ragonot, 1887^358^, ^359^; valid (Vitula) 6007

bombylicolella (Amsel, 1955); syn. (Moodna) 6007

**pinei** Heinrich, 1956; valid (Vitula) 6009

**aegerella** Neunzig, 1990^360^; valid (Vitula) 4415

**insula** Neunzig, 1990^360^; valid (Vitula)

**coconinoana** Neunzig, 1990^360^; valid (Vitula) 4416

**setonella** (McDunnough, 1927); valid (Moodna) 6010

**broweri** (Heinrich, 1956); valid (Manhatta) 6011

**Volatica** Heinrich, 1956

**gallivorella** Neunzig, 1990^361^; valid (Volatica) 4425

**Caudellia** Dyar, 1904

**apyrella** Dyar, 1904; valid (Caudellia) 6012

albovittella Dyar, 1904^362^; syn. (Caudellia) 6013

**floridensis** Neunzig, 1990^363^; valid (Caudellia) 4431

**nigrella** (Hulst, 1890); valid (Ephestia) 6014 4427

arizonella (Walter, 1928); syn. (Ephestia) 6014 4427

**Sosipatra** Heinrich, 1956

**rileyella** (Ragonot, 1887); valid (Ephestia) 6015 4436

**anthophila** (Dyar, 1925); valid (Eurythmia) 6016 4437

**proximanthophila** Neunzig, 1990^364^; valid (Sosipatra)

**thurberiae** (Dyar, 1917); valid (Eurythmia) 6017 4438

**knudsoni** Neunzig, 1990^364^; valid (Sosipatra) 4439

**nonparilella** (Dyar, 1904); valid (Ephestia) 6018 4440

**Heinrichiessa** Neunzig, 1990^365^

**sanpetella** Neunzig, 1990; valid (Heinrichiessa)

**Ribua** Heinrich, 1940

**droozi** Neunzig, 1990^366^; valid (Ribua)

**innoxia** Heinrich, 1940^367^; valid (Ribua) 4445

**Bethulia** Ragonot, 1888

**championella** Ragonot, 1888^368^; valid (Bethulia) 4444

**Plodia** Guenée, 1845

**interpunctella** (Hübner, [1813]); valid (Tinea) 6019 4448

interpunctalis (Hübner, 1825); syn. (Tinea) 6019 4448

zeae (Fitch, 1856); syn. (Tinea) 6019 4448

castaneella Reutti, 1898; syn. (Plodia) 6019 4448

latercula (Hampson, 1901); syn. (Unadilla) 6019 4448

glycinivora (Matsumura, 1917); syn. (Ephestia) 6019 4448

**Ephestia** Guenée, 1845

Hyphantidium Scott, 1859

Anagasta Heinrich, 1956^369^

**kuehniella** Zeller, 1879; valid (Ephestia) 6020 4452

fuscofasciella Ragonot, 1887; syn. (Ephestia) 6020 4452

gitonella Druce, 1896; syn. (Ephestia) 6020 4452

ischnomorpha (Meyrick, 1931)^370^; syn. (Homoeosoma) 4452

alba Roesler, 1966;

syn. form infrasubspecific (Ephestia (Anagasta)) 4452

nigra Roesler, 1966;

syn. form infrasubspecific (Ephestia (Anagasta)) 4452

**columbiella** Neunzig, 1990^371^; valid (Ephestia)

**elutella** (Hübner, 1796); valid (Tinea) 6021 4451

aquella (Denis & Schiffermüller, 1775)^372^; syn. (Tinea) 4451

elutea (Haworth, [1811]); syn. emendation (Phycis) 6021 4451

semirufa (Haworth, [1811]); syn. (Phycis) 6021 4451

angusta (Haworth, [1811]); syn. (Phycis) 6021 4451

rufa (Haworth, [1811]); syn. (Phycis) 6021 4451

sericarium (Scott, 1859); syn. (Hyphantidium) 6021 4451

roxburghii Gregson, 1871; syn. (Ephestia) 6021 4451

infumatella Ragonot, 1887; syn. (Ephestia) 6021 4451

affusella (Ragonot, 1888); syn. (Homoeosoma) 6021 4451

icosiella Ragonot, 1888; syn. (Ephestia) 6021 4451

amarella Dyar, 1904; syn. (Ephestia) 6021 4451

uniformata Dufrane, 1942;

syn. infrasubspecific, aberration (Ephestia) 6021 4451

pterogrisella Roesler, 1965^373^; syn. (Ephestia) 4451

**Uncitruncata** Neunzig, 2000^374^

**leuschneri** Neunzig, 2000; valid (Uncitruncata)

**Cadra** Walker, 1864

Xenephestia Gozmány, 1958

**cautella** (Walker, 1863); valid (Pempelia) 6022 4453

defectella Walker, 1864; syn. (Cadra) 6022 4453

desuetella (Walker, 1866); syn. (Nephopteryx) 6022 4453

passulella (Barrett, 1875); syn. (Ephestia) 6022 4453

formosella (Wileman & South, 1918);

syn. (Cryptoblabes) 6022 4453

rotundatella (Turati, 1930); syn. (Ephestia) 6022 4453

pelopis (Turner, 1947); syn. (Ephestia) 4453

irakella (Amsel, 1959); syn. (Ephestia) 6022 4453

**figulilella** (Gregson, 1871); valid (Ephestia) 6023 4454

ficulella (Barrett, 1875); syn. emendation (Ephestia) 6023 4454

milleri (Zeller, [1876]); syn. (Ephestia) 6023 4454

gypsella (Ragonot, 1887); syn. (Ephestia) 6023 4454

venosella (Turati, 1926); syn. (Ephestia) 6023 4454

ernestinella (Turati, 1927); syn. (Ephestia) 6023 4454

halfaella Roesler, 1966;

syn. form infrasubspecific (Cadra) 4454

**Bandera** Ragonot, 1887

Nasutes Hampson, 1930

**binotella** (Zeller, 1872); valid (Anerastia) 6024 4455

subluteella Ragonot, 1887; syn. (Bandera) 6024 4455

**cupidinella** Hulst, 1888; valid (Bandera) 6025 4456

conspersella (Ragonot, 1901); syn. (Anerastia) 6025 4456

venata (Hampson, 1930); syn. (Nasutes) 6025 4456

**virginella** Dyar, 1908; valid (Bandera) 6026 4457

**Wakulla** Shaffer, 1968

**carneella** (Barnes & McDunnough, 1913); valid (Bandera) 6027 4458

**Tampa** Ragonot, 1887

**dimediatella** Ragonot, 1887; valid (Tampa) 6028 4459

**Varneria** Dyar, 1904

**postremella** Dyar, 1904; valid (Varneria) 6029

**atrifasciella** Barnes & McDunnough, 1913; valid (Varneria) 6030 4463

**Eurythmia** Ragonot, 1887

**hospitella** (Zeller, [1876]); valid (Ephestia) 6031 4464

**angulella** Ely, 1910; valid (Eurythmia) 6032

diffusella Ely, 1910; syn. (Eurythmia) 6032

**furnella** Ely, 1910^375^; valid (Eurythmia) 6033

**spaldingella** Dyar, 1905^376^; valid (Eurythmia) 6031

**yavapaella** Dyar, 1906^376^; valid (Eurythmia) 6031 4465

**Erelieva** Heinrich, 1956

**quantulella** (Hulst, 1887); valid (Pempelia) 6034 4466

santiagella (Dyar, 1919); syn. (Eurythmia) 6034 4466

**parvulella** (Ely, 1910); valid (Eurythmia) 6035

**Barberia** Dyar, 1905

**affinitella** Dyar, 1905; valid (Barberia) 6036 4469

**Bema** Dyar, 1914

Relmis Dyar, 1914

**neuricella** (Zeller, 1848)^377^; valid (Ephestia) 4261

myja Dyar, 1914; syn. emendation (Bema) 4261

**Cabnia** Dyar, 1904

**myronella** Dyar, 1904; valid (Cabnia) 6037

**Anerastia** Hübner, 1825

Prinanerastia Hampson, 1918^5^

**lotella** (Hübner, 1813); valid (Tinea) 6038

miniosella (Zincken, 1818); syn. (Phycis) 6038

**Coenochroa** Ragonot, 1887

Petaluma Hulst, 1888

Alamosa Hampson, 1901

**californiella** Ragonot, 1887; valid (Coenochroa) 6039 4472

inspergella Ragonot, 1887; syn. (Coenochroa) 6039 4472

monomacula Dyar, 1914; syn. (Coenochroa) 4472

miasticta (Hampson, 1918); syn. (Metacrateria) 4472

**illibella** (Hulst, 1887); valid (Anerastia) 6040 4473

puricostella Ragonot, 1887; syn. (Coenochroa) 6040 4473

piperatella (Hampson, 1901); syn. (Alamosa) 6040 4473

**bipunctella** (Barnes & McDunnough, 1913); valid (Alamosa) 6041 4474

**Peoria** Ragonot, 1887

Aurora Ragonot, 1887

Statina Ragonot, 1887

Ceara Ragonot, 1888

Calera Ragonot, 1888

Altoona Hulst, 1888

Cayuga Hulst, 1888

Volusia Hulst, 1890; preocc. by Robineau-Desvoidy, 1830

Wekiva Hulst, 1890

Osceola Hulst, 1891; nomen nudum

Chipeta Hulst, 1892

Trivolusia Dyar, [1903]; repl. name

Ollia Dyar, 1904

**longipalpella** (Ragonot, 1887); valid (Aurora) 6042

**bipartitella** Ragonot, 1887; valid (Peoria) 6043

roseopennella (Hulst, 1890); syn. (Volusia) 6043

**tetradella** (Zeller, 1872); valid (Anerastia) 6044 4550

**opacella** (Hulst, 1887); valid (Anerastia) 6045 4544

dichroeella (Ragonot, 1889); syn. (Saluria) 6045 4544

**floridella** Shaffer, 1968; valid (Peoria) 6046

**padreella** A. Blanchard, 1980^378^; valid (Peoria) 4545

**rostrella** (Ragonot, 1887); valid (Saluria) 6047

**gemmatella** (Hulst, 1887); valid (Spermatophthora) 6048 4541

bistriatella (Hulst, 1890); syn. (Cayuga) 6048 4541

pamponerella (Dyar, 1908); syn. (Pectinigera) 6048 4541

**roseotinctella** (Ragonot, 1887); valid (Statina) 6049 4547

punctilimbella (Ragonot, 1888); syn. (Calera) 6049 4547

bifasciella (Hampson, 1901); syn. (Statina) 6049 4547

**johnstoni** Shaffer, 1968; valid (Peoria) 6050 4543

**santaritella** (Dyar, 1904); valid (Ollia) 6051 4548

**holoponerella** (Dyar, 1908); valid (Ollia) 6052 4542

**approximella** (Walker, 1866); valid (Eurhodope) 6053

haematica (Zeller, 1872); syn. (Anerastia) 6053

roseatella (Packard, 1873); syn. (Nephopteryx) 6053

cremoricosta (Hampson, 1918); syn. (Hypsotropa) 6053

**luteicostella** (Ragonot, 1887); valid (Hypsotropa) 6054

nodosella (Hulst, 1890); syn. (Wekiva) 6054

perlepidellus (Smith, 1891);

syn. nomen nudum (Osceola)

perlepidella (Hulst, 1892); syn. (Chipeta) 6054

**punctata** Shaffer, 1976; valid (Peoria) 6055

**insularis** Shaffer, 2003^379^; valid (Peoria)

**gaudiella** (Hulst, 1890)^29^; valid (Statina) 6056

**Tolima** Ragonot, 1888

**cincaidella** Dyar, 1904^29^; valid (Tolima) 6057

**Navasota** Ragonot, 1887

**hebetella** Ragonot, 1887^29^; valid (Navasota) 6058

**Anacostia** Shaffer, 1968

**tribulella** Shaffer, 1968; valid (Anacostia) 6059

**Arivaca** Shaffer, 1968

**pimella** (Dyar, 1906); valid (Poujadia) 6060 4509

**linella** Shaffer, 1968; valid (Arivaca) 6061

**ostreella** (Ragonot, 1887); valid (Saluria) 6062 4507

discostrigella (Dyar, 1904); syn. (Peoria) 6062 4507

**poohella** Shaffer, 1968; valid (Arivaca) 6063 4508

**albidella** (Hulst, [1901]); valid (Peoria) 6064 4505

**artella** Shaffer, 1968; valid (Arivaca) 6065 4506

**albicostella** (Grossbeck, 1917); valid (Calera) 6066 4504

**Atascosa** Hulst, 1890

Eumoorea Dyar, 1917

**glareosella** (Zeller, 1872); valid (Anerastia) 6067 4511

bicolorella Hulst, 1890; syn. (Atascosa) 6067 4511

albocostella (Hulst, 1900); syn. (Maricopa) 6067 4511

chejelis (Dyar, 1919); syn. (Poujadia) 4511

**heitzmani** Shaffer, 1980^380^; valid (Atascosa)

**Homosassa** Hulst, 1890

**ella** (Hulst, 1887); valid (Ephestia) 6068 4534

**platella** Shaffer, 1968; valid (Homosassa) 6069 4535

**incudella** Shaffer, 1968; valid (Homosassa) 6070

**blanchardi** Shaffer, 1976; valid (Homosassa) 6071

**Reynosa** Shaffer, 1968

**floscella** (Hulst, 1890); valid (Atascosa) 6072 4551

**Goya** Ragonot, 1888

Atopothoures A. Blanchard, 1975

**stictella** (Hampson, 1918); valid (Saluria) 6073 4533

**ovaliger** (A. Blanchard, 1975); valid (Atopothoures) 6074 4531

**Uinta** Hulst, 1888^29^

**oreadella** Hulst, 1888; valid (Uinta) 6075

**Epipaschiinae**

**Macalla** Walker, [1859]

Aradrapha Walker, [1866]; preocc. by Walker, [1866]

Mochlocera Grote, 1876

Pseudomacalla Dognin, 1908

**thyrsisalis** Walker, [1859]; valid (Macalla) 5575 3771

mixtalis (Walker, [1866]); syn. (Aradrapha) 5575 3771

**phaeobasalis** Hampson, 1916; valid (Macalla) 5576 3769

**zelleri** (Grote, 1876)^381^; valid (Mochlocera) 5579 3772

**Epipaschia** Clemens, 1860

**superatalis** Clemens, 1860; valid (Epipaschia) 5577

borealis (Grote, 1870); syn. (Deuterolyta) 5577

olivalis (Hulst, 1886); syn. (Tetralopha) 5577

**Cacozelia** Grote, 1878

**pemphusalis** (Druce, 1899); valid (Pococera) 3658

albomedialis (Barnes & Benjamin, 1924)^382^;

syn. (Epipaschia) 5578 3658

**basiochrealis** Grote, 1878; valid (Cacozelia) 5580 3655

**interruptella** (Ragonot, 1888)^383^; valid (Epipaschia) 5586 3657

dentilineella (Hulst, 1900); syn. (Jocara (Toripalpus)) 5586 3657

**elegans** (Schaus, 1912)^383^; valid (Pococera) 5587 3656

neotropica (Amsel, 1956)^384^; syn. (Tioga) 3656

**Milgithea** Schaus, 1922

**alboplagialis** (Dyar, 1905)^385^; valid (Cacozelia) 5581 3792

**trilinearis** (Hampson, 1906)^386^; valid (Jocara) 3796

**Toripalpus** Grote, 1878^387^

Yuma Hulst, 1889^388^

**breviornatalis** Grote, 1878; valid (Toripalpus) 5584 3886

**trabalis** Grote, 1881; valid (Toripalpus) 5585 3887

adulatalis Hulst, 1887^389^; syn. (Toripalpus) 5585 3887

**Deuterollyta** Lederer, 1863^390^

Winona Hulst, 1888

Oedomia Dognin, 1906

Ajocara Schaus, 1925

Ajacania Schaus, 1925

**majuscula** Herrich-Schäffer, 1871; valid (Deuterollyta) 3725

incrustalis (Hulst, 1887); syn. (Toripalpus) 5582 3725

infectalis Möschler, 1890; syn. (Deuterollyta) 3725

ferrifusalis (Hampson, 1906); syn. (Jocara) 3725

obscuralis (Schaus, 1912); syn. (Jocara) 3725

perseella (Barnes & McDunnough, 1913)^391^; syn. (Jocara) 5583 3725

musettalis (Schaus, 1934); syn. (Jocara) 3725

**Oneida** Hulst, 1889

**lunulalis** (Hulst, 1887); valid (Toripalpus) 5588

**luniferella** Hulst, 1895; valid (Oneida) 5589 3799

diploa Dyar, 1920^392^; syn. (Oneida) 3799

pallidalis Barnes & Benjamin, 1924^390^;

syn. infrasubspecific? (Oneida) 5589 3799

**grisiella** Solis, 1991^393^; valid (Oneida)

**Pococera** Zeller, 1848

Tetralopha Zeller, 1848^394^

Lanthaphe Clemens, 1860

Hemimatia Lederer, 1863

Benta Walker, 1863

Auradisa Walker, [1866]

Saluda Hulst, 1888

Katona Hulst, 1888

Loma Hulst, 1888

Wanda Hulst, 1888

Tioga Hulst, 1888

Attacapa Hulst, 1889

Afra Ghesquière, 1942^5^

**robustella** (Zeller, 1848); valid (Tetralopha) 5595

diluculella (Grote, 1880); syn. (Tetralopha) 5595

**scortealis** (Lederer, 1863); valid (Hemimatia) 5596 3847

slossi (Hulst, 1895); syn. (Benta) 5596 3847

**melanogrammos** (Zeller, 1872); valid (Tetralopha) 5597 3842

**texanella** Ragonot, 1888; valid (Pococera) 5598 3850

**callipeplella** (Hulst, 1888); valid (Tetralopha) 5599 3828

**speciosella** (Hulst, 1900); valid (Benta) 5600 3848

**floridella** (Hulst, 1900); valid (Benta) 5601

**subcanalis** (Walker, 1863); valid (Nephopteryx) 5602 3849

taleolalis (Hulst, 1886); syn. (Toripalpus) 5602 3849

querciella (Barnes & McDunnough, 1913);

syn. (Tetralopha) 5602 3849

**maritimalis** (McDunnough, 1939); valid (Tetralopha) 5603

**militella** (Zeller, 1848); valid (Tetralopha) 5604

platanella (Clemens, 1860); syn. (Lanthaphe) 5604

**aplastella** (Hulst, 1888); valid (Tioga) 5605 3824

**asperatella** (Clemens, 1860); valid (Lanthaphe) 5606

**vacciniivora** (Munroe, 1963); valid (Tetralopha) 5607

**expandens** (Walker, 1863); valid (Benta) 5608

nephelotella (Hulst, 1888); syn. (Loma) 5608

clemensalis (Dyar, 1904);

syn. infrasubspecific (Tetralopha) 5608

**spaldingella** (Barnes & Benjamin, 1924); valid (Tetralopha) 5609

**dolorosella** (Barnes & Benjamin, 1924); valid (Tetralopha) 5610

**provoella** (Barnes & Benjamin, 1924); valid (Tetralopha) 5611

**arizonella** (Barnes & Benjamin, 1924); valid (Tetralopha) 5612 3825

**thoracicella** (Barnes & Benjamin, 1924); valid (Tetralopha) 5613 3851

**griseella** (Barnes & Benjamin, 1924); valid (Tetralopha) 5614 3835

**fuscolotella** (Ragonot, 1888); valid (Tetralopha) 5615 3832

nigricans (Hulst, 1900)^395^; **new synonym** (Salebria) 5778

**tiltella** (Hulst, 1888); valid (Wanda) 5616 3852

**humerella** (Ragonot, 1888); valid (Tetralopha) 5617 3839

formosella (Hulst, 1900); syn. (Tetralopha) 5617 3839

**gelidalis** (Walker, [1866]); valid (Auradisa) 3833

subalbella (Walker, 1866); syn. (Myelois) 3833

tertiella Dyar, 1905^396^; syn. (Pococera) 5618 3833

irrorata Schaus, 1912; syn. (Pococera) 3833

**baptisiella** (Fernald, 1887); valid (Tetralopha) 5619

**euphemella** (Hulst, 1888); valid (Katona) 5620 3831

variella Ragonot, 1888; syn. (Pococera) 5620 3831

melanographella Ragonot, 1888; syn. (Pococera) 5620 3831

**Tallula** Hulst, 1888

**atrifascialis** (Hulst, 1886); valid (Tetralopha) 5591 3866

**watsoni** Barnes & McDunnough, 1917; valid (Tallula) 5592

**baboquivarialis** Barnes & Benjamin, 1926; valid (Tallula) 5593 3867

**beroella** Schaus, 1912^397^; valid (Tallula) 3868

bunniotis (Dyar, 1913); syn. (Tioga) 3868

**fieldi** Barnes & McDunnough, 1913; valid (Tallula) 5594 3869

**Phidotricha** Ragonot, [1889]^398^

Eutrichocera Hampson, 1904

Jocarula Dyar, 1925

**erigens** Ragonot, [1889]; valid (Phidotricha) 5590 3816

dryospila (Meyrick, 1936); syn. (Auradisa) 3816

**Incertae sedis**

**glastianalis** Schaus, 1922^399^; valid (Macalla) 3897

**Pyralinae**

**Pyralis** Linnaeus, 1758

Aletes Rafinesque, 1815; nomen nudum

Ceropsina Rafinesque, 1815; nomen nudum

Spyrella Rafinesque, 1815; nomen nudum

Asopia Treitschke, 1828

Sacatia Walker, 1863

Eutrichodes Warren, 1891

**farinalis** Linnaeus, 1758^400^; valid (Pyralis) 5510 3199

erecta (Fourcroy & Geoffroy, 1785); syn. (Phalaena) 3199

farinatus (Haworth, 1803); syn. (Crambus) 3199

domesticalis (Zeller, 1847); syn. (Asopia) 3199

fraterna Butler, 1879; syn. (Pyralis) 5510 3199

tenerifensis Rebel, 1906; syn. variety, aberration (Pyralis) 3199

infumata Rebel, 1940;

syn. aberration infrasubspecific (Pyralis) 3199

**a. meridionalis** Schmidt, 1934; ssp. extralimital (Pyralis) 5510 3199

**b. orientalis** Amsel, 1961; ssp. extralimital (Pyralis) 5510 3199

**manihotalis** Guenée, 1854^400^; valid (Pyralis) 5515 3200

vetusalis Walker, [1859]; syn. (Pyralis) 5515 3200

gerontesalis Walker, [1859]; syn. (Pyralis) 5515 3200

laudatella (Walker, 1863); syn. (Sacatia) 5515 3200

despectalis Walker, [1866]; syn. (Pyralis) 5515 3200

miseralis Walker, [1866]; syn. (Pyralis?) 5515 3200

achatina Butler, 1877; syn. (Pyralis) 5515 3200

haematinalis (Saalmüller, 1880); syn. (Asopia) 3200

gerontialis (Meyrick, 1888); syn. emendation (Asopia) 5515 3200

centripuctalis (Gaede, 1916); syn. (Endotricha) 3200

pupalis Strand, 1919; syn. (Pyralis) 3200

compsobathra Meyrick, 1932; syn. (Pyralis) 3200

**Aglossa** Latreille, [1796]

Euclita Hübner, [1825]^401^

Oryctocera Ragonot, 1891^402^

Crocalia Ragonot, 1892^402^

Agriope Ragonot, 1894^402^

**costiferalis** (Walker, 1866)^403^; valid repl. name (Pyralis) 5511 3160

costigeralis (Walker, [1865]);

syn. preocc. by Walker, 1862 (Pyralis) 5511 3160

**disciferalis** (Dyar, 1908)^403^; valid (Pyralis) 5512 3162

**electalis** Hulst, 1886^403^; valid (Aglossa) 5513 3163

**cacamica** (Dyar, 1914)^403^; valid (Pyralis) 5514 3158

**pinguinalis** (Linnaeus, 1758)^404^; valid (Phalaena (Pyralis)) 5516 3167

marmorella (Goeze, 1783); syn. (Phalaena) 3167

pinguedinis (Retzius, 1783); syn. (Phalaena) 3167

marmoratella (Villers, 1789); syn. (Phalaena) 3167

pinguis (Fabricius, 1799); syn. (Crambus) 3167

pinguiculatus (Haworth, 1809); syn. (Crambus) 3167

streatfieldii Curtis, 1833; syn. (Aglossa) 5516 3167

guicciardii Constantinio, 1922; syn. (Aglossa) 3167

maroccana Schmidt, 1934; syn. (Aglossa) 3167

**caprealis** (Hübner, [1809])^404^; valid (Pyralis) 5517 3159

capreolatus (Haworth, 1811)^5^; syn. (Crambus) 5517 3159

aenalis (Costa, 1836); syn. (Pyralis) 3159

domalis Guenée, 1854; syn. (Aglossa) 5517 3159

incultella (Walker, 1866); syn. (Acrobasis) 3159

euthealis (Hulst, 1886); syn. (Tetralopha) 5517 3159

**cuprina** (Zeller, 1872); valid (Pyralis) 5518 3161

**acallalis** Dyar, 1908; valid (Aglossa) 5519 3156

**baba** Dyar, 1914; valid (Aglossa) 5520 3157

**gigantalis** Barnes & Benjamin, 1925; valid (Aglossa) 5521 3165

**furva** Heinrich, 1931; valid (Aglossa) 5522 3164

**oculalis** Hampson, 1906; valid (Aglossa) 5523 3166

**Hypsopygia** Hübner, [1825]

Dolichomia Ragonot, 1891^405^

Herculia Walker, 1859^405^

Cisse Walker, 1863^405^

Buzala Walker, 1863^405^

Ocrasa Walker, [1866]^405^

Bejuda Walker, 1866^405^

Pseudasopia Grote, 1873^405^

Bleone Ragonot, 1890^406^

Orthopygia Ragonot, 1890^405^

Parasopia Möschler, 1890^407^

**costalis** (Fabricius, 1775); valid (Phalaena) 5524 3187

fimbrialis (Denis & Schiffermüller, 1775);

syn. (Pyralis) 5524 3187

purpurana (Thunberg, 1784); syn. (Tortrix) 3187

hyllalis (Walker, 1859); syn. (Pyralis) 5524 3187

rubrocilialis (Staudinger, 1870);

syn. aberration infrasubspecific (Asopia) 5524

**a. syriaca** Zerny, 1914^5^; ssp. extralimital (Hypsopygia)

**planalis** (Grote, 1880); valid **comb. n.** (Asopia) 5525 3182

anniculalis (Hulst, 1886); syn. (Asopia) 5525 3182

occidentalis (Hulst, 1886); syn. (Asopia) 5525 3182

**intermedialis** (Walker, 1862)^408^; valid (Pyralis) 5526 3197

sodalis (Walker, 1869); syn. (Pyralis) 5526 3197

squamealis (Grote, 1873); syn. (Pseudasopia) 5526 3197

**phoezalis** (Dyar, 1908); valid **comb. n.** (Herculia) 5527 3198

**cohortalis** (Grote, 1878); valid **comb. n.** (Asopia) 5528 3196

florencealis (Blackmore, 1920); syn. (Herculia) 5528 3196

**thymetusalis** (Walker, 1859); valid **comb. n.** (Botys) 5529

devialis (Grote, 1875); syn. (Asopia) 5529

**binodulalis** (Zeller, 1872)^408^; valid (Asopia) 5530 3173

**nostralis** (Guenée, 1854)^408^, ^409^; valid (Pyralis) 3194

helenensis (Wollaston, 1879); syn. (Pyralis) 3194

tenuis (Butler, 1880); syn. (Pyralis) 3194

dissimilalis (Möschler, 1890)^410^; syn. (Parasopia) 3194

sordidalis (Barnes & McDunnough, 1913)^410^;

syn. (Herculia) 5531 3194

psammioxantha (Dyar, 1917)^410^; syn. (Herculia) 3194

venezuelensis (Amsel, 1956)^410^; syn. (Herculia) 3194

**olinalis** (Guenée, 1854)^408^; valid (Pyralis) 5533 3180

trentonalis (Lederer, 1863); syn. (Asopia) 5533 3180

himonialis (Zeller, 1872); syn. (Asopia) 5533 3180

infimbrialis (Dyar, 1908)^411^; syn. (Herculia) 5532 3180

**Arispe** Ragonot, 1891

Uscodys Dyar, 1908^412^

**cestalis** (Hulst, 1886); valid (Anerastia) 5534 3168

atalis (Dyar, 1908)^413^; syn. (Uscodys) 5535 3168

**Neodavisia** Barnes & McDunnough, 1914; repl. name

Davisia Barnes & McDunnough, 1913;

preocc. by Del Guercio, 1909

**singularis** (Barnes & McDunnough, 1913); valid (Davisia) 5536 3193

**melusina** Ferguson, A. Blanchard & Knudson, 1984^414^;

valid (Neodavisia) 3192

### Species not included or removed from the North American list

**Musotiminae**

**Austromusotima** Solis & Yen, 2004

**camptozonale** (Hampson, 1897)^415^;

valid introduced not established (Oligostigma)

**Chrysauginae**

**Blepharocerus** Blanchard, 1852^416^

**rosellus** Blanchard, 1852; valid (Blepharocerus) 5572 3254

rufulalis (Lederer, 1863); syn. (Asopia) 5572 3254

**ignitalis** Hampson, 1906; valid (Blepharocerus) 5573 3253

**Galleriinae**

**Aphomia** Hübner, [1825]

**fuscolimbella** (Ragonot, 1887)^417^; valid (Melissoblaptes) 5631

**Phycitinae**

**Crocidomera** Zeller, 1848

**turbidella** Zeller, 1848^232^; valid (Crocidomera) 5706 3949

**Cryptoblabes** Zeller, 1848

Albinia Briosi, 1877; preocc. by Robineau-Desvoidy, 1830

**gnidiella** (Millière, 1867)^418^; valid (Ephestia) 3921

aliena Swezey, 1909; syn. (Cryptoblabes) 3921

**Apomyelois** Heinrich, 1956

Ectomyelois Heinrich, 1956^236^

Spectrobates Roesler, 1956 (not Meyrick, 1935)

**decolor** (Zeller, 1881)^419^; valid (Myelois) 5722 3965

ephestiella (Hampson, 1901); syn. (Nephopteryx) 5722 3965

**Creobota** Turner, 1931

**grossipunctella** (Ragonot, 1888)^420^;

valid, introduction not established (Myelois) 5715

coccophthora Turner, 1931^421^; syn. (Creobota)

**Phycitodes** Hampson, 1917

Rotruda Heinrich, 1956

**albatella** (Ragonot, 1887)^325^; valid (Homoeosoma) 5946 4295

parvum (Gerasimov, 1930); syn. (Homoeosoma) 5946 4295

pseudonimbella (Bentinck, 1937)^422^; syn. (Homoeosoma)

dierli Roessler, 1973; syn. (Phycitodes)

**Cactobrosis** Dyar, 1914

**longipennella** (Hampson, 1901)^423^; valid (Euzophera) 5990 4367

elongatella (Hampson, 1901); syn. (Moodna) 5990 4367

**Vitula** Ragonot, 1887

Hornigia Ragonot, 1887; preocc. by Ragonot, 1885

Manhatta Hulst, 1890

**biviella** (Zeller, 1848); valid (Ephestia)

lugubrella Ragonot, 1887^424^; syn. (Vitula) 6008

**Epipaschiinae**

**Coenodomus** Walsingham, 1888^425^

Dyaria Neumoegen, 1893

Alippa Aurivillius, 1894

**hockingi** Walsingham, 1888; valid (Coenodomus) 5621

singularis (Neumoegen, 1893); syn. (Dyaria) 5621

anomala (Aurivillius, 1894); syn. (Alippa) 5621

**Tallula** Hulst, 1888

**atramentalis** (Lederer, 1863)^426^, ^427^; valid (Hemimatia) 5590 3865

## Notes

1 Sarabotys cupreicostalis is here reported for the first time in the United States from Arizona by MAS. Five specimens are deposited in the NMNH (National Museum of Natural History, Washington, DC): Babquivari Mts., Pima Co., 1–15 Sept. 1923 (2 specimens); Huachuca Mts., Aug. 16–23, Barnes Coll. (1 specimen); and Huachuca Mts., Miller Canyon, 5000’, Cochise Co., 18 July 1974, J. G. Franclemont (2 specimens).

2 Sclerocona acutellus was first reported in the United States from Connecticut, Massachusetts, Maryland, and Rhode Island by Wagner et al. (2003).

3 Leraut (2012) recognized sinensellus as a subspecies.

4 Pseudopyrausta marginalis was first reported in the United States from Texas by Bordelon and Knudson (2010) in the 2009 Season Summary and discussed again in Bordelon and Knudson (2011).

5 These names were recognized as synonyms by Nuss et al. (2003–2015).

6 Many new synonyms and several subspecies were recognized in Munroe (1995a,b). If not specifically footnoted, this is true in all cases where we do not list a MONA #, but do list an NTCL #.

7 An expanded concept of Anania is recognized as in Leraut (2005a) and Tränker et al. (2009).

8 Mutuuraia and Nealgedonia were synonymized with Algedonia in Maes (2005).

9 Anania tertialis and plectilis were elevated to species status in Yang et al. (2012).

10 Anania tennesseensis was described in Yang et al. (2012).

11 Aponia aponianalis was first reported in the United States from Texas, by Bordelon (2011) in the 2010 Season Summary and discussed in Bordelon and Knudson (2011).

12 Dosara was synonymized with Achyra in Maes (1987).

13 Sitochroa palealis was first reported in the United States from Illinois, Indiana, Michigan, and Wisconsin by Passoa et al. (2008).

14 Paracorsia repandalis is here reported for the first time in the United States from Indiana by Jim Vargo. Specimens at the FMNH (Florida Museum of Natural History): Mishawaka, 41°37’46.2”N -86°08’13.9”, St. Joseph Co. on 11 Aug 2010 and again on 14 Aug 2012. Identification by James Hayden.

15 Leimonia was synonymized with Loxostege in Maes (1994).

16 Meridiophila was synonymized with Loxostege in Leraut (2005a).

17 Proteroeca was synonymized with Pyrausta in Maes (1994).

18 Mardinia was synonymized with Pyrausta in Maes (2002).

19 Pyrausta cardinalis was first reported in the United States from Florida by Hayden et al. (2011).

20 Pyrausta insequalis replaced subsequalis as the accepted name in Munroe (1995a).

21 Pyrausta borealis was recognized as a distinct species in Poole and Lewis (1996).

22 Daulia was moved to the Pyraustinae by Nuss et al. (2003–2015) based on structure of the genitalia and tympanum.

23 Ecpyrrhorrhoe puralis was first reported in the United States from Alabama, Georgia, Kentucky, Maryland, Mississippip, Pennsylvania, South Carolina, Tennnessee, and West Virginia by Solis et al. (2010).

24 Spilomelinae was recognized as a subfamily in Minet [1982], Solis and Maes (2002), and Regier et al. (2012).

25 Phaedropsis principialis was recognized as a new combination in Munroe (1995a).

26 Framinghamia was synonymized with Phlyctaenia (Maes 1994), and then subsequently with Anania (Leraut 2005a) in the Pyraustinae, but Hayden (pers. comm.) places it back in the Spilomelinae as in Munroe (1983) based on the morphology of the genitalia.

27 Hyperthalia was synonymized with Lygropia in Vári, Kroon and Krüger (2002).

28 Lygropia fusalis was first reported in the United States by Munroe (1995a) through synonymy of Nacoleia hampsoni.

29 This species is indicated as misplaced in Munroe (1983).

30 This species is indicated as misplaced in Munroe (1995a).

31 Lygropia memmialis was recognized as a species in Munroe (1995a), but its new status was not indicated.

32 Cyclocena was synonymized with Microthyris in Hayden and Dickel (2014).

33 Microthyris lelex was first reported in the United States by Hayden and Dickel (2014).

34 Pantographa suffusalis was first reported in the United States in Texas by Bordelon (2009) and discussed in Bordelon and Knudson (2009).

35 Pleuroptya penumbralis was synonymized with Pleuroptya silicalis in Munroe (1995a).

36 Herpetogramma ipomoealis was synonymized with Pleuroptya fluctuosalis (Lederer), which was transferred to Herpetogramma in Munroe (1995a).

37 Botys otreusalis was synonymized with Herpetogramma phaeopteralis in Vári, Kroon and Krüger (2002).

38 Acharana descripta was synonymized with Herpetogramma phaeopteralis in Solis (2010).

39 The identity of Herpetogramma centrostrigalis was confirmed in Solis (2010).

40 Herpetogramma sphingealis was described in Handfield and Handfield (2011).

41 Blepharomastix magualis, aplicalis, rehamalis, and differentialis were transferred to Hileithia in Munroe (1995a).

42 Hileithia decostalis was first reported in the United States from Mississippi by Mather and Munroe (1992).

43 Stenia pulchellalis was synonymized with Hydriris ornatalis in Viette (1990).

44 Ercta ornatalis orientalis was synonymized with Ercta ornatalis ornatalis in Inoue (1982).

45 Lineodes triangulalis was reported in the United States from Florida and Texas by Hayden and Dickel (2014), although it had been previously reported from Florida by Dyar (1901).

46 Lineodes elcodes was first reported in the United States from California by Epstein (2004).

47 Lineodes multisignalis was first reported in the United States from Florida by Covell and Hayden (2014).

48 Lineodes vulnifica was first reported in the United States by Annand (1945) (Hayden pers comm.).

49 Zellerina (replacement name for Stenoptycha) was synonymized with Atomopteryx in Munroe (1995a).

50 Lamprosema baracoalis was first reported in the United States from Texas by Blanchard and Knudson (1985a).

51 Neoleucinodes torvis was first reported in the United States from Florida by Dickel (1985) and discussed in Hayden and Dickel (2014).

52 Udea aenigmatica was transferred from Platytes and recognized as a distinct species by Landry (1991).

53 Desmia divisalis was synonymized with Desmia ufeus in Munroe (1995a), but its status as a new synonym was not indicated.

54 Desmia propinqualis was synonymized with Desmia ploralis in Munroe (1995a).

55 Blepharomastix eudamidasalis was transferred to Diasemiodes in Munroe (1995a).

56 Hymenia exodias was synonymized with Spoladea recurvalis in Zimmerman (1958).

57 Nacoleia charesalis was first reported in the United States from Florida by Hayden in Dixon and Anderson (2014a) and later discussed in Hayden and Troubridge (2015).

58 Duponchelia fovealis was first reported in the United States from California by Epstein (2004), and later discussed by Brambila and Stocks (2010) and Heppner and Brambila (2010).

59 Stemorrhages costata was transferred to Diaphania in Munroe (1995a), but its status as a new combination was not indicated; it was indicated as misplaced.

60 Dysallacta was synonymized with Glyphodes in Inoue (1982).

61 Glyphodes batesi was removed from synonymy with Glyphodes sibillalis by Nuss et al. (2003–2015). Comparison of the type specimen of Glyphodes batesi shows that it is a junior synonym of Glyphodes sibillalis and is included in this list.

62 Spelling corrected (misspelled in MONA as atlitalis); Glyphodes alitalis Hulst, 1886 (type locality USA) was synonymized with Glyphodes bivitralis Guenée, 1854 (from India) by Nuss et al. (2003–2015). After a comparison of a photo of the type of Glyphodes alitalis at the AMNH and the description of Glyphodes bivitralis we found that Munroe was correct in 1983 when he noted Glyphodes bivitralis as “authors, part, not Guenee 1854” so we exclude Glyphodes bivitralis from this list.

63 Glyphodes onychinalis was first reported in the United States from California by Solis (2008). It had been sighted and sent to MAS by Don Sterba who collected it in Culver City (Los Angeles Co.) as early as 2007; Ron Leuschner had seen it in his backyard in Manhattan Beach as early as 2006. Leuschner (2009) reported it from Los Angeles Co., Manhattan Beach.

64 Maruca vitrata was first reported in the United States from Florida by Dickel (1981).

65 Maruca testulalis, a pest of legume crops, was first synonymized with Maruca vitrata in Munroe (1995a), although not explicitly stated as a new synonym (Solis 2003).

66 The names fortificalis (Möschler, 1890) and pigralis (Dognin, 1909) listed in Munroe (1995a) were found to be synonyms of Omiodes metricalis (Möschler, 1881) by Gentili and Solis (1998) and are excluded here.

67 Omiodes exogrammalis was synonymized with Omiodes stigmosalis in Munroe (1995a).

68 Omiodes martyralis was first reported in the United States from Louisiana by Brou (1985) and discussed in Brou (1994).

69 Sylora was synonymized with Palpita in Shaffer et al. (1996).

70 Palpita persimilis was first reported in the United States from Florida by Hayden and Buss (2012).

71 Palpita euphaesalis was synonymized with Palpita illibalis in Munroe (1995a).

72 Palpita maritima was described in Sullivan and Solis (2013).

73 Polygrammodes oxydalis was recognized as a species distinct from Polygrammodes flavidalis in Munroe (1995a), but its new status was not indicated.

74 Loxomorpha and Maracayia were recognized as part of the Siga group and moved to near Laniifera by Landry, Roque-Albelo, and Hayden (2011).

75 Gonocausta sabinalis was omitted from Munroe (1983) but included in Munroe (1995a) from Texas.

76 Epipagis fenestralis was recognized as an unused senior synonym for Epipagis huronalis in Munroe (1995a) (see Note 143: 171 in Munroe (1995a) for a full explanation of the status of Epipagis fenestralis).

77 Nomophila triticalis was first reported in the United States from Florida by Heppner (2011) and discussed in Hayden and Dickel (2014).

78 Samea druchachalis was first reported in the United States from Texas by Bordelon and Knudson (2011).

79 Sameodes albiguttalis was transferred to Niphograpta in Munroe (1995a).

80 Somatania was recognized as a synonym of Crocidocnemis in Munroe (1995a).

81 Marasmia was synonymized with Cnaphalocrocis in Shaffer et al. (1996).

82 Bradina andresi was synonymized with Cnaphalocrocis trapezalis in Vári, Kroon and Krüger (2002).

83 Salbimorpha was synonymized with Salbia in Munroe (1995a).

84 Cangetta micralis was first reported in the United States from Florida by Hayden and Dickel (2014).

85 Eurrhyparodes splendens was first reported in the United States from Texas by Blanchard and Knudson (2007), but also reported as a new state record from Arizona by Bordelon (2007) in the same 2006 Season Summary.

86 Hydropionea was included in Munroe (1995a) under “Unplaced Genera”; its placement needs to be investigated further (Hayden pers. comm.).

87 Falcimorpha (replacement name for Falx) was synonymized with Microphysetica in Munroe (1995a).

88 Microphysetica philogelos was synonymized with Microphysetica hermeasalis in Munroe (1995a).

89 Pilocrocis glaucusalis was recognized as a species of Palpusia in Munroe (1995a).

90 Rhectocraspeda was recognized as the new generic name for Pilemia (preoccupied) in Munroe (1995a).

91 Araschnopsis was synonymized with Sisyracera in Munroe (1995a).

92 Sisyracera contortilinealis was first identified in the United States from Florida by Hayden (pers. comm.) based on a 2012 specimen from Monroe Co.

93 Plumipalpiella was established as a replacement name for Plumipalpia (preoccupied) in Munroe (1995a).

94 Frechinia murmuralis was recognized as a species in Munroe (1995a), but was not listed as new status.

95 Hercyna was synonymized with Metaxmeste in Fletcher and Nye (1984).

96 Chlorobaptella was established as a replacement name for Chlorobapta (preoccupied) in Munroe (1995a).

97 Metrea was synonymized with Cliniodes by Hayden (2011) and belongs in the Odontiinae (Munroe 1995a, Hayden 2011). Dichogamini was transferred to Glaphyriinae (see Note #100 below).

98 Basonga was synonymized with Cliniodes in Klima (1939).

99 Procliniodes was described as a subgenus of Cliniodes in Hayden (2011).

100 Dichogamini was transferred to Glaphyriinae in Munroe and Solis (1999).

101 Schacontia was transferred from Cybalomiinae to Glaphyriinae in Solis (2009).

102 Schacontia themis was described and first reported in the United States from Florida by Goldstein et al. (2013).

103 Schacontia rasa was first reported in the United States from Florida by Hayden in Dixon and Anderson (2015).

104 Ashwania was synonymized with Hellula in Shaffer et al. (1996).

105 Aethiophysa invisalis was recognized as the senior synonym of Aethiophysa lentiflualis in Munroe (1995a).

106 Cybalomia extorris was transferred to Aethiophysa in Solis (2009).

107 Ennomosia basalis was first reported in the United States from Florida by Hayden and Dickel (2014).

108 Lipocosma albinibasalis was established as a replacement name for Lipocosma albibasalis (preoccupied) in Munroe (1995a).

109 Evergestinae was synonymized with Glaphyriinae in Regier et al. (2012).

110 Maelinoptera was synonymized with Evergestis in Nuss (2005).

111 Reskovitsia was synonymized with Evergestis in Maes (1994).

112 Evergestis palousalis was recognized as the senior synonym of Evergestis obscuralis (preoccupied) in Munroe (1995a). Evergestis palousalis obscuralias was established as the replacement name for the subspecies previously recognized as Evergestis obscuralias obscuralis.

113 Evergestis dischematalis was established as the replacement name for Evergestis dimorphalis (preoccupied) in Munroe (1995a).

114 Speidel (1981) resurrected Musotiminae Meyrick, 1890 and synonymized Ambiini Munroe, 1972 which included Undulambia.

115 Neomusotima conspurcatalis was recognized as established due to biocontrol introduction in the United States by Boughton and Pemberton (2012). Ambia conspurcatalis was combined with Neomusotima in Solis et al. (2004).

116 Musotima fuscalis was synonymized with Neomusotima conspurcatalis in Solis et al. (2004).

117 Sineudonia was synonymized with Scoparia in Li et al. (2010).

118 Dipleurina, Malageudonia, and Vietteina were synonymized with Eudonia in Nuss (1999).

119 Eudonia alpina was recognized as the senior synonym of Eudonia lugubralis in Nuss (1999). The Old World synonyms not previously listed are included here.

120 Klots (1983) omitted Old World synonyms for species in the Crambinae. These are added here as in Nuss et al. (2003–2015).

121 The order of genera in the Crambinae follows Landry (1995).

122 Surattha santella and Surattha indentella were transferred to Prionapteryx by Bleszynski (1967). Landry (1995) retained these two species in Surattha as in Klots in Munroe (1983) and Munroe (1995a).

123 Loxophantis was synonymized with Prionapteryx in Bassi (2013).

124 Alloea was synonymized with Prionapteryx in Shaffer et al. (1996).

125 Spelling corrected; incorrectly spelled as huachucella in Klots (1983) and Munroe (1995a).

126 Fletcher and Nye (1984) referred Acigona to the Noctuidae. Agnew (1987) addressed the nomenclatural problem dealing with various crambine genera including Occidentalia. Munroe (1995a) mentioned that Occidentalia does not belong in the Haimbachiini. Landry (1995) listed Occidentalia as a separate genus in the Haimbachiini. None of the authors above explicitly indicated its new status as a new combination.

127 Eoreuma arenella was described in Blanchard and Knudson (1983a).

128 Diatraea mitteri was described in Solis et al. (2015).

129 All species in the United States formerly in the genus Vaxi were transferred to Argyria in Munroe (1995a). Argyria auratella and Argyria critica were not listed as new combinations, but Argyria tripsacas was. There were no changes in Landry (1995).

130 Argyria of the southeastern United States was reviewed by Martinez and Brown (2007).

131 Argyria argentana Martyn, 1797 is not the correct name for Argyria nummulalis because it is a nomen nudum according to Nuss et al. (2003–2015).

132 Pseudoancylolomia was synonymized with Euchromius and Pseudoancylolomia qadrii was synonymized with Euchromius ocellea in Schouten (1992).

133 This species was indicated as misplaced in Klots (1983).

134 Catoptria tristis was synonymized with Catoptria trichostomus in Sinev (2008).

135 Thaumatopsis bolterellus was transferred from Parapediasia in Landry (1995).

136 Thaumatopsis digramellus was first reported in the United States from Arizona by Landry (1995). Specimens were collected by O. C. Poling from Indian Oasis, Sells P.O., Pima Co. on 15–30 Apr 1922.

137 Fissicrambus albilineellus was transferred from Crambus in Landry (1995).

138 Fissicrambus quadrinotellus was transferred from Crambus in Bleszynski (1963, 1967), and also listed in Landry (1995).

139 Loxocrambus coloradellus was transferred from Crambus in Landry (1995).

140 Loxocrambus hospition was transferred from Fissicrambus in Landry (1993).

141 Neodactria was described as a new genus, and Crambus caliginosella, Crambus luteolella, Crambus modestella, Crambus murella, and Crambus zeella were all transferred to Neodactria in Landry (1995).

142 Neodactria glenni was described in Landry and Metzler (2002).

143 Neodactria daemonis and Neodactria oktibbeha were described in Landry and Brown (2005).

144 Neodactria cochisensis was described in Landry and Albu (2012).

145 Pseudopediasia was synonymized with Pediasia in Shaffer and Robinson (1987).

146 Oseriates was synonymized with Pediasia in Landry (1995).

147 Pseudopediasia mikkolai was synonymized with Pediasia aridella caradjaellus in Shaffer and Robinson (1987).

148 La cerveza was described in Landry (1995).

149 Parapediasia hulstellus was transferred from Crambus in Landry (1995).

150 Parapediasia ligonellus was first reported in the United States from Florida by Landry (1995). Specimens were collected by Jerry Powell from Key Largo, Monroe Co. on 2 Jun 1986.

151 Parapediasia torquatella was described in Landry (1995).

152 Almita was described as new genus, and Almita portalia and Almita texana as new species in Landry (1995).

153 Amphibolia was synonymized with Chrysoteuchia and recognized as a homonym of Macquart 1843 in Bleszynski (1965).

154 Veronese was synonymized with Chrysoteuchia in Bleszynski (1965).

155 Crambus pseudorostellus was synonymized with Crambus perlella in Leraut (2012).

156 Crambus monochromella was recognized as a subspecies in Karsholt (1996).

157 This species was listed as Incertae sedis by Landry (1995).

158 All Scirpophaga in the United States were transferred to Carectocultus in Becker and Solis (1990).

159 Carectocultus repugnatalis was synonymized with Carectocultus perstrialis in Solis and Becker [2003].

160 All Patissa in the United States were transferred to Leptosteges in Munroe (1995a).

161 Donacaula was revised by Martinez (2010) in an unpublished dissertation, therefore the new scientific names are not available until they are published.

162 Lathrotelinae was elevated to subfamily to include the genus Sufetula, closely related to the Acentropinae by Minet (2015).

163 Perforadix was synonymized with Sufetula in Solis and Shaffer (1999).

164 Sufetula carbonalis was described in Hayden (2013).

165 Although Solis (1999) proposed precedence of Nymphulinae over Acentropinae, the ICZN (2003) committee voted to maintain Acentropinae as the valid name.

166 Oligostigmoides cryptalis was the original spelling; Oligostigmoides cryptale was an unjustified emendation (Hampson 1897).

167 Synclita was synonymized with Elophila in Speidel (2005).

168 Munroessa was synonymized with Elophila in Yoshiyasu (1985).

169 Nymphula ekthlipsis was transferred to Elophila by Nuss et al. (2003–2015).

170 Elophila (Munroessa) serralinealis was no longer recognized as a subspecies in Munroe (1995a).

171 Hydreuretis and Cosmophylla were synonymized with Parapoynx in Shaffer et al. (1996).

172 Microdracon was synonymized with Parapoynx in Munroe (1992).

173 Parapoynx itealis was no longer recognized as a subspecies in Munroe (1995a).

174 Paraponyx dicentra was synonymized with Paraponyx diminutalis in Shaffer et al. (1996).

175 Nymphula uxorialis was synonymized with Parapoynx diminutalis in Speidel and Mey (1999).

176 Chrysendeton nigrescens was described in Heppner (1991).

177 Argyractis drumalis was indicated as a new combination by Habeck and Solis (1994).

178 Petrophila heppneri was described in Blanchard and Knudson (1983a).

179 Acentria was transferred to the Acentropinae (=Nymphulinae) from the Schoenobiinae in Speidel (1984); see also Passoa (1988). Many Old World synonyms were not listed in Munroe (1983), but are included here.

180 Artopsis was synonymized with Parachma in Barnes and McDunnough (1917).

181 Artopsis borregalis was synonymized with Parachma ochracealis by Cashatt 2013 in Solis et al. (2013).

182 Basacallis was described as new genus for Parachma tarachodes in Cashatt (1984).

183 Polloccia was synonymized with Acallis in Cashatt (1969).

184 Acallis centralis was synonymized with Acallis gripalis by Cashatt 2013 in Solis et al. (2013).

185 Polloccia alticolalis was transferred to Acallis as a new combination by Cashatt 2013 in Solis et al. (1995).

186 Acallis mitchelli was transferred to Zaboba in Solis et al. (1995).

187 Balidarcha was synonymized with Anemosella in Cashatt (1969).

188 Anemosella polingalis was synonymized with Anemosella basalis by Cashatt 2013 in Solis et al. (2013).

189 Balidarcha cuis was synonymized with Anemosella viridalis in Munroe (1983).

190 Epitamyra albomaculalis was first reported in the United States from Florida by James Hayden in Dixon and Anderson (2014b).

191 Paragalasa was decribed as new genus with Paragalasa exospinalis as a new species by Cashatt 2013 in Solis et al. (2013).

192 Negalasa rubralis was recognized as a species in Solis et al. (1995).

193 Tetraschistis leucogramma was transferred to Penthesilea and recognized as a junior synonym of Penthesilea difficilis in Solis et al. (1995).

194 Penthesilea sacculalis baboquivariensis was described as a new subspecies by Cashatt 2013 in Solis et al. (2013).

195 Ballonicha and Teucronoma were synonymized with Salobrena in Solis et al. (1995).

196 Salobrena rubiginea was synonymized with Salobrena recurvata in Solis et al. (1995).

197 Epitamyra birectalis was transferred to Salobrena and synonymized with Salobrena vacuana in Solis et al. (1995).

198 Clydonopteron was reinstated as a separate genus from Salobrena in Cashatt (1969).

199 Clydonopteron tecomae was synonymized with Clydonopteron sacculana in Miller and Becker (1989).

200 Xantippides was synonymized with Arta in Cashatt (1969).

201 Xantippides descansalis was synonymized with Arta epicoenalis in Munroe (1983).

202 Xantippe beatifica was synonymized with Arta epicoenalis by Cashatt 2013 in Solis et al. (2013).

203 Arta brevivalvalis was described by Cashatt 2013 in Solis et al. (2013).

204 Xantippe uranides was synonymized with Heliades mulleolella by Cashatt 2013 in Solis et al. (2013).

205 Heliades lindae was described by Cashatt 2013 in Solis et al. (2013).

206 Heliades huachucalis was elevated to species status by Cashatt 2013 in Solis et al. (2013).

207 Arenipses was synonymized with Aphomia in Kuchlein (1978).

208 Paralipsa decorella was transferred to Aphomia and synonymized with Aphomia terrenella in Solis and Metz (2008).

209 Aphomia fulminalis was omitted from the Munroe (1983) list. It was included by Solis and Metz (2008).

210 Paralipsa and Corcyra were synonymized with Aphomia in Leraut (2014).

211 Athaliptis was synonymized with Epimorius in Solis [2003].

212 Epimorius testaceellus was first reported in the United States in Ferguson (1991).

213 Stenopaschia trichopteris was first reported in the United States from Texas, Cameron Co., Sabal Palm Sanctuary by Knudson (1996).

214 All species formerly in Macrotheca were placed in Cacotherapia because the former name was preoccupied. Munroe (1995b) made all new species combinations except Cacotherapia angulalis and Cacotherapia bilinealis.

215 Conobathra and Trachycera were synonymized with Acrobasis in Leraut (2005b).

216 Hylopylora and Rhodophaeopsis were synonymized with Numonia, which is a synonym of Acrobasis in Roesler (1985, 1987).

217 Cyphita was synonymized with Conobathra, which is a synonym of Acrobasis in Roesler (1985).

218 Acrobasis grossbecki was synonymized with Acrobasis indigenella in Neunzig (1986).

219 Rhodophaea kofa, Rhodophaea fria and Rhodophaea neva were all synonymized with Acrobasis comptella in Neunzig (1986).

220 Acrobasis feltella was synonymized with Acrobasis palliolella in Neunzig (1986).

221 Acrobasis kylesi, Almita texana, Acrobasis juglanivorella, and Acrobasis caulivorella were described in Neunzig (1986).

222 Acrobasis tumidulella was recognized as a species with Acrobasis kearfottella as a synonym in Leraut (2005b).

223 Acrobasis peplifera was synonymized with Acrobasis exsulella in Neunzig (1986).

224 Acrobasis malipennella and Acrobasis secundella were synonymized with Acrobasis normella in Neunzig (1986).

225 Acrobasis myricella was synonymized with Acrobasis cirroferella in Neunzig (1986).

226 Acrobasis comacornella was synonymized with Acrobasis caryivorella in Neunzig (1986).

227 Acrobasis hebescella was synonymized with Acrobasis betulella in Neunzig (1986).

228 Acrobasis dyarella was synonymized with Acrobasis rubrifasciella in Neunzig (1986).

229 Rhodophaea was synonymized with Pempelia in Leraut (1997). This genus does not apply to Rhodophaea caliginella and Rhodophaea suavella and Neunzig (1986) transferred them to Trachycera, which was subsequently synonymized with Acrobasis in Leraut (2005b).

230 Rhodophaea cruza, Rhodophaea durata, and Rhodophaea yuba were all synonymized with Trachycera caliginella in Neunzig (1986). These were then transferred to Acrobasis in Leraut (2005b).

231 Anypsipyla univitella was first reported in the United States from Florida by Dickel (1988).

232 Crocidomera imitata was described in Neunzig (1990). This entity was formerly included in the check list as turbidella Zell., 1848, but was recognized as a new species in Neunzig (1990).

233 Neunzig (1990: 34) designated as lectotype the type specimen labeled “Dioryctria minutulella (sic) Hulst” that Hulst described as minutularia in 1887 and later misspelled in 1890.

234 Myelois coniella (and thus Rampylla nefas) was synonymized with Myelopsis minutularia in Neunzig (1990).

235 Myelopsoides was described for the new species Myelopsoides venustus in Neunzig (1998).

236 Ectomyelois was synonymized with Apomyelois in Leraut (2002).

237 Fundella agapella was synonymized with Fundella argentina in Landry and Neunzig (1998).

238 Fundella ignobilis was first reported in the United States from Texas by Knudson (1983).

239 Scorylus cubensis was first reported in the United States from Florida by Dickel (1987).

240 Anegcephalesis was synonymized with Zamagiria in Neunzig (2003).

241 Philocrotona was described in Neunzig (2003) with Zamagiria kendalli as the type species.

242 Pima occidentalis was elevated to species status in Neunzig (2003).

243 Pima subcostella was no longer recognized as a subspecies in Neunzig (2003).

244 Pima vividella was synonymized with Pima fulvirugella in Neunzig (2003).

245 Pima fergusoni was described in Neunzig (2003).

246 Pimodes caliginosus was described in Neunzig (2003).

247 Epischnia ruderella was synonymized with Interjectio denticulella in Neunzig (2003).

248 Ambesa mirabella was elevated to species status in Neunzig (2003).

249 Ambesa dentifera was described in Neunzig (2003).

250 Catastia subactualis was described in Neunzig (2003).

251 Salebriaria ademptanella and Salebriaria robustella were elevated to species status in Neunzig (1988).

252 Salebriaria borealis, Salebriaria chisosensis, Salebriaria fasciata, Salebriaria rufimaculatella, Salebriaria bella, Salebriaria grandidentalis, Salebriaria equivoca, Salebriaria integra, Salebriaria maximella, Salebriaria simpliciella, Salebriaria carolynae, and Salebriaria squamopalpiella were described in Neunzig (1988).

253 Salebriaria roseopunctella, Salebriaria kanawha, Salebriaria floridana, and Salebriaria pallidella were described in Neunzig (2003).

254 Psorosina fergusonella was described in Blanchard and Knudson (1983b).

255 Psorosina fergusonella was transferred to Salebriaria in Neunzig (2003).

256 Salebriaria atratella was described in Blanchard and Knudson (1985b).

257 Salebriaria atratella, Salebriaria fructetella and Salebriaria occidentalis were transferred to Quasisalebria in Neunzig (2003).

258 Salebriaria occidentalis was described in Neunzig (1988).

259 Ortholepis baloghi was described in Neunzig (2003).

260 Meroptera anaimella was described in Blanchard and Knudson (1985c).

261 Meroptera nevadensis was described in Neunzig (2003).

262 All species of Nephopterix of the United States were transferred to Sciota in Neunzig (2003), following the European placement of Phycita rhenella in Sciota (Palm 1986; Speidel 1996; Leraut 1997).

263 Sciota fraudifera was omitted from the Munroe (1983) check list. Neunzig (2003) included this species.

264 Sciota fraudifera, Sciota floridensis, Sciota levigatella and Sciota yuconella were elevated to species status in Neunzig (2003).

265 The original spelling of this species by Hulst 1886 was tarmitalis; termitalis was incorrectly used in Munroe (1983) and Neunzig (2003).

266 Sciota californiana and Sciota quasisubfuscella were described in Neunzig (2003).

267 Telethusia rhypodella was synonymized with Telethusia ovalis in Neunzig (2003).

268 Stylopalpia luniferella was first reported in the United States from Florida by Dickel (1988) as Stylopalpia lungerella.

269 The original spelling of this name is luniferella. Both Heinrich (1956) and Munroe (1995a) incorrectly used lunigerella.

270 Hypochalcia hulstiella was synonymized with Hypochalcia ahnella, and all Palaearctic synonyms are listed in Leraut (2002).

271 Pyla gaspeensis was synonymized with Pyla aequivoca in Balogh and Wilterding (1998).

272 Pyla araeneola was described in Balogh and Wilterding (1998).

273 Many Old World synonyms of Pyla fusca were included in Neunzig (2003).

274 Pyla westerlandi was described in Wilterding and Balogh (2002).

275 Pyla longispina and Pyla serrata were described in Neunzig (2003).

276 Utah was described as a new genus in Ferris (2012a) with the type species sanrafaelensis described as a new species.

277 Dioryctroides and Ocrisia were synonymized with Dioryctria in Speidel and Asselbergs (2000).

278 Dioryctria hodgesi, Dioryctria mutuurai, Dioryctria muricativorella, Dioryctria inyoensis and Dioryctria sierra described in Neunzig (2003).

279 Dioryctria resinosella was described in Mutuura (1982).

280 Dioryctria delectella and Dioryctria vancouverella were elevated to species status in Neunzig (2003).

281 Dioryctria yatesi, Dioryctria ebeli and Dioryctria merkeli were described in Mutuura and Munroe (1979).

282 Dioryctria westerlandi and Dioryctria fordi were described in Donahue and Neunzig [2005].

283 Dioryctria durangoensis was described in Mutuura and Neunzig (1986).

284 Dioryctria caesirufella was described in Blanchard and Knudson (1983c).

285 Dioryctria taedivorella was described in Neunzig and Leidy (1989).

286 Sarata septentrionaria was synonymized with Sarata punctella in Neunzig (2003). It is no longer considered a subspecies.

287 Sarata alpha is probably the female of Sarata caudellella (Landry 1998); names of unassociated females are not treated in Neunzig (2003).

288 Lipographis leoninella was synonymized with Lipographis fenestrella in Neunzig (2003).

289 Lipographis unicolor was described in Ferris (2012b).

290 Quasisarata was described in Neunzig (2003). Lipographis subosseella is the type species.

291 Quasisarata subosseella was first reported in the United States from Florida by Dickel (1987) in the genus Lipographis.

292 Pseudadelphia was described in Neunzig (2003).

293 Ufa roseitinctella was synonymized with Ufa lithosella in Neunzig (2003).

294 Acroncosa minima was described in Neunzig (2003).

295 Acroncosa castrella was elevated to species status in Neunzig (2003).

296 Passadenoides was described for the new species Passadenoides donahuei and Passadenoides pullus in Neunzig (2003).

297 Passadenoides montanus was described in Ferris (2004).

298 Chorrera extrincica was first reported in the United States from Texas by Knudson (1983). Neunzig (2003) stated that this species “has been found on two of the Florida Keys (Long Key and Grassy Key, Monroe Co.).

299 Eumysia pallidipennella was omitted from the Munroe (1983) list, but was listed by Shaffer (1995).

300 Arcola malloi was first reported from the United States in Brown and Spencer (1973). It was introduced to control alligatorweed (Coulson 1977) and was omitted from the Munroe (1983) list.

301 Ocala and Divitiaca were synonymized with Macrorrhinia in Neunzig (2003).

302 Divitiaca simulella was synonymized with Macrorrhinia ochrella in Neunzig (2003).

303 Shaffer (1991) transferred Rhinaphe endonephele and Rhinaphe ignetincta to Divitiaca (=Macrorrhinia).

304 Macrorrhinia endonephele was established as an older name for Macrorrhinia ignetincta and Macrorrhinia signifera, both new synonyms, in Neunzig (2003).

305 Maricopa was recognized as the valid genus name because Valdivia is preoccupied (Shaffer 1995).

306 Protasia mirabilicornella, described in Valdivia from California, was omitted from Munroe (1983), but listed in Shaffer (1995). It was also reported in Texas by Bordelon and Knudson (2000).

307 Staudingeria was synonymized with Ancylosis in Leraut (2008).

308 Pseudocabotia was described for the new species Pseudocabotia balconiensis in Blanchard and Knudson (1985c).

309 Pseudocabotia was synonymized with Ancylosis in Hayden and Dickel (2014).

310 Ancylosis bonhoti was first reported in the United States from Florida by Hayden and Dickel (2014).

311 Palatka powelli was described in Neunzig and Solis (1996).

312 Anderida peorinella was described in Blanchard and Knudson (1985b).

313 Cassiana malacella was first reported in the United States from the Florida in Neunzig (1997).

314 Mescinia berosa was reported in United States from Florida in Neunzig (1997).

315 Mescinia parvula was reported in the United States from Florida in Neunzig (1997).

316 Mescinia neoparvula was synonymized with Mescinia parvula in Neunzig (1997).

317 Mescinia texanica was described in Neunzig (1997).

318 Phestinia costella was discovered in the United States when it was submitted for identification (Solis 2011) for R. Diaz (Diaz et al. 2015). See also Solis et al. (2008) for information about the identity of this species.

319 Comotia torsicornis was reported for the first time in the United States from Florida by Heppner and Dickel (2008).

320 Homoeosoma phaeoboreas, Homoeosoma asylonnastes, Homoeosoma oxycercus, Homoeosoma ammonastes, Homoeosoma pedionnastes, Homoeosoma eremophasma, and Homoeosoma ardaloniphas were described in Goodson and Neunzig (1993).

321 Homoeosoma breviplicitum and Homoeosoma imitator were synonymized with Homoeosoma striatellum in Goodson and Neunzig (1993).

322 Homoeosoma parvalbum was described in Blanchard and Knudson (1985c).

323 Homoeosoma emendator was elevated to species status in Goodson and Neunzig (1993).

324 Homoeosoma nanophasma was described in Neunzig (2003).

325 Phycitodes albatella is no longer recognized from North America; it occurs only in the Old World.

326 Phycitodes mucidella and Phycitodes reliquellum were elevated to species status by Neunzig (1997).

327 Unadilla floridensis was synonymized with Unadilla maturella in Neunzig (1997).

328 Unadilla nasutella was synonymized with Unadilla erronella in Neunzig (1997).

329 Laetilia dilatifasciella and Laetilia hulstii were elevated to species status in Neunzig (1997).

330 Laetilia cinerosella and Laetilia bellivorella were described in Neunzig (1997).

331 Dakruma pallida was transferred from Laetilia as a new name for Baphala basimaculatella in Neunzig (1997).

332 Baphala eremiella was elevated to species status in Neunzig (1997).

333 Baphala phaeolella was described in Neunzig (1997).

334 Zophodia multistriatella was described in Blanchard and Knudson (1981).

335 Olycella was synonymized with Melitara in Neunzig (1997).

336 Melitara texana was described in Neunzig (1997).

337 Melitara doddalis was elevated to species status in Neunzig (1997).

338 Melitara apicigrammella was described in Blanchard and Knudson (1985c).

339 Zophodia holochlora was synonymized with Alberada bidentella in Neunzig (1997).

340 Alberada californiensis, Alberada franclemonti, and Alberada candida were described in Neunzig (1997).

341 Cactoblastis cactorum was first reported in the United States from Florida in Habeck and Bennett (1990).

342 Rumatha jacumba was described in Neunzig (1997).

343 Zophodia longipennella was synonymized with Yosemitia graciella in Neunzig (1997).

344 Ozamia clarefacta was elevated to species status in Neunzig (1997).

345 Ozamia lucidalis was first reported in the United States from Florida in Habeck and Bennett (1990).

346 Echinocereta was described in Neunzig (1997).

347 Selga californica was described in Neunzig (1990).

348 Pseudocabima arizonensis was omitted from Munroe (1983), but included by Neunzig (1990).

349 Euzophera aglaeella was elevated to species status in Neunzig (1990).

350 Euzophera habrella and Euzophera vinnulella were described in Neunzig (1990).

351 Ephestiodes monticolus and Ephestiodes griseus were described in Neunzig (1990).

352 Australephestiodes was described in Neunzig (1990).

353 Australephestiodes stictella was first reported in the United States from Florida in Neunzig (1990).

354 Moodnodes was described in Neunzig (1990).

355 Moodnodes plorella was first reported in the United States from Florida in Neunzig (1990).

356 Moodna pallidostrinella was described in Neunzig (1990).

357 Manhatta was synonymized with Vitula in Roesler (1973); also see Neunzig (1990).

358 Vitula serratilineella was determined to be an older name for Vitula bombylicolella by Sattler (1989).

359 Vitula serratilineella was elevated to species status in Neunzig (1990).

360 Vitula aegerella, Vitula insula, and Vitula coconinoana were described in Neunzig (1990).

361 Volatica gallivorella was described in Neunzig (1990).

362 Caudellia albovittella was synonymized with Caudellia apyrella in Neunzig (1990).

363 Caudellia floridensis was described in Neunzig (1990).

364 Sosipatra proximanthophila and Sosipatra knudsoni were described in Neunzig (1990).

365 Heinrichiessa was described for the new species Heinrichiessa sanpetella in Neunzig (1990).

366 Ribua droozi was described in Neunzig (1990).

367 Ribua innoxia is here reported for the first time in the United States from South Carolina by BGS. A male specimen deposited in the BGS personal collection was collected from Francis Marion NF, Berkeley Co., 7 Sep 2002. A somewhat rubbed male with limited reddish scales, but vinculum distinctly twice as long as wide (see Neunzig 1990 for comparison with Ribua droozi).

368 Bethulia championella was first reported in the United States Neunzig and Solis (2006).

369 Anagasta was synonymized with Ephestia in Neunzig (1990).

370 Homoeosoma ischnomorpha was synonymized with Ephestia kuehniella in Neunzig (1990).

371 Ephestia columbiella was described in Neunzig (1990).

372 Tinea aquella is a junior subjective synonym and is an undisputed available name and an unused senior synonym as per Article 23 in the International Code of Zoological Nomenclature (K. Sattler pers. comm.; see also Sattler and Tremewan 1984 on Denis & Schiffermüller names).

373 Ephestia pterogrisella was synonymized with Ephestia elutella in Neunzig (1990).

374 Uncitruncata was described for the new species Uncitruncata leuschneri in Neunzig (2000).

375 Eurythmia furnella was misspelled as fumella in Heinrich (1956) and Munroe (1983).

376 Eurythmia spaldingella and Eurythmia yavapaella were elevated to species status in Neunzig (1990).

377 Bema neuricella was first reported in the United States from Florida by Dickel (1987).

378 Peoria padreella was described in Blanchard (1980).

379 Peoria insularis was described in Shaffer (2003).

380 Atascosa heitzmani was described in Shaffer (1980).

381 Epipaschia zelleri was transferred to Macalla in Solis (1992).

382 Epipaschia albomedialis was transferred to Cacozelia as a synonym of Cacozelia pemphusalis in Solis (1993).

383 Jocara interruptella and Jocara elegans were transferred to Cacozelia in Solis (1993).

384 Tioga neotropica was synonymized with Cacozelia elegans in Solis (1993).

385 Cacozelia alboplagialis was transferred to Milgithea in Solis (1993).

386 Milgithea trilinearis was reported as reared in the United States from Florida in Solis (1993).

387 Toripalpus was resurrected as a genus for Jocara breviornatalis and Jocara trabalis in Solis (1993).

388 Yuma was omitted from Solis (1993, 1995).

389 The synonymy of Toripalpus adulatalis was omitted from Solis (1993, 1995).

390 Deuterollyta was resurrected as a genus to include Jocara incrustalis as a synonym of Deuterollyta majuscula in Solis (1993).

391 Jocara perseella was synonymized with Deuterollyta majuscula in Solis (1993).

392 Oneida diploa and Oneida pallidalis were synonymized with Oneida luniferella in Solis (1991).

393 Oneida grisiella was described in Solis (1991).

394 Tetralopha and all its synonyms were synonymized with Pococera in Solis (1993).

395 Salebria nigricans is here transferred to Pococera as a new synonym of Pococera fuscolotella based on MAS examination of type specimen and genitalia slide.

396 Pococera tertiella was synonymized with Pococera gelidalis in Solis (1993).

397 Tallula beroella is here reported for the first time from Arizona by MAS. Specimens deposited in the NMNH with the following data: Madera Canyon, Santa Rita Mtns., Santa Cruz Co., J. G. Franclemont, various specimens collected in June and July at elevations around 5000’; also one specimen with the same data collected by D. Davis.

398 Phidotricha was resurrected as a genus to include Phidotricha erigens with Auradisa dryospila as a synonym in Solis (1993). This species was historically misidentified in the literature under the name Pococera atramentalis Lederer, 1863.

399 Macalla glastianalis was listed as Incertae sedis in Solis (1993). It requires a new genus, inclusion of several Neotropical species, and description of new species. It was first collected by A. Blanchard in Texas in the early 1970’s (MAS unpub.).

400 Several new synonyms for Pyralis farinalis and Pyralis manihotalis were assigned in Shaffer and Solis (1995) and Nuss et al. (2003–2015).

401 Euclita was synonymized with Aglossa in Fletcher and Nye (1984).

402 Oryctocera, Crocalia, Agriope and were synonymized with Aglossa in Leraut (2003b).

403 Pyralis costiferalis, Pyralis disciferalis, Pyralis electalis, and Pyralis cacamica were transferred to Aglossa in Shaffer and Solis (1995).

404 Several new synonyms for Aglossa pinguinalis and Aglossa caprealis were assigned in Shaffer and Solis (1995) and Nuss et al. (2003-2015).

405 Dolichomia, Herculia, Cisse, Buzala, Ocrasa, Bejuda, Pseudasopia, and Orthopygia were synonymized with Hypsopygia in Leraut (2006a).

406 Bleone was synonymized with Hypsopygia in Leraut (2003a).

407 Parasopia was synonymized with Hypsopygia in Shaffer and Solis (1995).

408 Pyralis intermedialis, Asopia binodulalis, Pyralis nostralis, and Pyralis olinalis were transferred to Hypsopygia in Leraut (2006a).

409 Pyralis nostralis was placed in Ocrasa (=Hypsopygia) in Shaffer and Solis (1995).

410 Parasopia dissimilalis, Ocrasa sordidalis, Herculia psammioxantha, and Herculia venezuelensis were synonymized with Dolichomia (=Hypsopygia) nostralis in Shaffer and Solis (1995).

411 Herculia infimbrialis was synonymized with Dolichomia (=Hypsopygia) olinalis in Shaffer and Solis (1995).

412 Uscodys was synonymized with Arispe in Shaffer and Solis (1995).

413 Uscodys atalis was synonymized with Arispe cestalis in Shaffer and Solis (1995).

414 Neodavisia melusina was described in Ferguson et al. (1984).

415 Austromusotima camptozonale was released but not established in Florida (Boughton and Pemberton 2012).

416 Blepharocerus rosellus and Blepharocerus ignitalis were evidently inserted into the MONA list inadvertently by Munroe (1983). They had not been listed in any previous check lists, and we have found no documented records. Both species are described from Chile.

417 Solis and Metz (2008) indicated that Aphomia fuscolimbella is likely not of North American origin.

418 Although covered in Neunzig (1986), he mentions that Cryptoblabes gnidiella is still not recorded from North America, but it is known from Bermuda, Central and South America, and Hawaii.

419 Although included in Munroe (1983), there is no evidence to indicate that Apomyelois decolor occurs in the United States, and Neunzig (1990) did not include this species.

420 Myelois grossipunctella was reported from the United States but not established and not reported again (Shaffer et al. 1996).

421 Myelois grossipunctella was found to be a senior synonym of Creobota coccophthora by Shaffer et al. (1996) transferring grossipunctella to Creobota.

422 Homoeosoma pseudonimbella was found to be a synonym of Phycitodes albatella by Leraut (2002).

423 Cactobrosis longipennella was removed from United States faunal list in Neunzig (1997).

424 Vitula lugubrella was found to be a synonym of Vitula biviella, a European species. The California description is believed to be in error according to Neunzig (1990).

425 Coenodomus hockingi was included in Munroe (1983) based on the description of Dyaria singularis from Maine. This locality is now believed to be either in error, or a one time introduction that was not established, and this species is removed from the check list (Solis 1992).

426 Pococera atramentalis Lederer, 1863 was included in Munroe (1983) as a result of misidentifications in the literature (see Note #396).

427 Hemimatia atramentalis was transferred to Tallula in Solis (1993). Tallula atramentalis (Lederer, 1863)(Hemimatia), described from Venezuela, does not occur in the U.S.
